# A potential EARLY FLOWERING 3 homolog in *Chlamydomonas* is involved in the red/violet and blue light signaling pathways for the degradation of RHYTHM OF CHLOROPLAST 15

**DOI:** 10.1371/journal.pgen.1010449

**Published:** 2022-10-17

**Authors:** Malavika Gururaj, Ayumi Ohmura, Mariko Ozawa, Takashi Yamano, Hideya Fukuzawa, Takuya Matsuo

**Affiliations:** 1 Center for Gene Research, Nagoya University, Nagoya, Japan; 2 Graduate School of Science, Nagoya University, Nagoya, Japan; 3 Graduate School of Biostudies, Kyoto University, Kyoto, Japan; Peking University, CHINA

## Abstract

Light plays a major role in resetting the circadian clock, allowing the organism to synchronize with the environmental day and night cycle. In *Chlamydomonas* the light-induced degradation of the circadian clock protein, RHYTHM OF CHLOROPLAST 15 (ROC15), is considered one of the key events in resetting the circadian clock. Red/violet and blue light signals have been shown to reach the clock via different molecular pathways; however, many of the participating components of these pathways are yet to be elucidated. Here, we used a forward genetics approach using a reporter strain that expresses a ROC15-luciferase fusion protein. We isolated a mutant that showed impaired ROC15 degradation in response to a wide range of visible wavelengths and impaired light-induced phosphorylation of ROC15. These results suggest that the effects of different wavelengths converge before acting on ROC15 or at ROC15 phosphorylation. Furthermore, the mutant showed a weakened phase resetting in response to light, but its circadian rhythmicity remained largely unaffected under constant light and constant dark conditions. Surprisingly, the gene disrupted in this mutant was found to encode a protein that possessed a very weak similarity to the *Arabidopsis thaliana* EARLY FLOWERING 3 (ELF3). Our results suggest that this protein is involved in the many different light signaling pathways to the *Chlamydomonas* circadian clock. However, it may not influence the transcriptional oscillator of *Chlamydomonas* to a great extent. This study provides an opportunity to further understand the mechanisms underlying light-induced clock resetting and explore the evolution of the circadian clock architecture in Viridiplantae.

## Author summary

Resetting of the circadian clock is crucial for an organism, as it allows the synchronization of its internal processes with the day/night cycle. Environmental signals—such as light and temperature—contribute to this event. In plants, the molecular mechanisms underlying the light-induced resetting of the circadian clock have been well-studied in the streptophyte, *Arabidopsis thaliana*, and has been explored in some chlorophyte algae such as *Ostreococcus tauri* and *Chlamydomonas reinhardtii*. Here, we used a forward genetics approach to examine the light signaling pathway of a process considered critical for the light resetting of the *Chlamydomonas* clock—light-induced degradation of the circadian clock protein ROC15. We explored various aspects of the isolated mutant, such as the degradation of ROC15 in response to a range of visible wavelengths, the circadian rhythm, and the phase resetting of the rhythm. We show that the effects of different wavelengths of light converge before acting on ROC15 or at ROC15 phosphorylation with the aid of a potential homolog of the *Arabidopsis thaliana* ELF3. Our findings contradict the existing view that there is no known homolog of ELF3 in chlorophyte algae. This study, therefore, sheds light on the evolutionary aspects of the Viridiplantae circadian clocks and their light resetting.

## Introduction

Most organisms on Earth show circadian rhythms, which are characterized by the three following features: 1) the period of the rhythm is approximately 24 h, even under constant environmental conditions; 2) the rhythm is temperature-compensated (i.e., the period of the rhythm does not change significantly with changes in ambient temperature); and 3) the phase of the rhythm can be reset (advanced or delayed) by external cues (e.g., light and temperature), which allows the organism to synchronize its activity with the environmental day/night cycle. Circadian rhythms are thought to be generated and maintained by a molecular circadian clock consisting of three parts: input pathways, an oscillator, and output pathways. The oscillator generates the rhythm, whereas the input pathways connect the oscillator to external resetting cues. These input pathways have been explored at the molecular level in many model organisms. Such investigations have resulted in the identification of photoreceptors involved in receiving light for resetting and the molecular events downstream of light reception (e.g., the induction of clock gene expression and the turnover of clock protein, among others) [1,2,3]

The molecular pathway underlying light-induced resetting of the circadian rhythm has been investigated in plant model organisms such as *Arabidopsis thaliana* [4]. The molecular components involved in integrating light signals and the molecular clock, include the protein EARLY FLOWERING 3 (ELF3) [4]. ELF3 plays a role in the circadian oscillator as part of the Evening Complex (EC) along with either of the following GARP transcription factors: LUX ARRHYTHMO (LUX) or BROTHER OF LUX (BOA) and the protein EARLY FLOWERING 4 (ELF4) [5,6,7]. Interestingly, the *elf3* mutant shows an abnormal light resetting of the clock and a loss of circadian gating in the light induction of a clock-controlled *CHLOROPHYLL A/B-BINDING PROTEIN 2* gene, therefore, ELF3 is suggested to play a significant role in the light input pathway to the circadian clock [8, 9]. In addition, ELF3 is known to interact with the phytochrome photoreceptors, Phy A-E [10,11]. The direct interaction between ELF3 and CONSTITUTIVE PHOTOMORPHOGENIC 1 (COP1) is also thought to connect the clock to many light-signaling cascades, as COP1 is a common factor in these pathways [4,10,12].

Light resetting at the molecular level has also been investigated in the prasinophyte green alga, *Ostreococcus tauri*. This alga possesses a histidine kinase with a light, oxygen, and voltage sensing domain photoreceptor (LOV-HK), which is involved in circadian clock functions [13]. Studies using experimental and mathematical modeling approaches have suggested that this LOV-HK photoreceptor—along with the other histidine kinase, Rhodopsin-HK—participates in a two-component signaling system to reset the *O*. *tauri* circadian clock using blue and green light [14].

The action spectrum for the resetting of the circadian rhythm has been investigated in the unicellular chlorophyte alga, *Chlamydomonas reinhardtii* [15,16]. A series of phase shift experiments over a broad range of wavelengths revealed that for dark-adapted *C*. *reinhardtii*, the phase resetting was sensitive to many wavelengths, especially to green and red wavelengths (i.e., 520 nm and 660 nm, respectively) [15]. It was later demonstrated that blue light (440 nm) could also reset the clock with high efficacy in another strain of *C*. *reinhardtii* [17]. Therefore, many photoreceptors have been considered as candidates for those in resetting the circadian clock. Some of the candidates for responses to blue and/or green light include phototropin, rhodopsins, and cryptochromes [18]. Of these, the plant-like cryptochrome which plays a role in the oscillator is also involved in the input pathway of the *C*. *reinhardtii* circadian clock [17,19]. Candidates for responses to red light include the animal-like cryptochrome, which has been shown to absorb red light in its dark form (the neutral radical state of flavin chromophore) in addition to absorbing blue light (oxidized state of flavin chromophore), and to regulate the blue and red light responses of some clock genes [20]. Interestingly, no known homolog of the phytochrome photoreceptor family, which is known to be involved in the red light response of the circadian clock in *A*. *thaliana* [21,22,23], has been found in *C*. *reinhardtii* [24,25].

The identification of the clock gene, *RHYTHM OF CHLOROPLAST 15* (*ROC15*) [26], has shed light on the molecular mechanisms of light resetting in *Chlamydomonas*. The encoded protein, ROC15, possesses a GARP DNA-binding motif similar to that of LUX and BOA (part of the EC in *A*. *thaliana*) [26]. Not only does ROC15 undergo circadian-phase-independent light-induced degradation, an insertional mutant of this gene also shows abnormal light resetting, as it fails to show a phase shift in response to a light pulse [27]. In addition, ROC15 degradation—similar to clock resetting—is sensitive to wavelengths across the entire visible spectrum [27,28]. Therefore, the light-induced degradation of ROC15 has been suggested to be associated with light resetting in *C*. *reinhardtii* [27,28]. The isolation of a mutant of *C*. *reinhardtii SHOC2/SUR8-like leucine-rich repeat* (*CSL*) revealed the existence of at least two pathways (i.e., red/violet and blue light pathways) by which light information was communicated to the clock. This was because the mutant showed an impaired response of ROC15 to red and violet light, but not to blue light [28].

However, although these findings have started to reveal the molecular pathways underlying light resetting in *C*. *reinhardtii*, there are still many questions that need to be answered. In this study, to further elucidate the molecular mechanisms of light resetting in *C*. *reinhardtii*, we used the ROC15-LUC reporter strain [27], which expresses a fusion protein of ROC15 and firefly luciferase. We isolated a mutant that showed an impaired ROC15 light response over a wide range of wavelengths. We then characterized this mutant to understand the significance of the gene disrupted in this mutant in the circadian oscillator and light resetting mechanism.

## Results

### Screening for mutants of ROC15 light response

**Fig 1:**
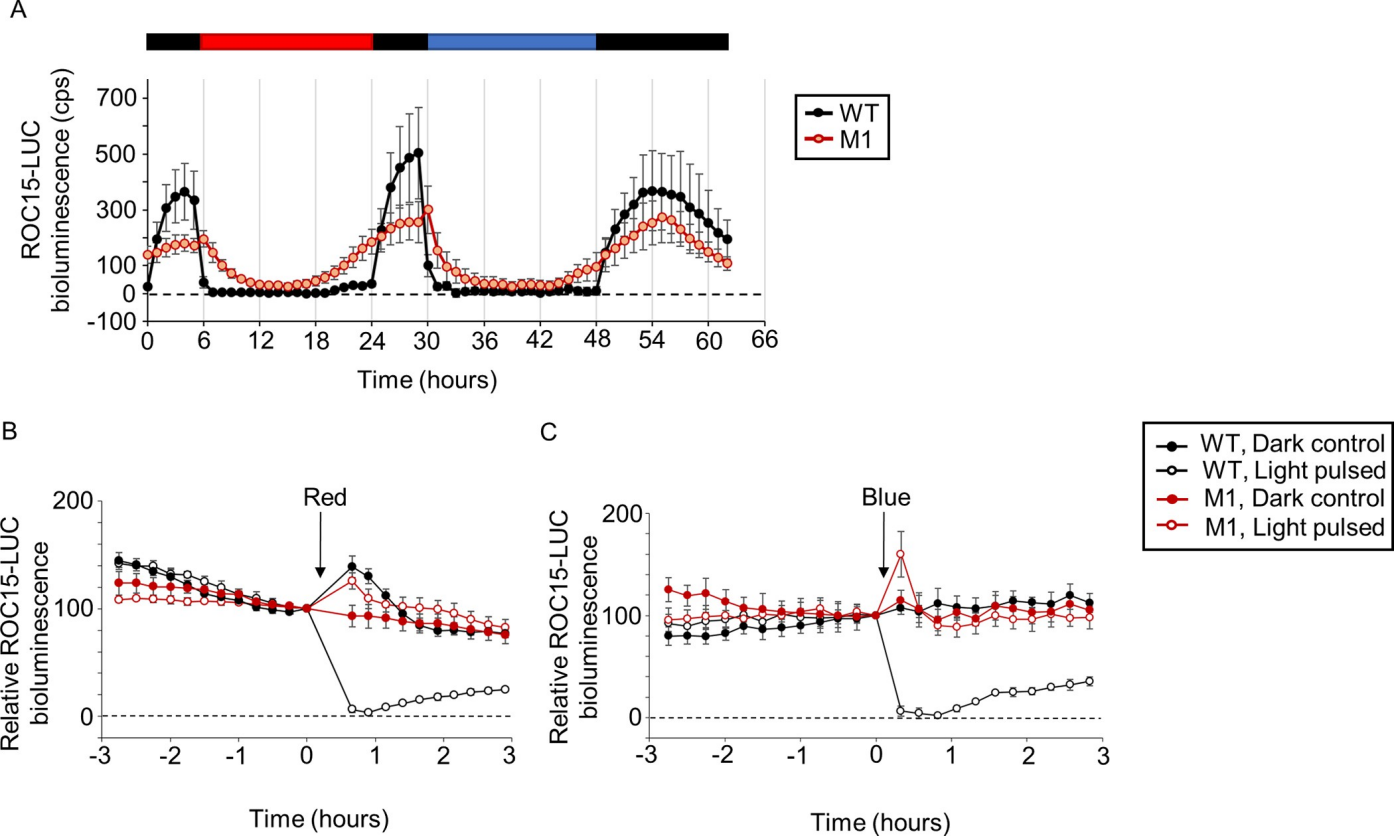
Representative bioluminescence traces of the M1 mutant. **A**. ROC15-LUC bioluminescence pattern of M1 under diurnal conditions. The red, blue, and black bars above the graph represent red light (8 μmol m^-2^ s^-1^), blue light (20 μmol m^-2^ s^-1^), and darkness, respectively. Cells were prepared for the screening as described in Materials and Methods. Mean ± standard deviation (SD) of 32–36 biological replicates are plotted. **B** and **C.** Representative trace of the ROC15-LUC bioluminescence response to light pulses in M1 mutant. Unsynchronized TAP cultures were transferred into 24 well black plates, maintained in darkness for at least 6 hours to allow for the accumulation of ROC15-LUC, and exposed to a 5 min pulse of red light (**B**, 10 μmol m^-2^ s^-1^) or blue light (**C**, 20 μmol m^-2^ s^-1^). Bioluminescence has been calculated relative to that at the time point just before light pulse (Time 0). Background was not subtracted. Mean ± SD of 6 biological replicates are shown. Arrows indicate the approximate time points of light pulses. The increase in bioluminescence levels after the light pulse probably corresponds to the disturbance of the plate during the experiment.

Mutants were generated by random insertional mutagenesis of the ROC15-LUC reporter strain using the hygromycin resistance gene, *aph7”* [29]. Approximately 4700 transformants were subjected to two cycles of a 6 h dark/18 h light schedule (one red and one blue light cycle). Under these conditions, the wild type (WT) ROC15 bioluminescence levels increased in the dark period, decreased acutely at the start of light-on conditions, and remained low until the end of the light period (**Fig 1A**, WT). These patterns reflected the expression levels of ROC15 [27]. Two mutants (tentatively named M1 and M2) were isolated. The first mutant (M1) showed a gradual decrease instead of an acute one in ROC15 bioluminescence levels after the transition to the light phase (**Fig 1A**). In addition, M1 bioluminescence levels failed to remain low until the start of the dark phase (**Fig 1A**). These altered patterns of ROC15-LUC bioluminescence were observed in both the red and blue cycles of screening (**Fig 1A**). The ROC15 light response in the mutant was further examined by exposing M1 to 5-min pulses of red and blue light. Unlike the WT, M1 failed to show an acute decrease in ROC15 bioluminescence levels in response to both light pulses (**Fig 1B and 1C**). Taken together, these results suggested that the light-dependent degradation of ROC15 was affected in the mutant. The second mutant (M2) also showed an altered ROC15 bioluminescence response to both red and blue lights, similar to the M1 phenotype (**S1 Fig**). In conclusion, we were able to isolate two light response mutants that showed impaired ROC15 bioluminescence responses to both red and blue light.

### Gene disrupted in the M1 mutant

To identify the disrupted genes in the mutants, we performed a genetic linkage analysis to confirm that the mutant phenotype was linked to hygromycin resistance. This was verified by backcrossing the mutants with the parental WT strain (ROC15-LUC reporter strain). In mutant M1, all hygromycin-resistant progeny failed to show an acute decrease in ROC15 bioluminescence after light-on, whereas all the hygromycin-sensitive progeny showed an acute decrease (**S2A and S2B Fig**). These results confirmed the genetic linkage of hygromycin resistance to the mutant phenotype. However, this pattern was not observed in the second mutant (M2) (**S2C and S2D Fig**). Therefore, we continued our investigations only in the M1 mutant.

**Fig 2:**
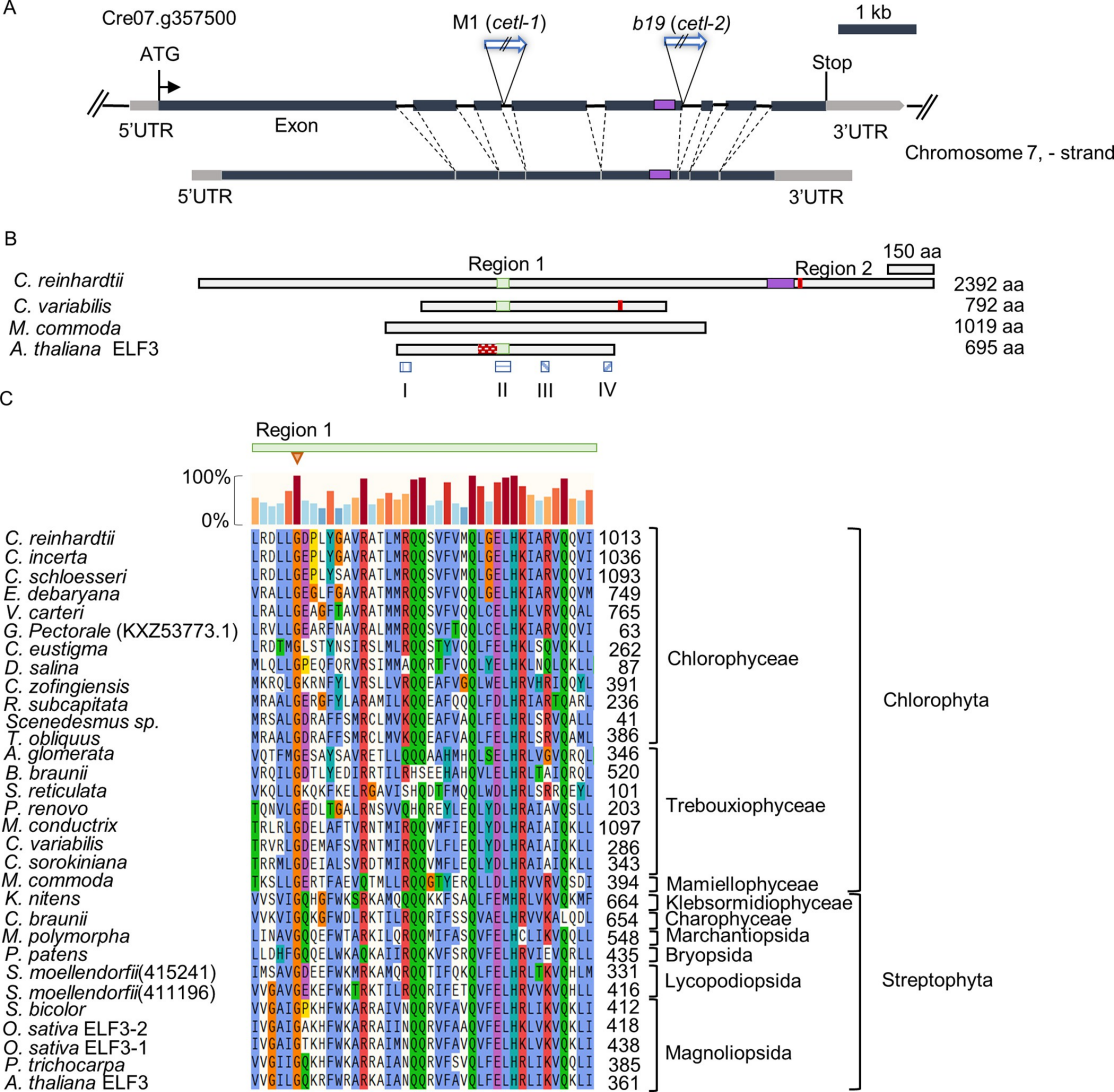
Gene responsible for the mutant phenotype. **A.** Schematic representation of Cre07.g357500 and its corresponding transcript. 5’UTR and 3’UTR ends of the transcript are illustrated based on information from the database (*C*. *reinhardtii* v5.6, Joint Genome Institute). This gene corresponds to Cre07.g800875 in the latest version (v6.1). The N-terminus appears to be missing in the gene prediction of this version. M1 (*cetl-1*) and *b19* (*cetl-2*) indicate the insertion loci of *aph7”* in the mutants. The purple box indicates the location of the additional 264 nucleotides present in our laboratory strain. The location of these additional nucleotides in the transcript and the location of the corresponding amino acids in the encoded protein are also indicated by a purple box. **B.** Protein sequence alignment. A schematic depiction of amino acid sequences, representing the sequences from the two phyla used for alignment. The two regions of similarity (Region 1 and Region 2) are also shown. The patterned red box on the *Arabidopsis* ELF3 sequence represents the region that was shown to be responsible for interaction with ELF4 [33]. The patterned blue boxes beneath the *Arabidopsis* ELF3 sequence represents the regions identified as Block I, II, III and IV [11]. **C.** Alignment of the amino acid sequences at Region 1. The amino acids have been colored according to the Clustal X -scheme i.e. according to their properties and conservation. The red inverted triangle indicates conservation at the Glycine residue, which was found to be substituted in the *elf3-12* mutant [32]. The colored bars above the alignment indicate the percentage of conservation. Sequences were aligned using MAFFT multiple sequence alignment program (MAFFT 7.471, SnapGene). The details of the amino acid sequences used for the alignment are listed in S2 Table.

In M1, the DNA flanking the hygromycin resistance marker was amplified by thermal asymmetric interlaced–polymerase chain reaction (TAIL-PCR) [30]. This sequence was compared to the *C*. *reinhardtii* reference genome (*C*. *reinhardtii* v5.6, Joint Genome Institute), and the Cre07.g357500 gene on chromosome 7 was found to be disrupted (Corresponds to the ID Cre07.g800875 in the latest version (v6.1)). This gene appeared to have eight exons, and the insertion of the hygromycin marker was found to be in the third intron (**Fig 2A**). The insertion was confirmed by PCR using a gene-specific primer pair (**S3A and S3B Fig**). We further performed a reverse transcription-PCR (RT-PCR) on the transcript (**S3A Fig**). No band with a WT band size was detected in the mutant. The two bands detected could possibly correspond to mutant transcripts resulting from unexpected alternative splicing due to the insertion of the marker (**S3C Fig**). We also performed a sequencing analysis of the RT-PCR product of the entire coding sequence (CDS) of this gene from our laboratory WT strain. The results revealed that compared to the database sequence (v5.6), our laboratory strain had an in-frame 264 nucleotide insertion in the fifth exon (**Fig 2A,** purple box). Mutants with similar phenotypes (i.e., impaired ROC15 light response to both red and blue wavelengths) had been isolated previously (*b19* and *b20* mutants) [28]. TAIL-PCR analysis of *b19* revealed that the same gene (Cre07.g357500) was disrupted (**Fig 2A**). The insertion locus of the hygromycin marker was in the fifth intron in the *b19* mutant (**Figs 2A, S3A, and S3B**).

To confirm that the disruption of the Cre07.g357500 gene caused the altered response to light in mutants, the M1 mutant was transformed with the WT gene (**S4A Fig**) to determine whether the ROC15 light response would be restored to WT levels. A total of 654 transformants (414 transformed with the WT gene and 240 transformed with only the antibiotic resistance cassette as a control group) were subjected to two cycles of a 6 h dark/18 h light schedule, with the first and second light phases consisting of red and blue light, respectively (**S4B Fig**). The ROC15 light response to both blue and red wavelengths was restored to WT levels in 6 of 414 transformants that had been transformed with the WT Cre07.g357500 gene (i.e., ROC15 bioluminescence levels declined steeply in response to light) (**S4B and S4C Fig**). The complementation rate was comparable to the rates observed in other mutants in previous studies [26]. In contrast, 0 of the 240 control transformants showed a WT ROC15 light response (**S4D Fig**). The ROC15 light responses of the six complements were further confirmed via 5-min pulses of red and blue light. The ROC15 bioluminescence levels decreased acutely in response to red and blue light pulses in all six complements (**S4E and S4F Fig**). These results confirmed that the disruption of the Cre07.g357500 caused the altered response to light in the mutant.

### Protein sequence alignment

The longest open reading frame of the Cre07.g357500 gene of our laboratory strain encodes a protein with an expected length of 2392 amino acids. This protein has not been well characterized in *Chlamydomonas*. Therefore, we performed a BLAST search of this protein sequence against Archaeplastida proteins via the algal multi-omics portal, PhycoCosm [31]. Using the default BLAST settings (e-value threshold = 1 × 10^−5^), homologs were detected only in members of Chlorophyceae. Some of the organisms that showed homologs included other species of *Chlamydomonas* (such as *C*. *incerta* and *C*. *schloesseri*), other members of the order Chlamydomonadales (such as *Volvox carteri*, *Gonium pectorale*, and *Edaphochlamys debaryana*), and organisms belonging to the order Sphaeropleales (such as *Scenedesmus obliquus*). BLAST was performed once again with a lower E-value threshold (= 1). This resulted in hits in many more chlorophytes, including members of Trebouxiophyceae (such as *Chlorella sorokiniana*), Mamiellophyceae (such as *Micromonas commoda*), and members of another phylum—Streptophyta, such as *Selaginella moellendorfii* and *Populus trichocarpa*. Surprisingly, the streptophyte proteins included homologs of the *A*. *thaliana* ELF3. Additionally, a BLAST analysis of *A*. *thaliana* ELF3 against the *C*. *reinhardtii* proteome (v5.6_281) revealed the protein encoded by Cre07.g357500 as the best hit with an E-value of 0.016. Similarly, a BLAST analysis of the protein encoded by the gene Cre07.g357500 against the *A*. *thaliana* Araport11 protein sequences database, revealed ELF3 as the best hit with an E-value of 0.11. We then aligned 33 sequences (**S2 Table**) obtained from the BLAST searches and the ELF3 homologs of the major species in Streptophyta. The results revealed two regions of similarity (Region 1 and Region 2) (**Figs 2B, 2C, and S5**). Region 2 was found only in chlorophyte proteins (**S5 Fig**), whereas Region 1 was found in the proteins of both chlorophytes and streptophytes (**Fig 2B and 2C**). Region 1 happened to be part of a region in *Arabidopsis* ELF3 referred to as Block II, which is one of the four highly conserved regions (the other three being Block I, Block III and Block IV) in angiosperm ELF3 [11]. To further observe the conservation at all the Blocks, the protein encoded by Cre07. g357500 was aligned with *A*. *thaliana* ELF3 and its homologs from only other angiosperms using MAFFT and MUSCLE multiple sequence alignment algorithms. It was observed that while Block II aligned to the same sequence of the Cre07. g357500 protein in both algorithms, Blocks I, III, and IV aligned to slightly different sequences depending on the algorithm used (**S6 Fig**). This data only weakly suggests the possibility of Cre07.g357500 being a homolog of ELF3. However, it is important to note that the Glycine residue (Gly-326) in Region 1, which when mutated is known to affect the circadian function of ELF3 [32], was fully conserved in the proteins analyzed (**Fig 2C**). In addition, Region 1 was immediately adjacent to the region that has been shown to interact with ELF4 in *Arabidopsis* [33] (**Fig 2B,** patterned red box). Therefore, we hereafter refer to this gene as *C**hlamydomonas-**E**LF**3**-**l**ike* (*CETL*), and to the M1 and *b19* mutants as *cetl-1* and *cetl-2*, respectively.

### CETL expression analyses

**Fig 3:**
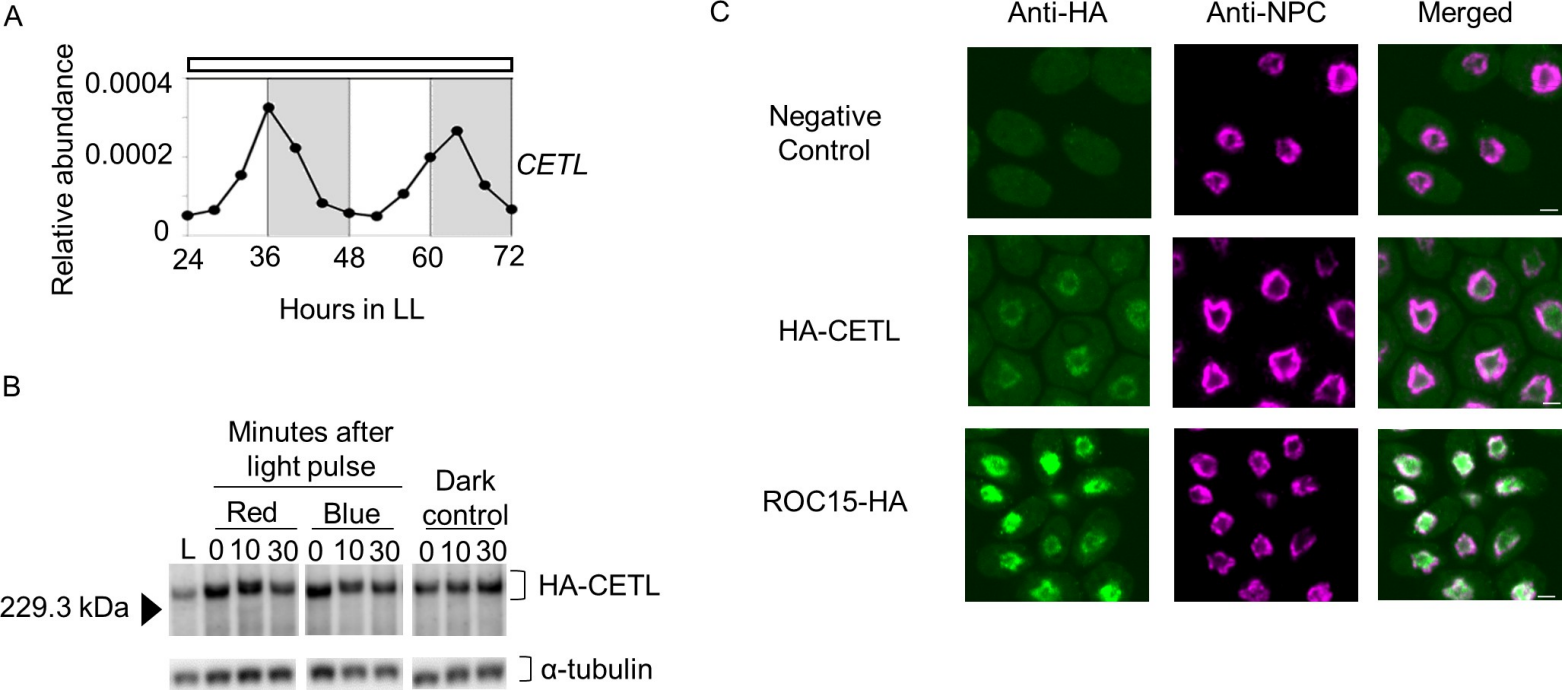
*CETL* gene expression pattern in LL conditions, CETL protein light response and subcellular localization. **A**. *CETL* mRNA rhythm in LL conditions. A free-running high-salt culture of the wild type (WT; ROC15-LUC mt^+^) was used. Cells were harvested every 4 hours between 24–72 hours in LL conditions and then subjected to RT-qPCR analysis. A representative trace of *CETL* mRNA levels (normalized by *RCK1* mRNA levels) is shown. The white bar above the graph represents the light conditions (i.e., LL conditions). The alternating white and grey background in the graph represents the subjective day and subjective night, respectively, that is expected from the light conditions prior to LL. **B**. CETL light response. High-salt cultures of complemented cells expressing HA-CETL were used. Cultures were maintained in LD conditions and exposed to 5 min pulses of red (10 μmol m^-2^ s^-1^) and blue (20 μmol m^-2^ s^-1^) light at ZT18. Western blot analysis was performed on whole protein extracts of cells harvested at ZT6 during the light phase (L), in the darkness before light pulse (0 min), and, 10 and 30 minutes after exposure to light pulses. Dark control was sampled at the same time points as the light pulsed samples. α-tubulin was used as loading control. **C**. CETL subcellular localization in comparison to ROC15 localization. Unsynchronized TAP cultures were maintained in darkness for 6 hours before cells were harvested in darkness and subjected to immunocytochemistry. The nuclear pore complex (NPC) was stained as a marker for the nuclear membrane. The ROC15-HA strain was used to observe ROC15 localization. The BR mt^+^ strain was used as a negative control. Results for a representative strain are shown. Scale bar: 2μm.

We analyzed whether *CETL* transcript level was rhythmic under constant light conditions (LL). The results of a reverse transcription-quantitative PCR (RT-qPCR) analysis revealed that the transcript-level was rhythmic, with a peak at approximately subjective dusk or early subjective night (**Fig 3A**). This result indicated that *CETL* gene expression was evening-phased due to circadian control.

To assess the CETL protein expression, the mutant (*cetl-1*) was transformed with a gene fragment encoding hemagglutinin (HA) tagged CETL (**S7A and S7B Fig**). A total of 462 transformants were screened for restoration of ROC15 response to 6 h dark/18 h red light cycles (**S7C Fig**). ROC15 response was restored to WT levels in six of the 462 transformants (**S7D Fig**). HA-CETL expression was then analyzed in these six transformants by western blot analysis. A band of size 237kDa corresponding to HA-CETL was detected in five of the six complements (**S7E Fig**). The ROC15 light response of these five complements was further confirmed by exposure to 5-min pulses of red and blue light. The ROC15 bioluminescence levels decreased acutely in response to red and blue light in all five complements (**S7F and S7G Fig**), indicating that the HA tagged CETL was functional with respect to ROC15 light induced degradation. CETL protein expression was then examined in one of these five complements by a western blot analysis. Bands corresponding to HA-CETL were detected in the light phase in a LD cycle and even 30 minutes after exposure to red and blue light pulses in the dark phase (**Fig 3B**). These results suggest that CETL does not show ROC15-like responses to either red or blue light. Immunocytochemical staining was also performed in one of the five complements by using anti-HA antibody. The HA-CETL signals were detected just inside the Nuclear Pore complex (NPC) signal, similar to where the ROC15-HA signals were detected (**Fig 3C**). These results suggest that CETL localizes in close proximity to ROC15.

### ROC15-LUC light response in the *cetl*-*1* mutant

**Fig 4:**
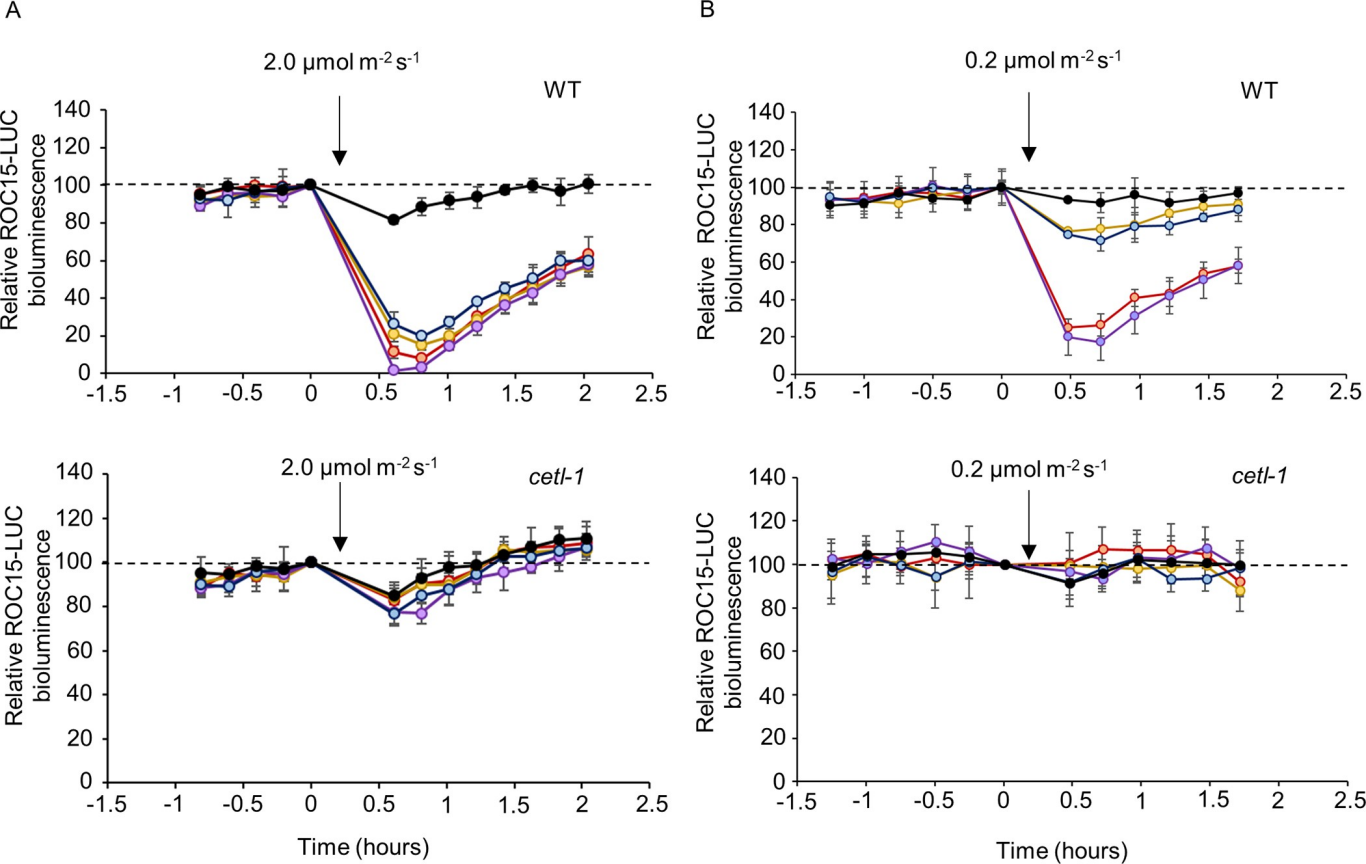
ROC15-LUC light response at different wavelengths in the *cetl-1* mutant. Spot cultures on high-salt agar were transferred to 96 well black plates, and bioluminescence was monitored as described in Materials and Methods. Akalumine was used as substrate. Cultures were maintained in darkness for at least 3 hours to allow for the build-up of ROC15-LUC, and then exposed to a 5-minute light pulse (violet, blue, yellow, or red) at two different intensities. **A** and **B**. Representative traces of ROC15-LUC bioluminescence in response to light pulses at intensities of 2 μmol m^-2^ s^-1^ and 0.2 μmol m^-2^ s^-1^, respectively, in WT (top panel) and *cetl-1* (bottom panel). Bioluminescence values have been calculated relative to those at the time point just before the light pulse. Mean ± SD of 4 biological replicates are shown. Arrows refer to the approximate time of the light pulse. Each color represents the wavelength of light to which the sample was exposed.

The *cetl-1* mutant showed an impaired response to red and blue light (**Fig 1**). To understand the wavelength specificity of this mutant, its ROC15 light response was further characterized by testing other wavelengths (i.e., violet and yellow). The mutant and WT were exposed to a light pulse of four different wavelengths at two different intensities. At an intensity of 2 μmol m^-2^ s^-1^, the WT showed an acute decrease in ROC15 bioluminescence levels at all wavelengths. In contrast, the mutant failed to show this acute decrease (**Fig 4A**). At a lower intensity of 0.2 μmol m^-2^ s^-1^, the WT cells showed an acute decrease in ROC15 bioluminescence levels in response to red and violet wavelengths, and a slight decrease in ROC15 bioluminescence levels in response to yellow and blue wavelengths (**Fig 4B**). This result was consistent with those of previous studies [27,28]. However, the mutant failed to show these decreases (**Fig 4B**). These results demonstrate that the mutant showed defective responses to violet and yellow wavelengths in addition to defective responses to blue and red wavelengths. Therefore, the ROC15 light response in the mutant was impaired across the visible range.

The effect of the *cetl-1* mutation on light-induced degradation of ROC15 was further observed in a *csl* mutant genetic background. The *cetl-1/csl* double mutant strain was obtained via a genetic cross. The double mutant was exposed to three different wavelengths (red, blue, and violet) at two different intensities. At both 2 μmol m^-2^ s^-1^ and 0.2 μmol m^-2^ s^-1^, the *cetl-1/csl* double mutant failed to show the ROC15-LUC bioluminescence decreases observed in the WT in response to all the wavelengths tested (**S8A and S8B Fig**). These results indicate that the blue light response in *csl* was lost due to the *cetl-1* mutation [28].

### Intracellular localization and light-induced phosphorylation of ROC15 in the *cetl-1* mutant

**Fig 5:**
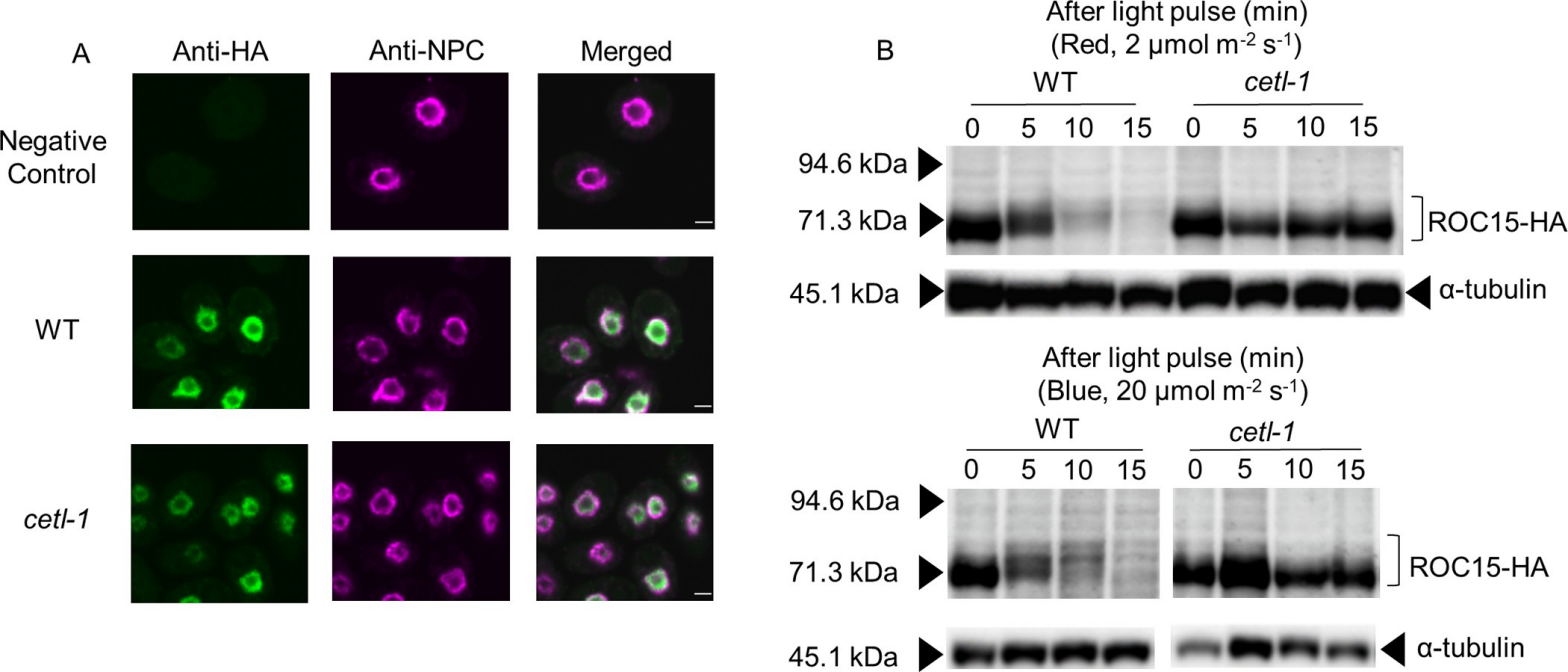
Subcellular localization and phosphorylation of the ROC15 protein in the *cetl-1* mutant. The complement (**S4 Fig**) was crossed with the ROC15-HA strain (wild type [WT]) to obtain progenies that inherited *ROC15-HA* (but not *ROC15-LUC*) along with the *cetl-1* mutation (*cetl-1*). Progenies were selected by antibiotic screening and genomic PCRs. **A**. ROC15 localization in the mutant. Unsynchronized TAP cultures were maintained in darkness for at least 6 hours to allow for accumulation of ROC15-HA. Cells were harvested in darkness and then subjected to immunocytochemistry. The NPC was counterstained as a marker for the nuclear membrane. The BR mt^+^ strain was used as a negative control. Scale bar: 2μm. **B.** Absence of light-induced ROC15 phosphorylation in the mutant. Cells in LD-entrained high-salt cultures were exposed to a 0.5-min light pulse of red (2 μmol m^-2^ s^-1^) and blue (20 μmol m^-2^ s^-1^) wavelengths at midnight (between ZT14 and ZT17). Western blot analysis was performed on whole protein extracts from cells sampled before the light pulse and at approximately 5, 10, and 15 minutes after the light pulse. The 0 min data corresponds to the sample just before the light pulse. WT in this experiment refers to a wild type sibling from the cross. α–tubulin was used as the loading control.

Previous studies have suggested that ROC15 localizes to the nucleus and undergoes light-induced phosphorylation [27]. To investigate these characteristics of ROC15 in the *cetl-1* mutant, the *ROC15-HA* genetic construct was introduced into the mutant genetic background via a genetic cross. To investigate ROC15 localization, we performed immunocytochemical staining with an anti-HA antibody. The results showed that ROC15-HA signals were detected immediately inside the NPC signals in both the WT and mutant cells (**Figs 5A and S9A**). This suggests that the *cetl-1* mutation did not affect the localization of the ROC15 protein. However, it was observed that the ROC15 expression between mutant cells was not as uniform as between WT cells (**S9A Fig**). This is probably due to the weak ability of this mutant to respond to synchronizing light/dark conditions (See below). This was followed by an analysis of ROC15 light-induced phosphorylation in response to red and blue lights. ROC15 phosphorylation has been reported to be visible as an electrophoretic mobility shift of ROC15-HA, as the shift was not observed when cell-extracts were subjected to phosphatase treatment [27]. This electrophoretic mobility shift was not observed in the mutant in response to red and blue light pulses (**Fig 5B**). The mobility shift was restored in the complement strains in both cases (**S9B and S9C Fig**), indicating that CETL was required for ROC15 to undergo light-induced phosphorylation.

### Circadian rhythm and resetting in the *cetl-1* mutant

**Fig 6:**
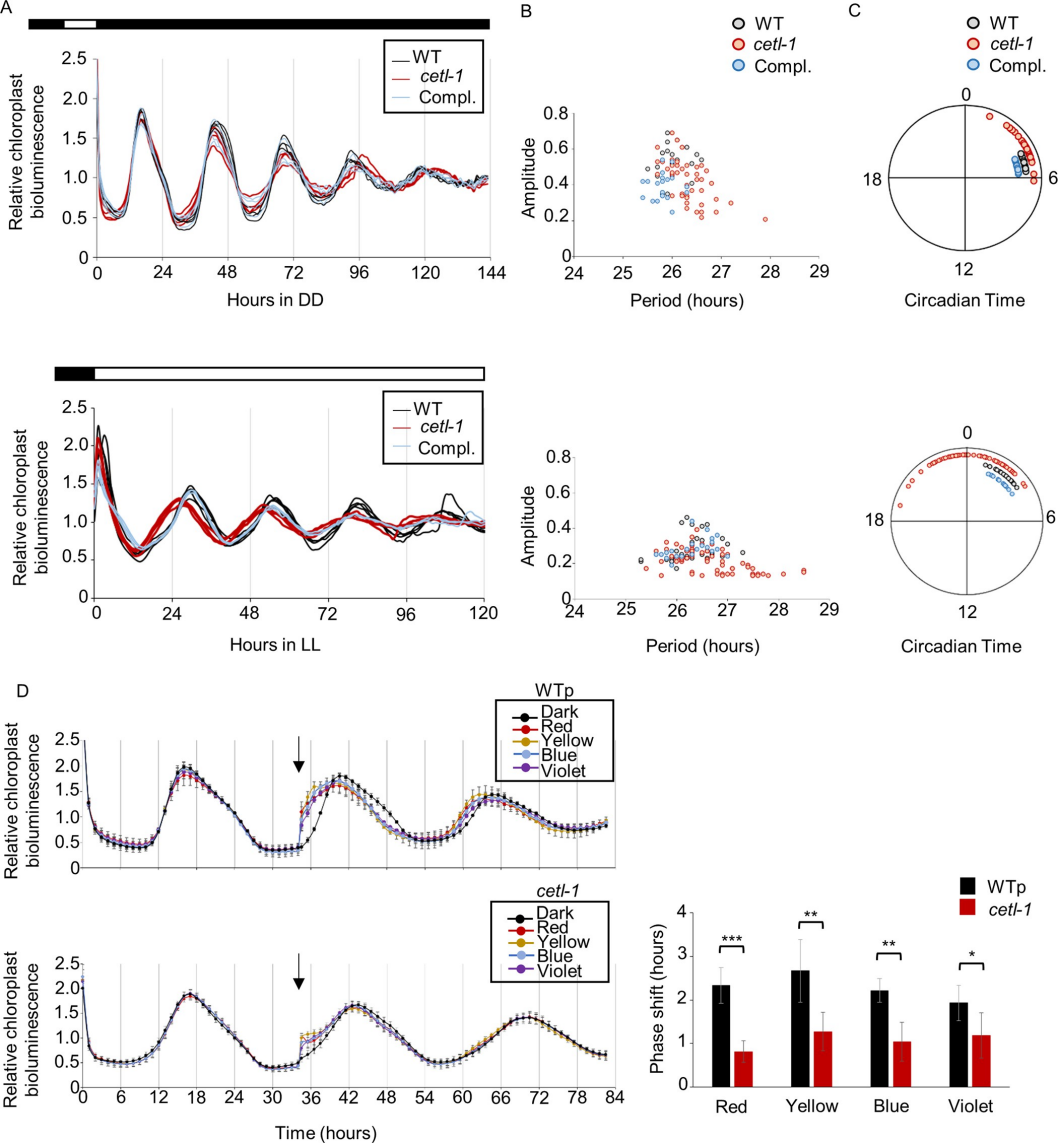
Circadian rhythm under DD and LL conditions and light-induced phase resetting. The mating type^-^ (mt^-^) strain of the *cetl-1* mutant (**S2 Fig**) and the complement (**S4 Fig**) were crossed with the CBR strain (mt^+^) (wild type [WT]) to obtain progenies that inherited the chloroplast luciferase reporter (but not the *ROC15-LUC*) along with *cetl-1* mutation (1–3), or along with both the *cetl-1* mutation and the *CETL* transgene (Compl.). These progenies were selected by antibiotic screening and genomic PCRs. Spot cultures of these progenies were synchronized by a 12-hour dark/12-hour light or 12-hour darkness before release into DD or LL conditions (2 μmol m^-2^ s^-1^), respectively. **A.** Representative traces of chloroplast bioluminescence rhythm in the *cetl-1* mutant under DD (top) and LL (bottom) conditions. Data of 3–5 biological replicates of the WT, a *cetl-1* progeny, and a complemented progeny from a single trial are shown. The light regime used is also depicted above the graphs. **B** and **C**. The period and amplitude (B) and phase diagram indicating the circadian phases (C) of the rhythms under DD (top) and LL (bottom) conditions. Data points correspond to the results of biological replicates of the WT and three *cetl-1* progenies across four trials, and of three complemented progenies from two trials (Refer to **S11 Fig** for the phase diagrams of individual *cetl-1* progeny from four different trials). The circadian times (CT) at the beginning of the DD and LL conditions were set to 12 and 0, respectively. **D**. Light-induced phase resetting under DD condition. Representative bioluminescence traces (left panel) and the amount of phase shift after the light pulse (right panel) are shown. A WT sibling (WTp) from the cross was used as WT for this experiment. Spot cultures were exposed to a 5-minute pulse of violet (5 μmol m^-2^ s^-1^), blue (38 μmol m^-2^ s^-1^), yellow (20 μmol m^-2^ s^-1^), and red (15 μmol m^-2^s ^-1^) light at approximately the 34^th^ hour after the onset of DD conditions. The amount of phase shift was calculated with respect to the dark control. Mean ± SD of 6–8 biological replicates are shown. *P*-values are based on Student’s *t*-tests: * *P*<0.05, ** *P*< 0.001, and *** *P*<0.0001.

To characterize the mutant, we observed its circadian rhythm in constant darkness (DD) and LL conditions. We introduced the chloroplast luciferase reporter gene (*tufA promoter-lucCP*; *lucCP* is a firefly luciferase gene that is codon-optimized for the *C*. *reinhardtii* chloroplast) [34] of the CBR strain [26] into a mutant genetic background via a genetic cross. A stable rhythm was detected in the *cetl-1* mutant under both DD and LL conditions (**Fig 6A**), with a slight tendency toward longer period lengths and lower amplitudes (**Fig 6B**). Similar results were observed in the *cetl-2* mutant under DD and LL conditions (**S10A and S10B Fig**). This result suggested that *CETL* did not play a major role in the oscillator of the *C*. *reinhardtii* circadian clock. However, the peak phase of *cetl-1* showed a wider distribution with a tendency to advance the phase, especially under LL conditions (**Fig 6C**). This is again close to what was observed in the *cetl-2* mutant under LL conditions (**S10C Fig**). As observed in the *cetl-1* mutant, the variation in phase was pronounced between independent trials of a single progeny, and also between progenies within a single trial, rather than between biological replicates of a single progeny within a single trial (**S11 and S12 Figs**). These unstable phase phenotypes might be indications of insufficient phase resetting in the mutants. Therefore, we investigated phase resetting using light pulses. The *cetl-1* mutant and WT were maintained in DD conditions and exposed to light pulses of four different wavelengths at late subjective night. The WT showed an advance in the phase in response to all four wavelengths, consistent with previous reports [28]. Although the *cetl-1* mutant also showed a slight phase advance, the advance was significantly reduced compared to that of the WT (**Fig 6D**). These results indicated that phase resetting was impaired in the *cetl-1* mutant.

### Phase distribution in the single colony protocol in the *cetl-1* mutant

**Fig 7:**
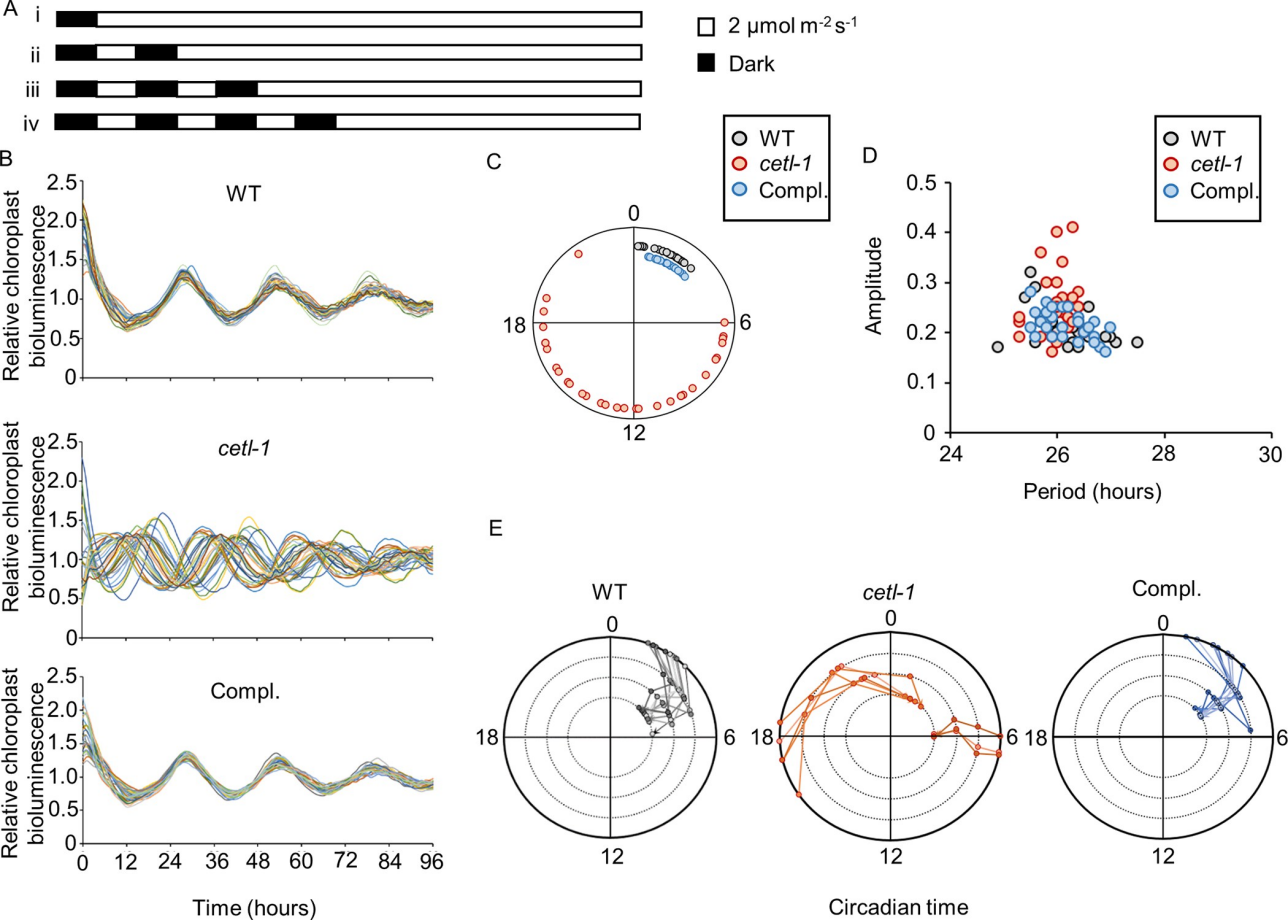
Phase distribution in LL in single colony protocol in *cetl-1*. The CBR (mt^+^) (wild type [WT]), mutant progeny (*cetl-1*), and complement progeny (Compl.) from the cross mentioned in **Fig 6** were used to observe the chloroplast bioluminescence rhythm. Spot cultures were prepared using the single colony protocol depicted in **S13 Fig**. **A**. Light regimes (**i**-**iv**) used for the experiment. Colonies were exposed to progressive LD (12 hours:12 hours) cycle before release into LL conditions (**ii-iv**). **B**. Chloroplast bioluminescence rhythms of the WT, *cetl-1*, and Compl. as observed after light schedule **i**. Cultures were maintained in 12 hours of darkness before release into LL conditions. Data of 28 biological replicates each, of WT and Compl., and 32 biological replicates of *cetl-1* from a single trial are shown. **C.** Phase diagram showing the circadian phases of *cetl-1*, WT and Compl. rhythms shown in B. **D** Plot of period and amplitude of the bioluminescence rhythms shown in B. **E.** Peak phases of the quadruplicate spots of biological replicates after each of the four light schedules. The results after light schedules **i**-**iv** are plotted from the outermost ring to the innermost ring, respectively. Quadruplicate spots from the same independent culture are connected by a line. Data are shown only for cases where the rhythms were detected for all four spots in an independent culture, and correspond to results of a single trial.

To further examine the phase resetting in the mutant, we also analyzed the chloroplast bioluminescence rhythm of cultures that had been prepared in a manner different to that of our standard protocol. The biological replicates for the current experiment originated from independent colonies (single colony protocol), as opposed to our standard protocol, where the biological replicates were obtained from a single patch on storage plates (**S13 Fig**). The quadruplicate spots from an independent culture of the single colony protocol were exposed to either the same light schedule as in **Fig 6A** (**Fig 7Ai**) or to additional progressive LD cycles (**Fig 7Aii, 7Aiii and 7Aiv**) before monitoring in LL conditions. Surprisingly, after light schedule i, the peak phases appeared to vary largely between the biological replicates of *cetl-1* (**Fig 7B**). When visualized as a phase diagram, the wide distribution of peak phases was evident (**Fig 7C**). We also noted that the distribution of peak phases of *cetl-1* varied with trials, and sometimes appeared more synchronized (**S14A and S14B Fig**). However, we again observed that the period and amplitude of the rhythm of the mutant were close to those of the WT in this protocol (**Figs 7D and S14C).** After each additional light/dark cycle (**Fig 7Aii, 7Aiii, and 7Aiv**), the peak phases of the rhythms in the mutant cultures were detected much closer to those of the WT (**Fig 7E**). Collectively, these results suggested that the mutant was severely affected in its ability to reset the clock, but that the ability is not completely lost.

### Light responses of clock gene mRNAs in the *cetl-1* mutant

**Fig 8:**
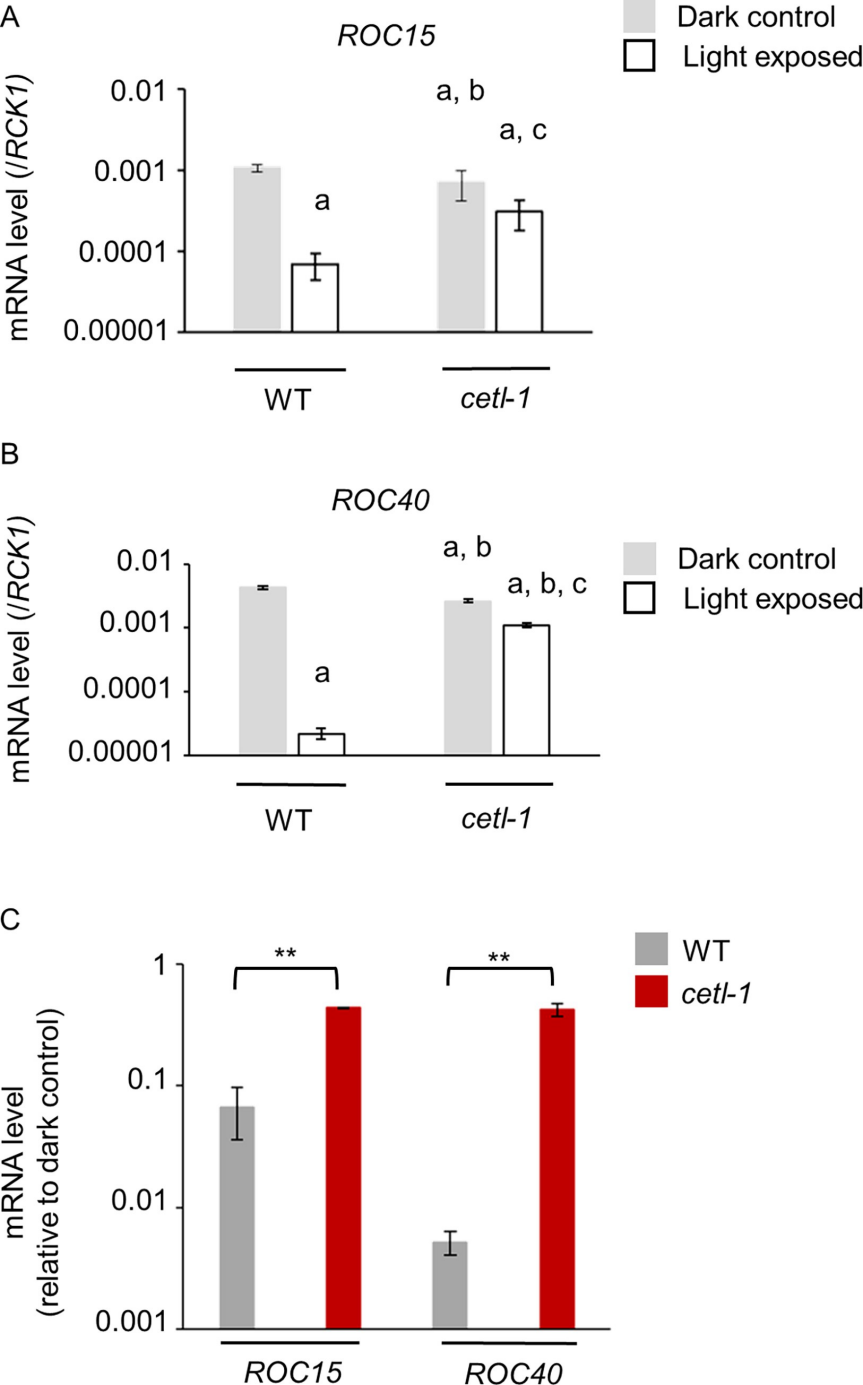
Light response of clock gene mRNAs in the *cetl-1* mutant. LD-entrained high-salt cultures were exposed to 30 μmol m^-2^ s^-1^ of white light at late night (ZT22, 2 hours before dawn). Cells were harvested from the light-exposed culture and the corresponding dark control after 1 hour. **A** and **B**. The mRNA levels of *ROC15* (A) and *ROC40* (B) in the wild type (WT; ROC15-LUC strain) and *cetl-1*. The mRNA levels have been calculated relative to the mRNA levels of *RCK1*. A two-way ANOVA indicated a significant interaction between genotype and light conditions for *ROC15* (genotype: *F*_1,8_ = 0.46, *P* = 0.52, light conditions: *F*_1,8_ = 54.61, *P*<0.0001, interaction: *F*_1,8_ = 10.24, *P*< 0.05) and for *ROC40* (genotype: *F*_1,8_ = 12.46, *P* = 0.0077, light conditions: *F*_1,8_ = 1199.57, *P*<0.0001, interaction: *F*_1,8_ = 272.17, *P*< 0.0001). Tukey’s post hoc test was performed on the samples which were subdivided into four groups: WT dark control, WT light exposed, *cetl-1* dark control, and *cetl-1* light exposed. **a** (*P<0*.*05*) for comparisons with WT dark control, **b** (*P<0*.*05*) for comparisons with WT light exposed sample, **c** (*P<0*.*05*) for comparisons with *cetl-1* dark control. **C.** Extent of downregulation in WT and *cetl-1*. The mRNA abundance (i.e., mRNA levels relative to *RCK1* mRNA levels) of the light-exposed samples are shown relative to that of their respective dark control for easy comparison. *P*-values are based on Student’s *t*- tests. ** *P<* 0.01. Mean ± SD of three biological replicates is shown (A-C). Note that these strains possess two copies of *ROC15*: an endogenous copy and *ROC15-LUC* transgene. The *ROC15* mRNA levels detected in this experiment are therefore a sum of the mRNA levels of the endogenous copy and the mRNA levels of the *ROC15-LUC* transgene.

Previous studies have demonstrated that the mRNA of some clock genes, such as those of *ROC15* and *ROC40*, are downregulated in response to light [20,27,28,35]. Here, we investigated this phenomenon in the *cetl-1* mutant. RT-qPCR was performed on samples from WT and *cetl-1* cultures exposed to light for 1 h in the late night. In WT, the mRNA levels of *ROC15* and *ROC40* were much lower in the light-exposed samples than in the dark controls (**Fig 8A and 8B**). The mRNA levels of the *ROC15* and *ROC40* genes also appeared to be downregulated in the light-exposed sample of *cetl-1* (**Fig 8A and 8B**). However, the decline in mRNA levels was much lesser in the *cetl-1* mutant than in the WT (**Fig 8C**). The weak responses of both the mRNAs were restored to WT levels in the complement strain (**S15 Fig**). These results suggested that *CETL* played a role in the light responses of *ROC15* and *ROC40* mRNAs.

### Circadian rhythm of clock genes in the *cetl-1* mutant

The mRNAs of certain clock genes have been shown to be rhythmic under LL conditions [26]. We examined whether the *cetl-1* mutation had any effect on the expression rhythm of clock genes (*ROC15*, *ROC40*, *ROC66*, and *ROC75)* under LL conditions. The RT-qPCR analysis (**S16 Fig**) revealed that all four clock genes tested in the mutant had rhythmic gene expression patterns. However, some differences were observed in certain cases. The level of *ROC15* mRNA appeared to be slightly lower in *cetl-1* mutants, whereas the *ROC40* mRNA levels appeared to be slightly higher, especially during the subjective day (**S16A and S16B Fig**). The phase of expression of *ROC40* and *ROC66* appeared to be slightly delayed in *cetl-1* mutants (**S16B and S16D Fig**). These results suggested that *CETL* might not have a great influence on the transcriptional oscillator.

## Discussion

### Light signaling pathway for ROC15 degradation

Three possible models for light induced ROC15 degradation are depicted in S17 Fig. A previous study indicated that a wide range of wavelengths were capable of causing ROC15 degradation [27]. The CSL protein, which mainly localizes in the cytoplasm, was indicated to be specific to the red/violet light signalling pathway upstream of ROC15 phosphorylation [28] (**S17 Fig**). In contrast, the clock protein ROC114, which localizes in the nucleus, was shown to be common to the different light signalling pathways downstream of ROC15 phosphorylation [27] (**S17 Fig**). In this study, we revealed that CETL, another protein which appears to localize in the nucleus (**Fig 3C**), is also common to the different light signalling pathways (**Fig 4**). In addition, impairment of the light induced phosphorylation in the *cetl-1* mutant (**Figs 5B, S9B and S9C**) indicates that unlike ROC114, CETL plays a role upstream of ROC15 phosphorylation (**S17 Fig**). Furthermore, since the blue light response that remained in the *csl* mutant was completely lost in the *csl*/*cetl* double mutant (**S8 Fig**), it seems likely that these light signals are integrated downstream of CSL. However, it is still unclear whether the different light signalling pathways are integrated by CETL (**S17A Fig**) or another unidentified component X upstream of CETL (**S17B Fig**). In addition, there also exists a possibility where CETL is essential for the expression of other unknown components (X and Y) that are upstream of ROC15 phosphorylation (**S17C Fig**).

### Mechanisms contributing to light resetting in *Chlamydomonas*

The light resetting ability of the circadian clock was not completely lost in the *cetl-1* mutant (**Figs 6 and 7**) despite a defect in the light-induced degradation of ROC15 in response to a wide range of wavelengths (**Fig 4**). This pattern points to the possibility of other mechanisms regulating the light-induced resetting of the *Chlamydomonas* circadian clock. One such mechanism is the light response of the mRNAs of clock genes, such as *C3*, *ROC15*, *ROC40*, and *ROC75* [20, 27, 28, 35]. The light-induced downregulation of *ROC15* and *ROC40* mRNAs was diminished in the *cetl-1* mutant (**Fig 8**), which is in contrast to the trend observed in the *csl* mutant [28]. In the *csl* mutant, the red light-induced degradation of the ROC15 protein was impaired, whereas the light responses of the *ROC15* and *ROC40* mRNAs were similar to those of the WT under strong red light conditions (200 μmol m^-2^ s^-1^) [28]. We cannot exclude the possibility that the *csl* mutant may show a defect in the mRNA response at the weaker light intensities used in the current study (30 μmol m^-2^ s^-1^). However, the *cetl-1* mutant is the first in which we have confirmed a defect in both light-induced ROC15 degradation and the light responses of clock gene mRNAs. It is intriguing that the *cetl-1* mutant did not completely lose its resetting ability (**Figs 6** and **7**) even with both the potential resetting mechanisms affected. This suggests the existence of yet another contributing factor which is currently unknown.

### Wide distribution of phases in the *cetl-1* mutant

Interestingly, mutants obtained using the single colony protocol exhibited a wide distribution of phases in some cases (**Fig 7**). A recent study has demonstrated that a liquid culture of *Chlamydomonas* comprises cells in various circadian phases [36]. Another study that analyzed co-expression networks in *Chlamydomonas* further estimated that 21–96% of cells from a culture grown in LL conditions were synchronized [37]. It has also been shown that in mammalian cells, the circadian rhythm of mother cells is resumed in daughter cells after cell division [38]. In the single colony protocol, the colonies are therefore assumed to have a distribution of phases, with each colony possessing the phase of one of the cells in the liquid culture. This phase distribution should be reset by either the 12-hour dark period or the light/dark cycles (for the WT cells). We speculate that the wide distribution of phases observed in the mutant (**Figs 7C and S14B**) is a reflection of the phase distribution of the liquid culture, possibly due to the reduced ability of the mutant to be reset by the light/dark regime before release into LL conditions. This is contrary to the narrower distribution of phases observed within a single trial of the standard protocol (**Fig 6**). This difference may also suggest that cells in patch cultures had a more uniform phase than those in liquid culture. Furthermore, the variation in the distribution of phases between trials in the mutant (**Figs 7C and S14B**) may be attributed to the variation in the synchronization of cells in the liquid culture [37].

### Comparison of CETL and ELF3

#### i) Similarities between CETL and ELF3

The notion that CETL is a potential homolog of ELF3 (**Figs 2B, 2C, and S5**) is extremely curious, as no obvious homolog of ELF3 has been found in *C*. *reinhardtii* to date [39,40]. Comparison of the characteristics of the two reveals three main points of similarity. The first involves their expression patterns: similar to the *ELF3* expression pattern [41], *CETL* gene expression was also shown to be under circadian control, with an expression peak during subjective dusk (**Fig 3A**). The second similarity is with respect to their involvement in the input pathway of the circadian clock. Our results suggest that *CETL* integrates red and blue light inputs (in addition to violet and yellow lights) into the circadian clock (**Figs 1 and 4**), which is similar to one of the roles of *ELF3* i.e., integrating red and blue light into the *Arabidopsis* circadian clock [8]. The third similarity is their close involvement with circadian-clock-related GARP proteins that are expressed at night (i.e., LUX/BOA in *A*. *thaliana* and ROC15 in *C*. *reinhardtii*). ELF3 has been shown to be directly associated with LUX and BOA in *A*. *thaliana* EC [42]. Although there is no evidence for a direct association between CETL and ROC15, the similar localization patterns of CETL and ROC15 (**Fig 3C**) and the involvement of CETL in the ROC15 light response (**Figs 4, 5B, S9B and S9C**) suggests a close relationship between the two. Importantly, CETL was found by random screening using the GARP protein, ROC15, as an indicator. Based on these similarities, we suggest the possibility of functional conservation of the CETL and ELF3 proteins during evolution.

#### ii) Differences between CETL and ELF3

*CETL* appears to differ from *ELF3* in some aspects, with respect to its role in the clock. The first difference is regarding their effect on the input to the clock. Since the ROC15 light response was impaired in the *cetl-1* mutant (**Figs 4 and 5B**)—which is likely a loss-of-function mutant—*CETL* appears to facilitate the light input to the clock. However, it has been suggested that *ELF3* antagonizes the light input to the clock [8,9]. The second difference is the rhythmicity under LL and DD conditions. The *cetl-1* and the *cetl-2* mutants were found to be rhythmic under both LL (**Figs 6A, 7B, S10A, S12 and S14A**) and DD conditions (**Figs 6A and S10A**), suggesting that *CETL* does not influence the oscillator to a great extent. In contrast, the *elf3* mutant was found to be arrhythmic under LL conditions [8] and conditionally arrhythmic under DD conditions, depending on the reporter used [8,43,44]. These results suggest that CETL has a weaker influence on the oscillator than ELF3. The third difference is with respect to the regulation of clock-related Myb transcription factor genes (i.e., *LHY*/*CCA1* in *A*. *thaliana* and *ROC40* in *C*. *reinhardtii*). The *elf3* mutant shows lower *LHY/CCA1* levels than the WT [45]. ELF3 is also known to repress the gene expression of the *PSEUDO RESPONSE REGULATOR* 9 (PRR9) [46], which is also one of the transcriptional repressors of *LHY/CCA1* [47,48]. It has therefore been proposed that ELF3 indirectly activates *LHY/CCA1* by repressing the gene expression of the *LHY/CCA1* repressors such as PRR9 [32,46]. In contrast, the *ROC40* expression was not greatly affected or was slightly upregulated in the *cetl-1* mutant (**S16B Fig**). In the mutant, the slightly higher level of expression of *ROC40* may be associated with the weak light-induced downregulation of *ROC40* mRNAs (**Figs 8 and S16B**), or possibly an indirect effect of the impaired light-induced degradation of ROC15 (**Figs 1 and 4**). These differences may be due to the potential differences between the circadian clock systems of *C*. *reinhardtii* and *A*. *thaliana*.

### Conclusion

The possibility of the existence of an ELF3 homolog opens up many avenues of exploration that would enable further elucidation of the *C*. *reinhardtii* circadian clock. One such avenue is the existence of an EC in *C*. *reinhardtii*, since *C*. *reinhardtii* also has a potential homolog of the other EC component, ELF4 [39,40]. ELF3 has been shown to have thermo-sensing abilities due to a prion-like domain [33] and is shown to be involved in temperature entrainment of the *A*. *thaliana* clock [44]. Therefore, another possibility is that *CETL* plays a role in the integration of temperature signals to the clock. In *C*. *reinhardtii*, the C1 and C3 subunits of the clock-related protein complex, Chlamy1, can integrate temperature information into the circadian clock [49]. This may provide a gateway to study the involvement of CETL in temperature integration into the circadian clock. In conclusion, this study has revealed many pathways that could be explored to improve our understanding of temporal organization in *Chlamydomonas* and the evolution of circadian clocks in Viridiplantae.

## Materials and methods

### Strains and media

We used the ROC15-LUC mating type ^+^ (mt^+^) and mating type ^-^ (mt^-^) [27], CBR (mt^+^) [26], BR (mt^+^) [27], *b19* (*cetl-2*) (mt^+^) [28], *csl* (mt^+^) [28], and ROC15-HA (mt^+^) [27] strains. BR is a reporter-less strain used to measure background noise during bioluminescence monitoring experiments. All strains were maintained on agar plates containing with Tris-acetate-phosphate (TAP) [50] medium and stored in constant white light conditions (10–20 μmol m^-2^ s^-1^) at 24°C (storage culture). The storage cultures were re-plated every 1–1.5 months. A high-salt (HS) medium [51] was also used in this study. All *C*. *reinhardtii* strains used in this study were derived from the CBR34 strain [26], which was obtained from a genetic cross between a CC2137-based reporter strain (*tufA* promoter-*lucCP*) [34] and the SAG11-32a WT strain.

### Light sources

The light sources that were used in this study are detailed henceforth. LED tubes (red LED: LT20RS, 636 nm [full width at half maximum of 21 nm]; blue LED: LT20BS, 458 nm [21 nm], Beamtec, Saitama, Japan) were used for mutant screening (**Figs 1**, **S1, S2, S4B and S7C**). LED panels (red LED: ISL-150×150-RR, 660 nm [24 nm], yellow LED: ISL-150×150-YY, blue LED: ISL-150×150-BB, 470 nm [27 nm], violet LED: ISL-150×150-VV, 405 nm [14 nm], CCS, Kyoto, Japan) were used for light pulse and wavelength specificity experiments (**Figs 1B, 1C, 3B, 4, 5B, 6D, S1B, S1C, S4E, S4F, S7F, S7G, S8, S9B and S9C**). The wavelength of the yellow LED was measured using a multi-channel spectrometer (MC2100, Otsuka Electronics, Osaka, Japan). The peak was detected at 570nm [11nm]. White LED panels (MLP-LSK2478DA5, Musashi Electric, Saitama, Japan) and white LED tubes (LT-40KY-III, Beamtec, Saitama, Japan) were used as background light during rhythm assays (**Figs 6A, 7, S10, S12 and S14**). Light intensity was measured with a light meter (LI-250 equipped with LI-190, LI-COR, NE, USA; LA-105, NK systems, Osaka, Japan). Blue LEDs were used as safety lights at lower intensities that were undetectable by the light meters. Fluorescent tubes (FL20SSW/18-B, Hitachi, Tokyo, Japan) were used for culturing and mRNA analysis (**Figs 3A, 8, S15 and S16**).

### Culture conditions

The three main types of cultures used in the study are detailed below.

1. Unsynchronized TAP cultures: Cells from the storage cultures were inoculated into TAP medium and maintained at 24°C in LL (30–40 μmol m^-2^ s^-1^) for 3–4 days (starter culture). Cells from starter cultures were then inoculated into fresh TAP medium to achieve a final concentration of 1 × 10^5^ cells/mL and maintained under the same conditions as the starter culture for 2 days.
2. LD-entrained and free-running HS cultures were prepared as previously described [35]. Briefly, starter cultures were inoculated into fresh HS medium at a concentration of 2 × 10^5^ cells/mL and maintained at 24°C in LL (30–40 μmol m^-2^ s^-1^) for 3 days. The temperature was then reduced to 17°C, and lights turned off for 12 hours to synchronize the circadian clock. After the synchronization period, the LD-entrained cultures were maintained in a 12-hour light (10 μmol m^-2^ s^-1^)/12-hour dark cycle, and free-running cultures were kept in LL conditions (10 μmol m^-2^ s^-1^).
3. Spot cultures for bioluminescence monitoring: Cells were cultured for 3 days in 96 well plates (Nunc MicroWell, Thermo Fisher Scientific, MA, USA) in 100 μL of TAP medium maintained at 24°C in LL conditions (30–40 μmol m^-2^ s^-1^). Following this, 5 μL of culture was spotted onto 1.5% HS agar plates (Agar BA-70, Ina Food Industry, Nagano, Japan was used for rhythm assays and Agar against dryness (LG), Kanto Chemical, Tokyo, Japan was used for light response assays). The spots were allowed to grow for 4 days at 24°C in LL conditions (30–40 μmol m^-2^ s^-1^).

### Genetic crosses and TAIL-PCR

Genetic crosses and TAIL-PCR were performed as described previously [26].

### Plasmid construction

To obtain the pLaadA/CETL plasmid, the blunt-ended 11.9 kb SacI/BamHI fragment from a bacterial artificial chromosome containing the *CETL* gene—obtained from the C9 strain—was sub-cloned into the EcoRV-digested pLaadA plasmid [28]. This fragment also contained the 264 nucleotide insertion. To obtain pLaadA/HA-CETL plasmid, an EcoRV restriction site was introduced just after the second predicted start codon of CETL in the pLaadA/CETL plasmid by PCR-based mutagenesis. A codon adapted HA sequence (**S7B Fig**) was inserted at the EcoRV site by using In-Fusion technology (TakaRa Bio, Shiga, Japan).

### Bioluminescence monitoring

Cultures were transferred into 96 well white plates (Nunc F96 MicroWell, Thermo Fisher Scientific), 96 well black plates (STREIFEN-PLATTE [762076], Greiner Bio-One, Kremsmünster, Austria), or 24 well black plates (Krystal Microplates, Porvair Sciences, Norfolk, UK). In cases where spot cultures needed to be transferred, the spots on the agar were cut along with the agar and transferred into wells using a glass tube (inner diameter: 6 mm). Luciferase substrates were added to each well at a final concentration of 100 μM (liquid cultures) or approximately 200 μM (spot cultures). D-luciferin (Biosynth, Staad, Switzerland) was used unless specifically mentioned, and the luciferin analog Akalumine-HCL (FUJIFILM Wako Pure Chemical Corporation, Osaka, Japan) was used in some experiments [52]. Bioluminescence was monitored at 24°C using a custom-made automatic bioluminescence apparatus [53,54] and commercially available instruments (CL24A-LIC and CL96S-4, Churitsu Electronic Corporation, Nagoya, Japan). In each cycle, bioluminescence was measured after at least 3.5 min of dark exposure to ensure a decrease in delayed light emission of chlorophyll. The results of bioluminescence monitoring were analyzed using the Rhythm analysis program (RAP [53,54]) and Kaiseki NINJA (Churitsu Electronic Corporation, Nagoya, Japan). Bioluminescence rhythm data were de-trended by dividing by the 24-hour moving average. The Cosinor-rhythmometry method was used to analyze bioluminescence rhythm data over 3–4 days. Data for which the curve fitting was obviously inaccurate or had a high error index (>0.055) were not considered for further analysis. For the analysis of the ROC15-LUC reporter, luminescence detected in empty wells or in cultures of the reporter-less BR strain was considered background noise and subtracted unless specifically mentioned.

### Transformation

Transformation of the *C*. *reinhardtii* nuclear genome was achieved by electroporation, as described previously [27,55]. For mutagenesis, ROC15-LUC mt^+^ was used as a host strain for transformation with the *aph7”* fragment. The *aph7”* fragment for mutagenesis was obtained by digesting the plasmid pHyg3 [29] with the HindIII restriction endonuclease. The 1.7 kb fragment was purified after agarose gel electrophoresis, and 30 ng of DNA (/~3 × 10^7^ cells) was used for the transformation. Transformants were selected on TAP agar plates with a final hygromycin concentration of 30 μg/mL. For complementation, the pLaadA/CETL plasmid was digested with the PacI restriction endonuclease. The resulting 14.3 kb fragment was purified after agarose gel electrophoresis. DNA fragments (300 ng /~3 × 10^7^ cells) were used for transformation, and transformants were selected on TAP agar plates with a final spectinomycin concentration of 50 μg/mL.

### Mutant/Complement screening

The transformant colonies were inoculated into 100 μL of TAP medium and maintained in 96 well plates (Nunc MicroWell, Thermo Fisher Scientific) for 3 days at 24°C in LL conditions (30–40 μmol m^-2^ s^-1^). Following this, 5 μL of the culture was transferred into 100 μL of fresh TAP medium containing D-luciferin (final concentration, 100 μM) in 96 well white plates (Nunc F96 MicroWell, Thermo Fisher Scientific). The plates were maintained at 24°C in LL conditions (30–40 μmol m^-2^ s^-1^) for 1 day before bioluminescence monitoring. Bioluminescence was monitored using a custom-made automatic bioluminescence apparatus [53, 54].

### Protein analysis

Western blot analysis was performed as previously described [27]. Polyacrylamide gel (12.5%) (e-PAGEL, ATTO Corporation, Tokyo, Japan) was used for the observation of light- induced phosphorylation of ROC15 (**Figs 5B, S9B and S9C**) and 7.5% polyacrylamide gel (e-PAGEL, ATTO Corporation) was used for the detection of HA-CETL (**Figs 3B and S7E**). The transfer step was performed using a Qblot kit, EZ blot kit and EZFastBlot *HMW* kit (ATTO Corporation)). BLOCK ACE Powder (KAC, Kyoto, Japan) was used to block the membranes. Rat monoclonal anti-HA antibody was used as the primary antibody (1:10000, clone 3F10, Roche, Basel, Switzerland). Horseradish peroxidase-conjugated goat anti-rat IgG was used as a secondary antibody (1:25000, Merck KGaA, Darmstadt, Germany).

Immunocytochemistry was performed as described previously [27]. Rat monoclonal anti-HA (1:1000) and mouse monoclonal anti-NPC (1:1000, clone MAb414, Labcorp, NC, USA) were used as primary antibodies. Alexa Fluor 488-conjugated goat anti-rat IgG (Thermo Fisher Scientific) and Alexa Fluor 647-conjugated goat anti-mouse IgG (Thermo Fisher Scientific) were used as secondary antibodies. Fluorescence was observed using a laser scanning confocal fluorescence microscope (FV10i-DOC; Olympus, Tokyo, Japan).

### RT-qPCR

RT-qPCR was performed as described previously [28]. The primers used for quantification of *ROC15*, *ROC40*, *ROC66*, *ROC75*, and *RCK1* transcripts are the same as previously described [35]. Primers used for quantification of *CETL* transcript are listed in S1 Table.

## Supporting information

S1 Fig ROC15 light response of the M2 mutant. **A**. ROC15-LUC bioluminescence pattern of the M2 mutant, observed under diurnal conditions. The red, blue, and black bars above the graph represent red light (8 μmol m^-2^ s^-1^), blue light (20 μmol m^-2^ s^-1^), and dark conditions, respectively. Cells were prepared as described in Materials and Methods (mutant/complement screening). The results of the initial screening are shown. The trace of a transformant (T), exhibiting a WT response, is shown for comparison. The background was not subtracted. **B** and **C.** ROC15 bioluminescence response to light pulses in the M2 mutant. Unsynchronized TAP cultures were transferred into 24 well black plates, and their bioluminescence was monitored. Cells were kept in darkness for at least 4–6 hours to allow for the accumulation of ROC15-LUC, and then exposed to a 5-min pulse of red (**B**, 10 μmol m^-2^ s^-1^) and blue (**C**, 5 μmol m^-2^ s^-1^) light. Bioluminescence has been calculated relative to the time point just before the light pulse (Time 0). Mean ± SD of 4 biological replicates are shown. Arrows indicate approximate time of light pulses.
(TIF)

S2 Fig Genetic linkage analysis of M1 and M2. M1 and M2 mutants were backcrossed with the parental ROC15-LUC reporter strain. The progeny were subjected to hygromycin resistance screening and ROC15 bioluminescence monitoring in response to red light (8 μmol m^-2^ s^-1^). **A** and **C**. Bioluminescence trace of the progeny in response to light, along with the approximate light schedule used. Cells were prepared as described in Materials and Methods (mutant/complement screening). All values were calculated relative to that at the time point (0-hour) just before the start of light phase. The background was not subtracted. Results of 94–96 individual progeny have been plotted. **B** and **D**. Distribution of the numbers of progeny showing hygromycin sensitivity, and their relative bioluminescence levels after exposure to light (corresponding to hour 2 in **A** and **C**).
(TIF)

S3 Fig Confirmation of the *aph7”* insertion. Primer binding sites and PCR confirmation of the insertion of the hygromycin resistance gene (*aph7”*). **A.** Schematic representation of the Cre07.g357500 gene and its transcript. Blue triangles around the insertion loci indicate the binding sites of the primers used for PCR confirmation in the genomic DNA. The red triangles indicate the binding sites of the primers used for PCR confirmation in the cDNA. **B**. PCR was performed on the wild type (WT) and mutant genomic DNA with primers targeted around the insertion loci (depicted in A). The amplified products were visualized on an agarose gel. **C**. RT-PCR was performed on the Cre07.g357500 transcript in the WT and mutant with primers targeted to the loci depicted in A. The amplified products were visualized on an agarose gel. The primers used are listed in S1 Table.
(TIF)

S4 Fig Complementation by the Cre07.g357500 gene. **A.** Schematic representation of the gene fragment used for complementation. WT genomic DNA fragment of Cre07.g357500 gene ligated to the spectinomycin resistance cassette (*aadA)* was used for complementation. **B.** A representative ROC15-LUC bioluminescence pattern of one of the six complements. Culture preparation and light conditions were the same as in **Fig 1A**. Mean ± SD of 8 biological replicates of the complement (Compl.) have been plotted. The bioluminescence trace of the WT and M1 from **Fig 1A** have been plotted for comparison. **C** and **D**. Histograms of the distribution of transformants with respect to the relative bioluminescence levels after exposure to red light (8 μmol m^-2^ s^-1^). The bioluminescence values at the first time point after the start of red light exposure were taken relative to the values just before the start of red light exposure. Transformants with only *aadA* are shown as a negative control. **E** and **F.** Representative trace of the light-pulse response of the six isolated complements. Unsynchronized TAP cultures were transferred into 24 well black plates and their bioluminescence was monitored as described in the Materials and Methods. Cells were kept in darkness for at least 3 hours before being exposed to a 5-min pulse of red light (**E**, 10 μmol m^-2^ s^-1^) and blue light (**F**, 10 μmol m^-2^ s^-1^). Bioluminescence values have been calculated relative to the time point just before the light pulse (Time 0). Background was not subtracted. Arrows indicate the approximate time of the light pulse.
(TIF)

S5 Fig Protein sequence alignment. A. Schematic representation of all the protein sequences used in the alignment and the two regions of similarity (Region 1 and Region 2). B. Alignment of the sequences at Region 2. Amino acids have been colored according to their properties and conservation (Clustal X color scheme). Percentage of conservation at each site is indicated by colored bars above the alignment. Sequences were aligned in the same manner as in **Fig 2**. Amino acid sequences used for the alignment are the same as those used in **Fig 2**.
(TIF)

S6 Fig Protein sequence alignment with only angiosperm ELF3. Protein encoded by Cre07. g357500 was aligned with *A*. *thaliana* ELF3 and its homologs from *O*. *sativa* (ELF3-1), *S*. *lycopersicum* and *Z*. *mays* using MAFFT (MAFFT 7.471, SnapGene) and MUSCLE (MUSCLE 3.8.1551, SnapGene) multiple sequence alignment algorithms. Block I, II, III, and IV are highlighted according to the regions recognized as Blocks in *A*. *thaliana* ELF3 [11]. Conserved and similar amino acids are represented in the Clustal format. Region 1 and Region 2 are underlined in red and orange respectively, in both alignments. Details of the amino acid sequences can be found in **S2** Table.
(PDF)

S7 Fig Complementation by *HA-CETL* gene. **A.** Schematic representation of the gene fragment used for complementation. An HA tag, codon adapted for the *C*. *reinhardtii* nuclear genome was incorporated after the second predicted start codon of the *CETL* gene that was ligated to *aadA*. **B.** The codon adapted sequences of the HA tag and a flexible GS linker. **C**. Representative trace of the ROC15-LUC bioluminescence pattern of one of six complements. Culture preparations were the same as in **Fig 1A**. Cultures were subjected to 6 h dark /18 h red light (2 μmol m^-2^ s^-1^) cycle. The representative trace of one of the six complements is shown in comparison to the traces of the WT and *cetl-1* (n = 1). **D.** Histogram showing the distribution of the transformants with respect to the ROC15 bioluminescence levels at the first time point after red light exposure. Bioluminescence levels were calculated relative to the value just before light exposure. **E.** Western blot analysis of the complemented strains. Unsynchronized TAP cultures were maintained in darkness for 6 hours. Western blot was performed on whole protein extracts from cells harvested in darkness. The WT BR mt^+^ strain was used as the negative control. **F** and **G** Light pulse response of five of the six complements. Spot cultures were prepared as described in **Fig 4** and their bioluminescence was monitored as described in materials and methods. Cells were in darkness for at least 6 hours before being exposed to 5-min pulses of red (**F**, 5 μmol m^-2^ s^-1^) and blue light (**G**, 10 μmol m^-2^ s^-1^). Bioluminescence values were calculated relative to the value just before the light pulse (Time 0). Mean ± SD of four biological replicates is shown. Arrows indicate approximate time of the light pulse.
(TIF)

S8 Fig ROC15-LUC light response at different wavelengths in *cetl-1/csl* double mutant. The *cetl-1* (mt^-^) mutant was crossed with *csl* mutant (mt^+^) to obtain progenies that inherited *cetl-1* mutation, *csl* mutation and *ROC15-LUC*. Progenies were selected by antibiotic resistance testing and genomic PCRs. Spot cultures were prepared as described in **Fig 4**. Cultures were maintained in darkness for at least 6 hours before being exposed to 5-min pulses of red, blue and violet light **A and B.** Representative traces of WT (top) and *cetl-1/csl* double mutant (bottom) ROC15-LUC response to lights of different wavelength at intensities of 2 μmol m^-2^ s^-1^ (**A**) and 0.2 μmol m^-2^ s^-1^ (**B**). ROC15-LUC bioluminescence values have been calculated relative to the value just before exposure to the light pulse (Time 0). Mean ± SD of five biological replicates are shown. Arrows indicate the approximate time of the light pulse.
(TIF)

S9 Fig ROC15 sub-cellular localization in the *cetl-1* mutant at a lower magnification and light-induced phosphorylation of ROC15 in the complements. Complements that had inherited *ROC15-HA* (but not *ROC15-LUC*), the *cetl-1* mutation, and the *CETL* transgene (Compl.) were obtained from the same genetic cross as described in **Fig 5**. **A.** Cultures were prepared in the same manner as described in **Fig 5**. Cultures were maintained in 6 hours of darkness and immunocytochemistry was performed on cells harvested in darkness. NPC was counterstained as a marker for the nuclear membrane. The BR mt^+^ strain was used as the negative control. Cells were observed at a lower magnification of 10x. Scale bar: 30μm. **B.** Cultures were prepared as described in **Fig 5B**, and exposed to 0.5-min light pulse of blue (**B**, 20 μmol m^-2^ s^-1^) wavelength at midnight (between ZT14 and ZT17). **C.** Unsynchronized TAP cultures were maintained in darkness for 4 hours and exposed to a 0.5-min pulse of red light (**C**, 2 μmol m^-2^ s^-1^). Western blot was performed on whole protein extracts from cells sampled before the light pulse and at 10 minutes after the light pulse. The 0-minute data corresponds to the time point just before samples were exposed to a light pulse. Results of three complemented progenies (1–3) are shown for the red light pulse experiment (**C**). WT in this experiment refers to the wild type sibling from the cross described in **Fig 5**. α-tubulin was used as the loading control.
(TIF)

S10 Fig Circadian rhythm of the *cetl-2* mutant under LL and DD conditions. The *cetl-2* mutant (mt^-^) was crossed with the CBR strain ([mt^+^], WT) to obtain progenies with *cetl-2* mutation and chloroplast luciferase reporter. Progenies were selected based on antibiotic resistance and genomic PCRs. Spot cultures were prepared in the same manner as in **Fig 6. A.** Representative chloroplast bioluminescence rhythm of the *cetl-2* mutant under DD (top) and LL (bottom) conditions. Results from a single trial are shown. 22–25 biological replicates of the WT and 24–30 biological replicates of the *cetl-2* mutant were used for the experiments. Light schedules used are also depicted. **B** and **C.** Distribution of period and amplitude (**B**) and phase diagram of the circadian phases (**C**) of the chloroplast bioluminescence rhythm of the *cetl-2* mutant under DD (top) and LL (bottom) conditions. Data points correspond to the chloroplast bioluminescence rhythm of one *cetl-2* progeny from three trials (74–80 biological replicates). Data points have been shown in comparison to one *cetl-1* progeny and WT from three trials (61–72 and 67–69 biological replicates respectively). *cetl-1* was included in these experiments for comparison. The CT at the beginning of LL was set to 0 and at the beginning of DD was set to 12.
(TIF)

S11 Fig Circadian phases of individual *cetl-1* progeny from different trials under LL conditions. The phase diagram shown in **Fig 6C** (bottom) has been separated to show the data of individual *cetl-1* progeny (1–3) from different trials. As all three *cetl-1* progenies were analyzed at the same time in every trial, the circadian phases of the *cetl-1* progenies within a single trial are compared to the same WT data points. 5–12 biological replicates of WT and 3–12 biological replicates of *cetl-1* progenies were used for the experiment.
(TIF)

S12 Fig Chloroplast bioluminescence rhythms of individual *cetl-1* progeny from different trials under LL conditions. The chloroplast bioluminescence rhythms corresponding to the phase diagrams in **S11 Fig**. The chloroplast bioluminescence traces of *cetl-1* and WT from **Fig 6A** are included in the figure for easy comparison. Since all three *cetl-1* progenies were monitored at the same time in a given trial, the bioluminescence traces of the progenies within a trial are compared to the same WT bioluminescence traces. Each row corresponds to a single trial. 5–12 biological replicates of the WT and 3–12 biological replicates of the *cetl-1* progenies were used in the experiments.
(TIF)

S13 Fig Standard protocol versus single colony protocol. **Standard protocol:** Cells were picked from a single patch on a storage plate and transferred to TAP medium in a 96 well plate. Cultures from the 96 well plate were then spotted on to HS agar plates (Materials and Methods: Culture conditions—Spot cultures for bioluminescence monitoring). **Single colony protocol**: An unsynchronized TAP culture was prepared from a single patch of cells on a storage plate (Materials and Methods: Culture conditions). Cells from this culture were spread onto TAP agar plates and allowed to form colonies. Colonies were picked at random from the plate and used in the preparation of a spot culture. In both protocols, cells were maintained in LL conditions at all stages of preparation until exposure to the appropriate light regime for synchronization.
(TIF)

S14 Fig Phase distribution in the single colony protocol in *cetl-1*. Results from the subsequent trials are shown. Spot cultures were prepared in the same manner as in **Fig 6**. Cultures were maintained in a 12-hour dark period before release into LL conditions. **A.** Chloroplast bioluminescence rhythm after release into LL conditions. Trial 2-Data for 30 biological replicates each for WT and Compl. and 31 biological replicates of *cetl-1*, are shown. Trial 3-Data for 32 biological replicates of WT, 21 biological replicates of Compl., and 30 biological replicates of *cetl-1* are shown**. B.** Circadian phases of the rhythms shown in A. **C.** Plots of amplitude and period of the rhythms show in A. The circadian time (CT) at the beginning of LL was set to 0.
(TIF)

S15 Fig Light response of clock gene mRNAs in the complement. *ROC15* (**A**) and *ROC40* (**B**) mRNA levels in the wild type (WT; ROC15-LUC strain), the *cetl-1* mutant, and the complements (Compl.) were analyzed as described in **Fig 8**. The mean is represented by the bars and the value of each biological replicate is indicated by the dots. These strains possess two copies of *ROC15*: an endogenous copy and the *ROC15-LUC* transgene. The *ROC15* mRNA levels detected in this experiment are therefore a sum of the levels of mRNA from the endogenous copy and the *ROC15-LUC* transgene.
(TIF)

S16 Fig mRNA rhythm of clock genes in the mutant. Free-running high-salt cultures of the wild type (WT; ROC15-LUC strain) and *cetl-1* were harvested every four hours (between 24–72 hours) after release into LL conditions. Mean ± SD of 3 biological replicates are shown at most time points. The levels of *ROC15* mRNA in *cetl-1* mutant at the 56^th^ hour correspond to the mean of two biological replicates. The bar above the graph indicates light conditions (i.e., LL conditions). The alternating white and grey background indicates subjective day and subjective night, respectively, that is expected from the light conditions prior to LL conditions. These strains possess an endogenous copy of *ROC15* and a *ROC15-LUC* transgene; therefore, the *ROC15* mRNA levels detected in this experiment are a sum of the levels of mRNA from the endogenous copy and the *ROC15-LUC* transgene.
(TIF)

S17 Fig Diagrammatic representation of the light signaling pathway for ROC15 degradation. The current working models for the light-induced ROC15 degradation are shown.
(TIF)

S1 Table Details of primers used for insertion check and RT-qPCR. (DOCX)

S2 Table Accession details for the sequences used in the protein sequence alignment (Figs 2B, 2C, S5, and S6). (DOCX)

S1 Data Numerical data corresponding to the main figures. (XLSX)

S2 Data Numerical data corresponding to the supporting figures. (XLSX)
