# Supplementary figures and images for: A potential EARLY FLOWERING 3 homolog in Chlamydomonas is involved in the red/violet and blue light signaling pathways for the degradation of RHYTHM OF CHLOROPLAST 15
Source: PLoS Genet. 2022 Oct 17;18(10):e1010449. doi: 10.1371/journal.pgen.1010449 (PMC9612821; doi:10.1371/journal.pgen.1010449)

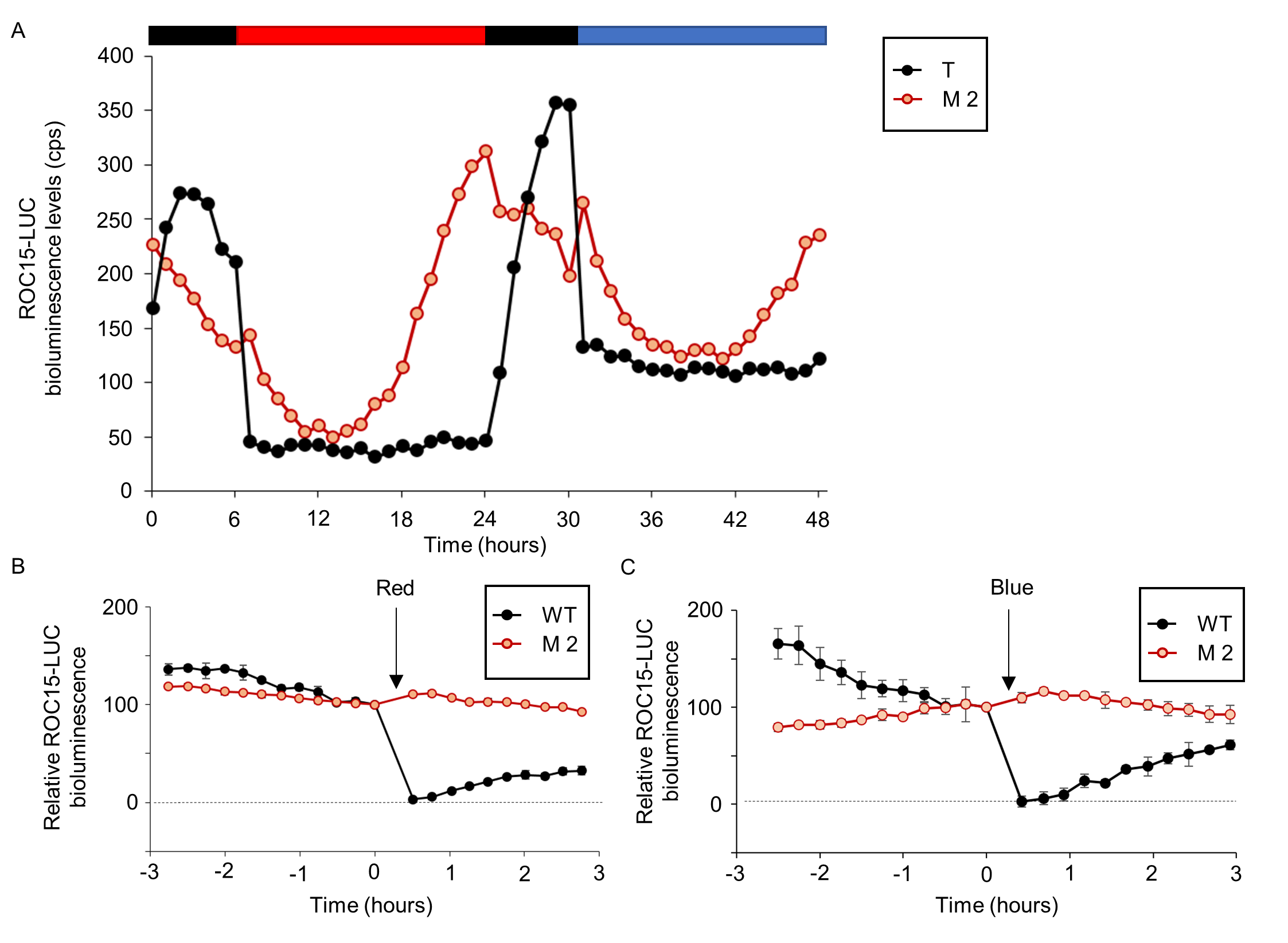

Supplement: S1 Fig — A. ROC15-LUC bioluminescence pattern of the M2 mutant, observed under diurnal conditions. The red, blue, and black bars above the graph represent red light (8 μmol m-2 s-1), blue light (20 μmol m-2 s-1), and dark conditions, respectively. Cells were prepared as described in Materials and Methods (mutant/complement screening). The results of the initial screening are shown. The trace of a transformant (T), exhibiting a WT response, is shown for comparison. The background was not subtracted. B and C. ROC15 bioluminescence response to light pulses in the M2 mutant. Unsynchronized TAP cultures were transferred into 24 well black plates, and their bioluminescence was monitored. Cells were kept in darkness for at least 4–6 hours to allow for the accumulation of ROC15-LUC, and then exposed to a 5-min pulse of red (B, 10 μmol m-2 s-1) and blue (C, 5 μmol m-2 s-1) light. Bioluminescence has been calculated relative to the time point just before the light pulse (Time 0). Mean ± SD of 4 biological replicates are shown. Arrows indicate approximate time of light pulses. (TIF) [file pgen.1010449.s001.tif]

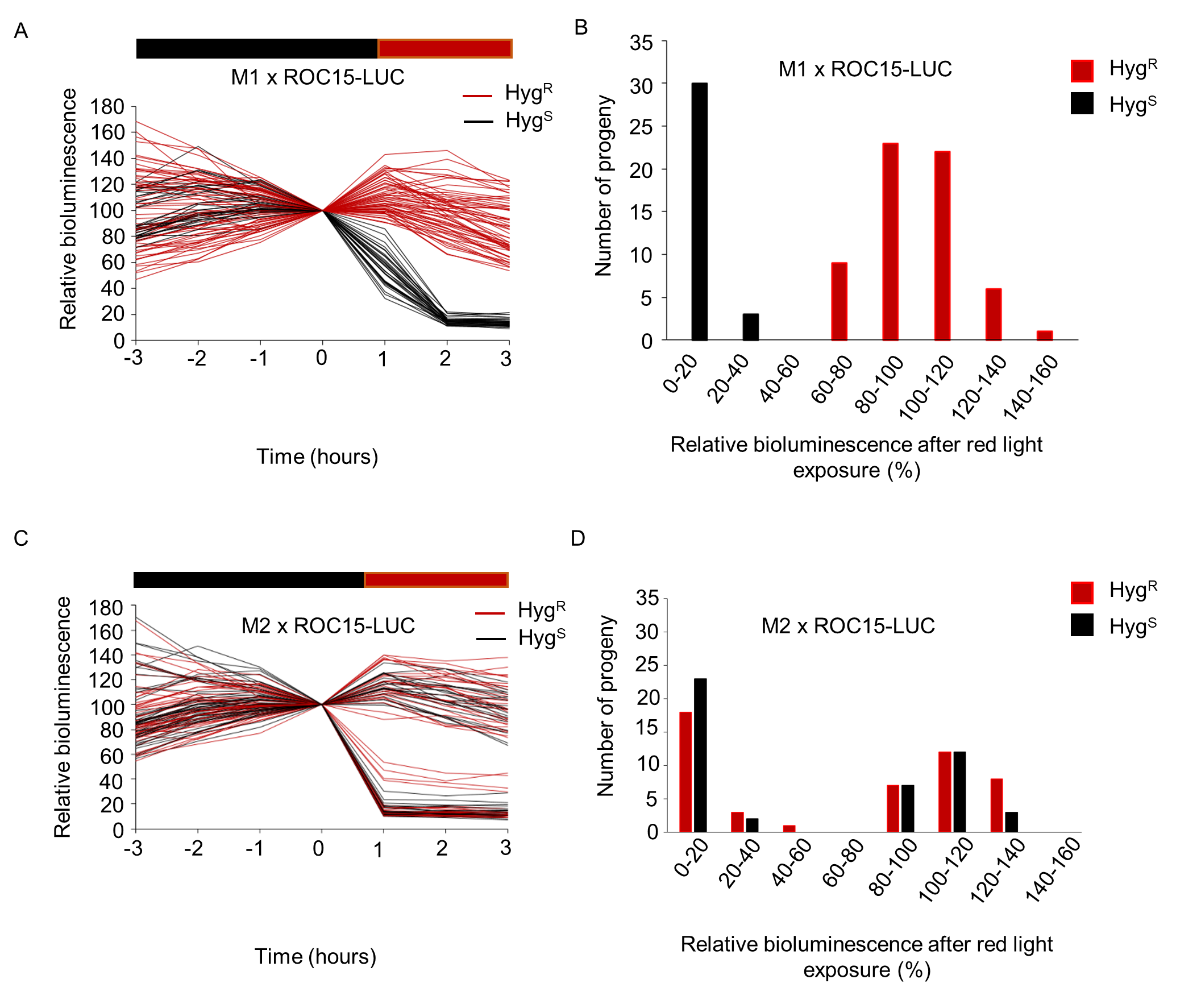

Supplement: S2 Fig — M1 and M2 mutants were backcrossed with the parental ROC15-LUC reporter strain. The progeny were subjected to hygromycin resistance screening and ROC15 bioluminescence monitoring in response to red light (8 μmol m-2 s-1). A and C. Bioluminescence trace of the progeny in response to light, along with the approximate light schedule used. Cells were prepared as described in Materials and Methods (mutant/complement screening). All values were calculated relative to that at the time point (0-hour) just before the start of light phase. The background was not subtracted. Results of 94–96 individual progeny have been plotted. B and D. Distribution of the numbers of progeny showing hygromycin sensitivity, and their relative bioluminescence levels after exposure to light (corresponding to hour 2 in A and C). (TIF) [file pgen.1010449.s002.tif]

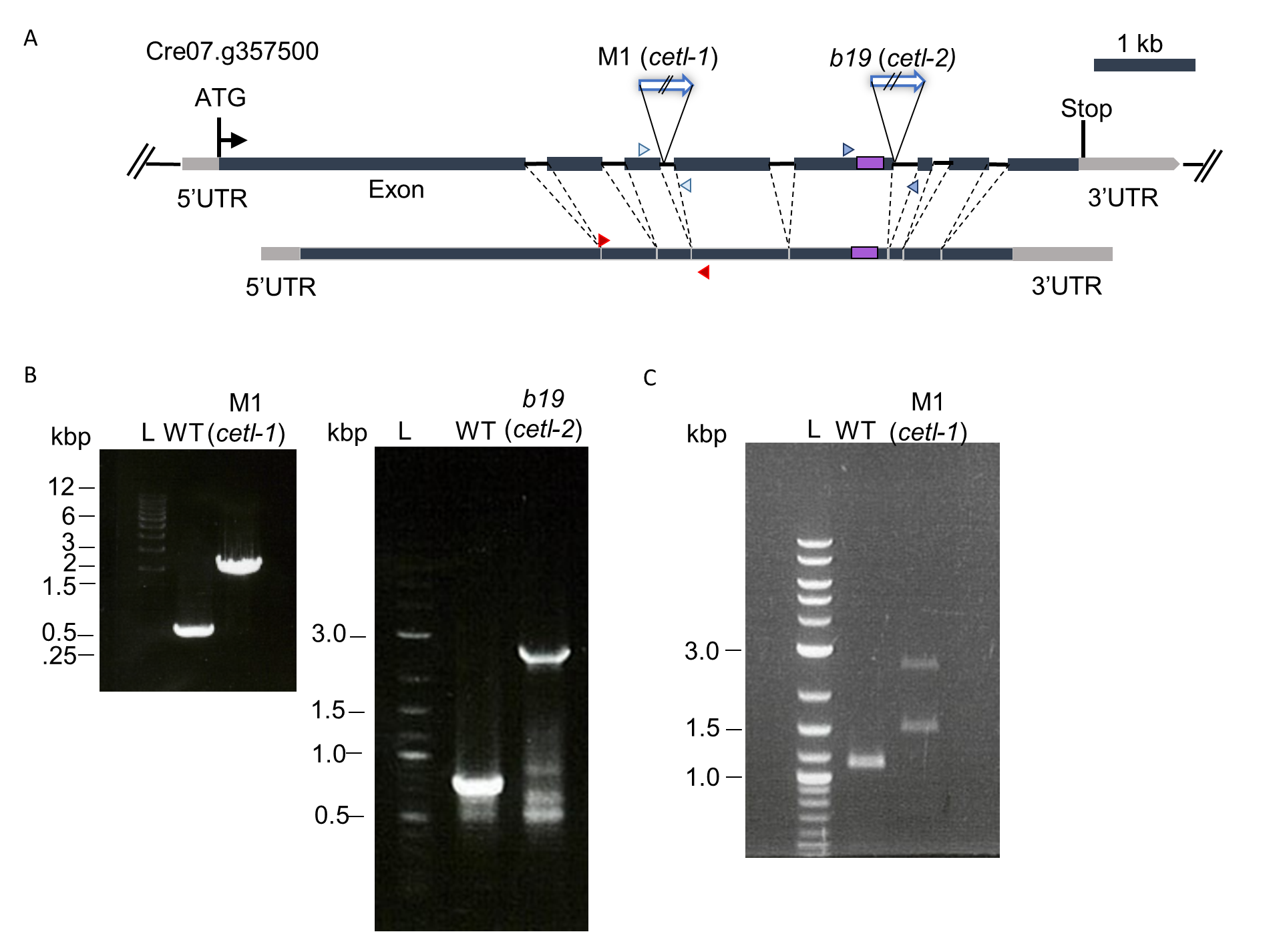

Supplement: S3 Fig — Primer binding sites and PCR confirmation of the insertion of the hygromycin resistance gene (aph7”). A. Schematic representation of the Cre07.g357500 gene and its transcript. Blue triangles around the insertion loci indicate the binding sites of the primers used for PCR confirmation in the genomic DNA. The red triangles indicate the binding sites of the primers used for PCR confirmation in the cDNA. B. PCR was performed on the wild type (WT) and mutant genomic DNA with primers targeted around the insertion loci (depicted in A). The amplified products were visualized on an agarose gel. C. RT-PCR was performed on the Cre07.g357500 transcript in the WT and mutant with primers targeted to the loci depicted in A. The amplified products were visualized on an agarose gel. The primers used are listed in S1 Table. (TIF) [file pgen.1010449.s003.tif]

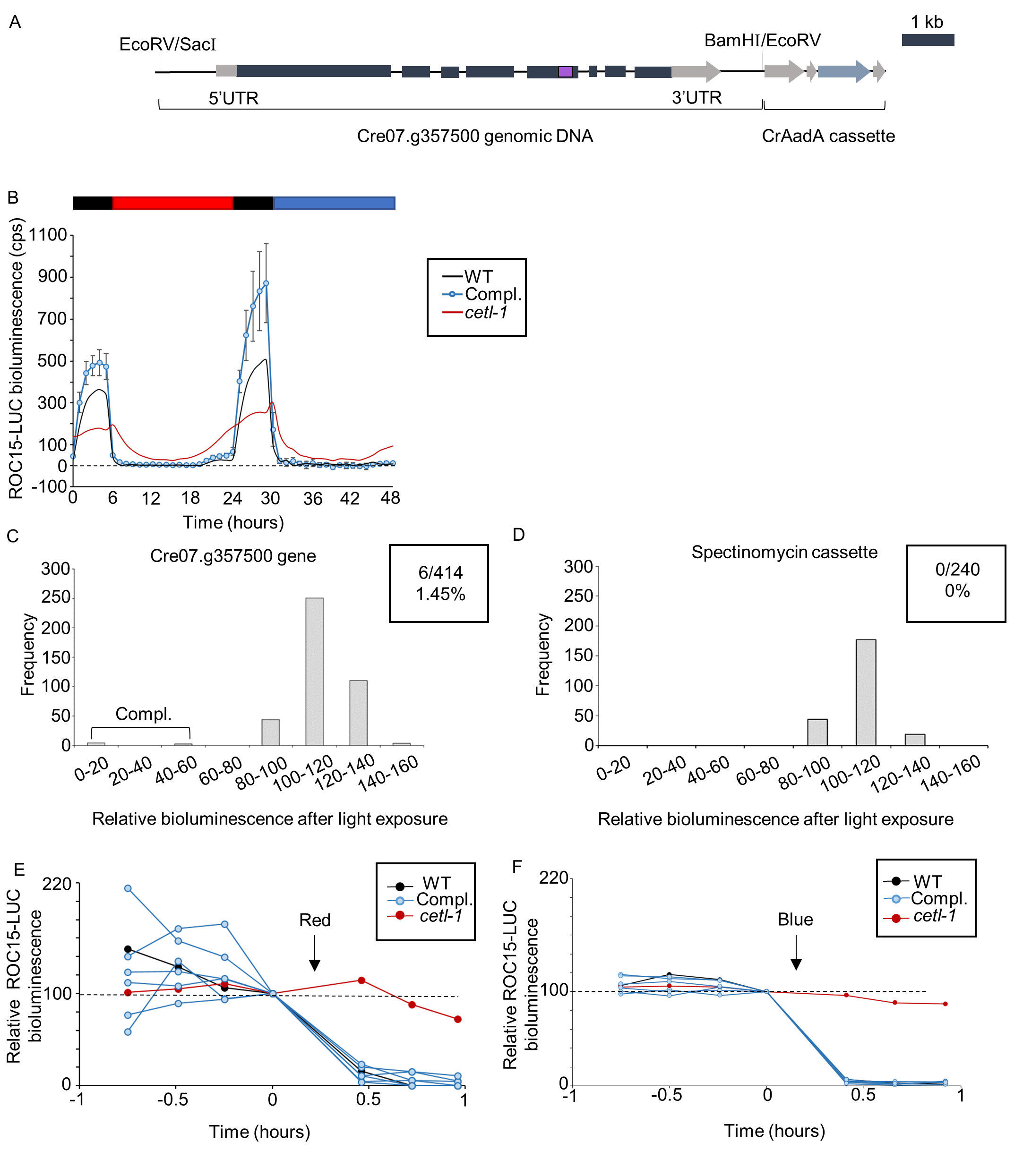

Supplement: S4 Fig — A. Schematic representation of the gene fragment used for complementation. WT genomic DNA fragment of Cre07.g357500 gene ligated to the spectinomycin resistance cassette (aadA) was used for complementation. B. A representative ROC15-LUC bioluminescence pattern of one of the six complements. Culture preparation and light conditions were the same as in Fig 1A. Mean ± SD of 8 biological replicates of the complement (Compl.) have been plotted. The bioluminescence trace of the WT and M1 from Fig 1A have been plotted for comparison. C and D. Histograms of the distribution of transformants with respect to the relative bioluminescence levels after exposure to red light (8 μmol m-2 s-1). The bioluminescence values at the first time point after the start of red light exposure were taken relative to the values just before the start of red light exposure. Transformants with only aadA are shown as a negative control. E and F. Representative trace of the light-pulse response of the six isolated complements. Unsynchronized TAP cultures were transferred into 24 well black plates and their bioluminescence was monitored as described in the Materials and Methods. Cells were kept in darkness for at least 3 hours before being exposed to a 5-min pulse of red light (E, 10 μmol m-2 s-1) and blue light (F, 10 μmol m-2 s-1). Bioluminescence values have been calculated relative to the time point just before the light pulse (Time 0). Background was not subtracted. Arrows indicate the approximate time of the light pulse. (TIF) [file pgen.1010449.s004.tif]

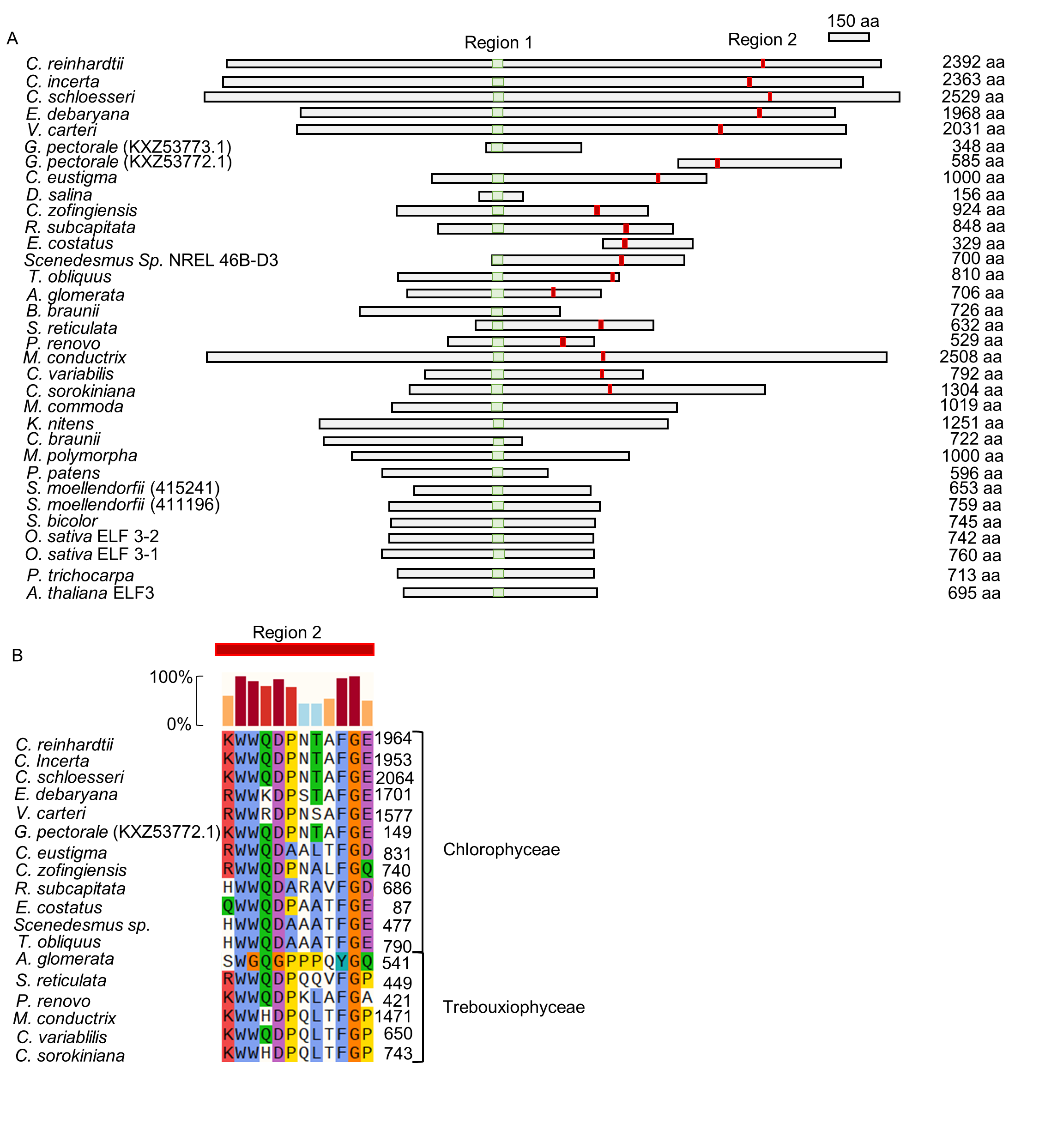

Supplement: S5 Fig — A. Schematic representation of all the protein sequences used in the alignment and the two regions of similarity (Region 1 and Region 2). B. Alignment of the sequences at Region 2. Amino acids have been colored according to their properties and conservation (Clustal X color scheme). Percentage of conservation at each site is indicated by colored bars above the alignment. Sequences were aligned in the same manner as in Fig 2. Amino acid sequences used for the alignment are the same as those used in Fig 2. (TIF) [file pgen.1010449.s005.tif]

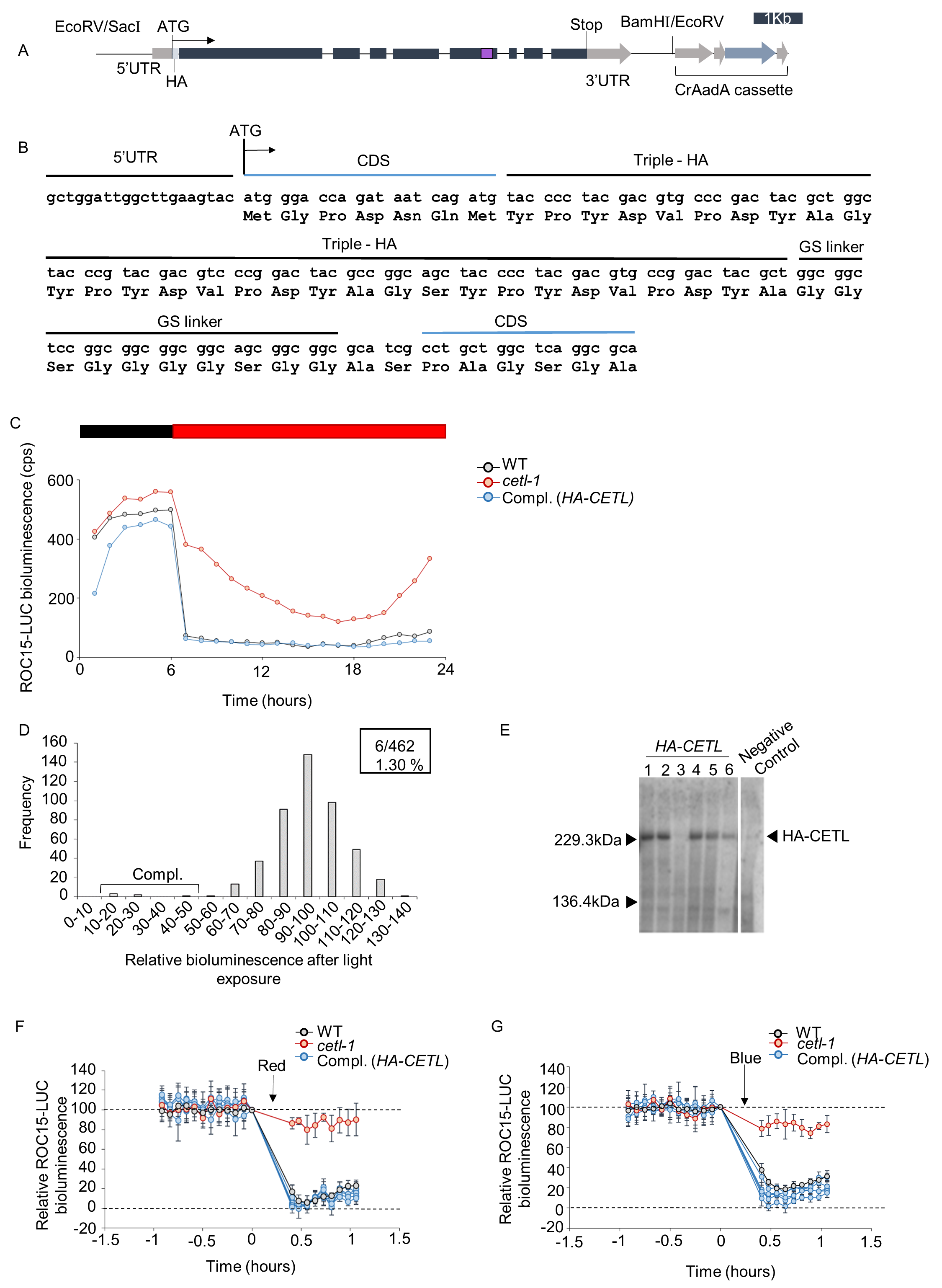

Supplement: S7 Fig — A. Schematic representation of the gene fragment used for complementation. An HA tag, codon adapted for the C. reinhardtii nuclear genome was incorporated after the second predicted start codon of the CETL gene that was ligated to aadA. B. The codon adapted sequences of the HA tag and a flexible GS linker. C. Representative trace of the ROC15-LUC bioluminescence pattern of one of six complements. Culture preparations were the same as in Fig 1A. Cultures were subjected to 6 h dark /18 h red light (2 μmol m-2 s-1) cycle. The representative trace of one of the six complements is shown in comparison to the traces of the WT and cetl-1 (n = 1). D. Histogram showing the distribution of the transformants with respect to the ROC15 bioluminescence levels at the first time point after red light exposure. Bioluminescence levels were calculated relative to the value just before light exposure. E. Western blot analysis of the complemented strains. Unsynchronized TAP cultures were maintained in darkness for 6 hours. Western blot was performed on whole protein extracts from cells harvested in darkness. The WT BR mt+ strain was used as the negative control. F and G Light pulse response of five of the six complements. Spot cultures were prepared as described in Fig 4 and their bioluminescence was monitored as described in materials and methods. Cells were in darkness for at least 6 hours before being exposed to 5-min pulses of red (F, 5 μmol m-2 s-1) and blue light (G, 10 μmol m-2 s-1). Bioluminescence values were calculated relative to the value just before the light pulse (Time 0). Mean ± SD of four biological replicates is shown. Arrows indicate approximate time of the light pulse. (TIF) [file pgen.1010449.s007.tif]

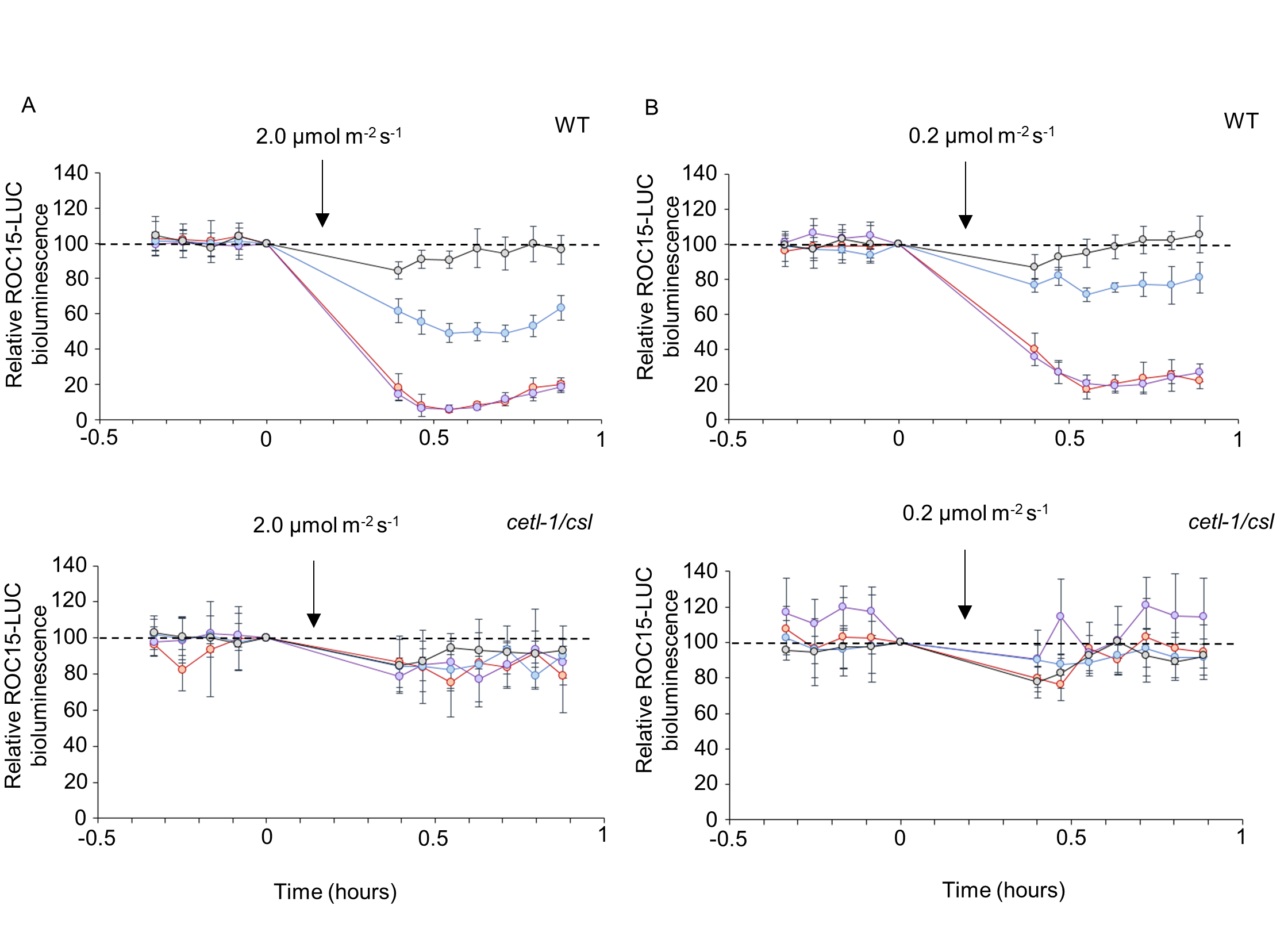

Supplement: S8 Fig — The cetl-1 (mt-) mutant was crossed with csl mutant (mt+) to obtain progenies that inherited cetl-1 mutation, csl mutation and ROC15-LUC. Progenies were selected by antibiotic resistance testing and genomic PCRs. Spot cultures were prepared as described in Fig 4. Cultures were maintained in darkness for at least 6 hours before being exposed to 5-min pulses of red, blue and violet light A and B. Representative traces of WT (top) and cetl-1/csl double mutant (bottom) ROC15-LUC response to lights of different wavelength at intensities of 2 μmol m-2 s-1 (A) and 0.2 μmol m-2 s-1 (B). ROC15-LUC bioluminescence values have been calculated relative to the value just before exposure to the light pulse (Time 0). Mean ± SD of five biological replicates are shown. Arrows indicate the approximate time of the light pulse. (TIF) [file pgen.1010449.s008.tif]

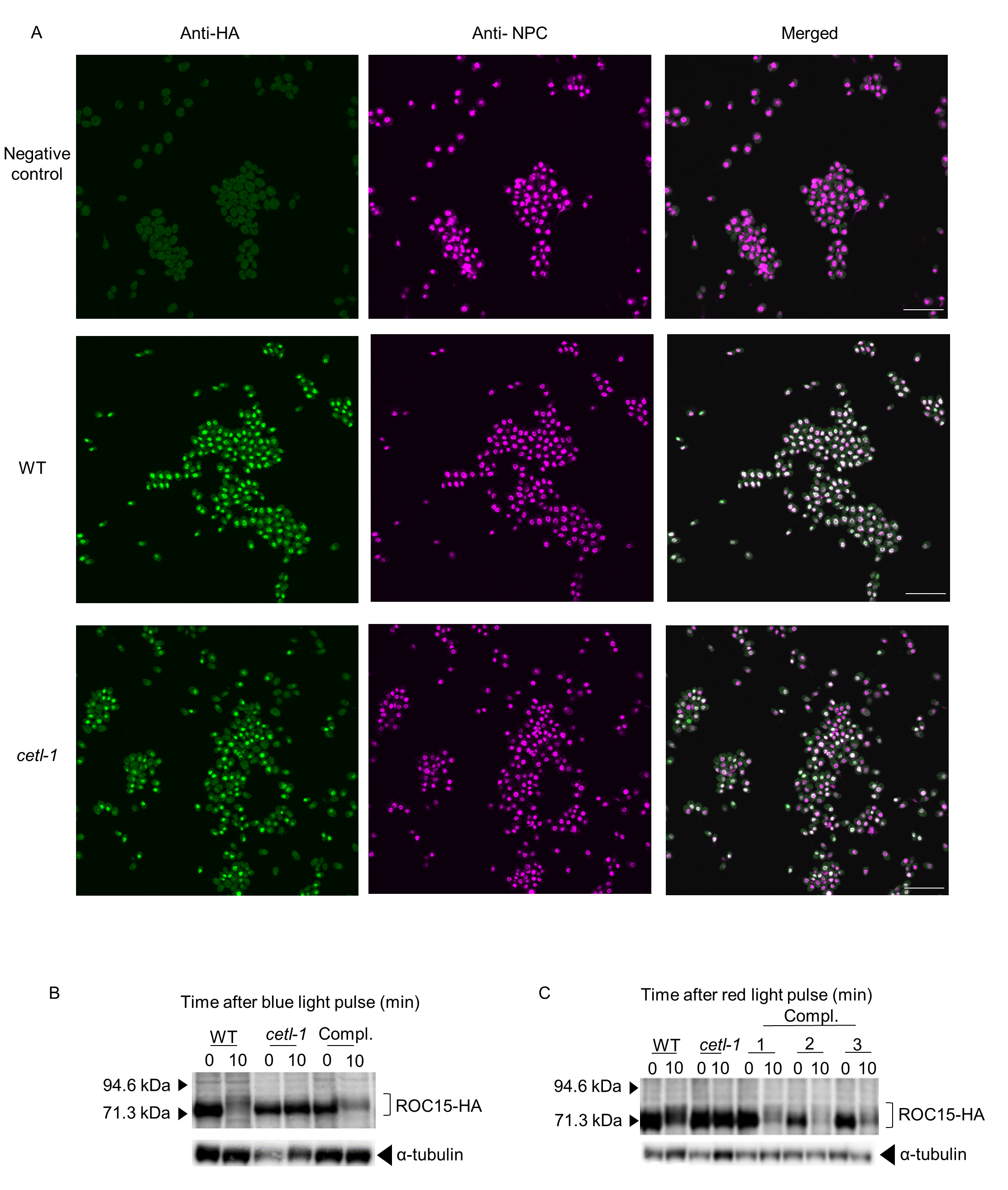

Supplement: S9 Fig — Complements that had inherited ROC15-HA (but not ROC15-LUC), the cetl-1 mutation, and the CETL transgene (Compl.) were obtained from the same genetic cross as described in Fig 5. A. Cultures were prepared in the same manner as described in Fig 5. Cultures were maintained in 6 hours of darkness and immunocytochemistry was performed on cells harvested in darkness. NPC was counterstained as a marker for the nuclear membrane. The BR mt+ strain was used as the negative control. Cells were observed at a lower magnification of 10x. Scale bar: 30μm. B. Cultures were prepared as described in Fig 5B, and exposed to 0.5-min light pulse of blue (B, 20 μmol m-2 s-1) wavelength at midnight (between ZT14 and ZT17). C. Unsynchronized TAP cultures were maintained in darkness for 4 hours and exposed to a 0.5-min pulse of red light (C, 2 μmol m-2 s-1). Western blot was performed on whole protein extracts from cells sampled before the light pulse and at 10 minutes after the light pulse. The 0-minute data corresponds to the time point just before samples were exposed to a light pulse. Results of three complemented progenies (1–3) are shown for the red light pulse experiment (C). WT in this experiment refers to the wild type sibling from the cross described in Fig 5. α-tubulin was used as the loading control. (TIF) [file pgen.1010449.s009.tif]

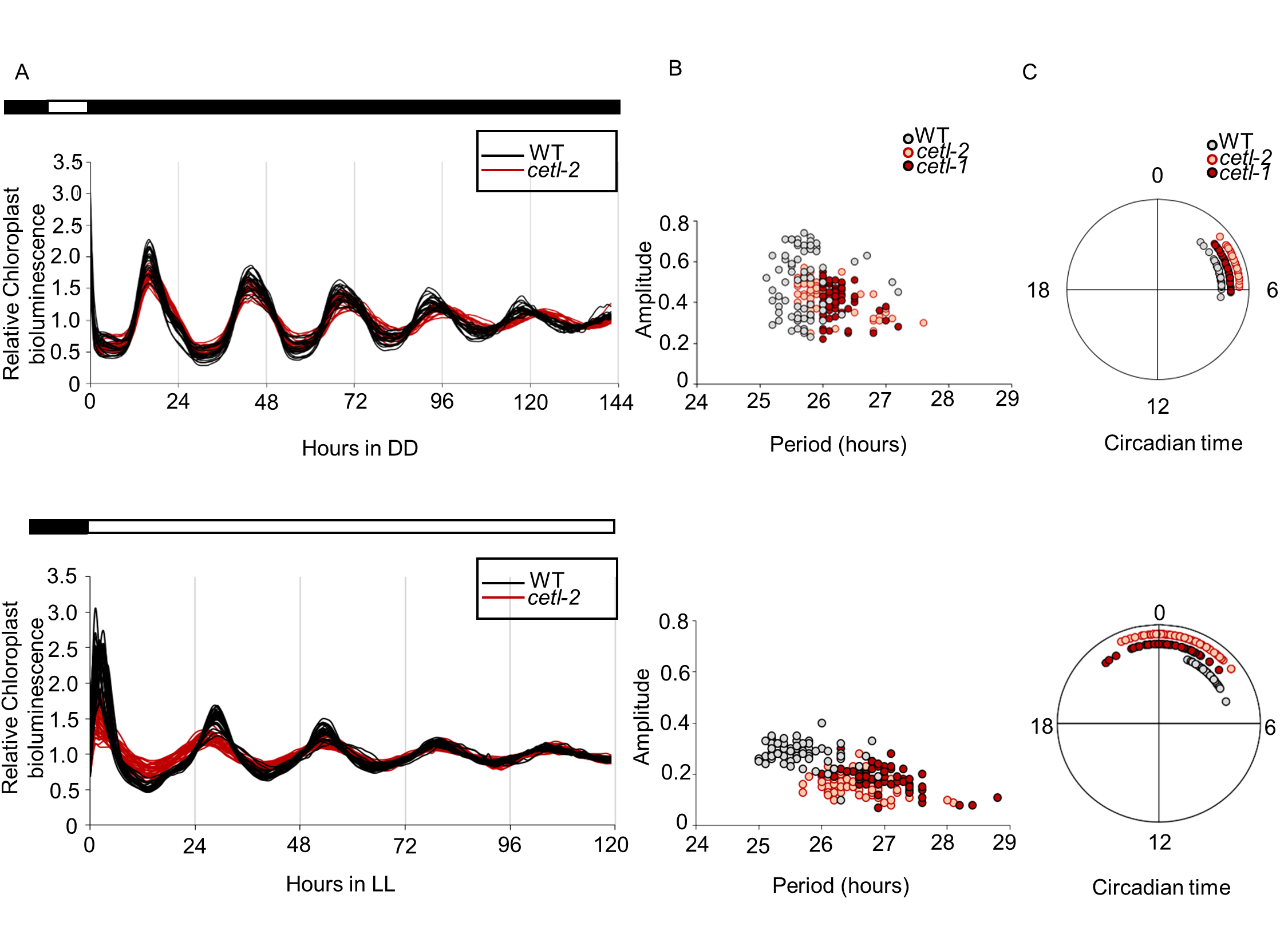

Supplement: S10 Fig — The cetl-2 mutant (mt-) was crossed with the CBR strain ([mt+], WT) to obtain progenies with cetl-2 mutation and chloroplast luciferase reporter. Progenies were selected based on antibiotic resistance and genomic PCRs. Spot cultures were prepared in the same manner as in Fig 6. A. Representative chloroplast bioluminescence rhythm of the cetl-2 mutant under DD (top) and LL (bottom) conditions. Results from a single trial are shown. 22–25 biological replicates of the WT and 24–30 biological replicates of the cetl-2 mutant were used for the experiments. Light schedules used are also depicted. B and C. Distribution of period and amplitude (B) and phase diagram of the circadian phases (C) of the chloroplast bioluminescence rhythm of the cetl-2 mutant under DD (top) and LL (bottom) conditions. Data points correspond to the chloroplast bioluminescence rhythm of one cetl-2 progeny from three trials (74–80 biological replicates). Data points have been shown in comparison to one cetl-1 progeny and WT from three trials (61–72 and 67–69 biological replicates respectively). cetl-1 was included in these experiments for comparison. The CT at the beginning of LL was set to 0 and at the beginning of DD was set to 12. (TIF) [file pgen.1010449.s010.tif]

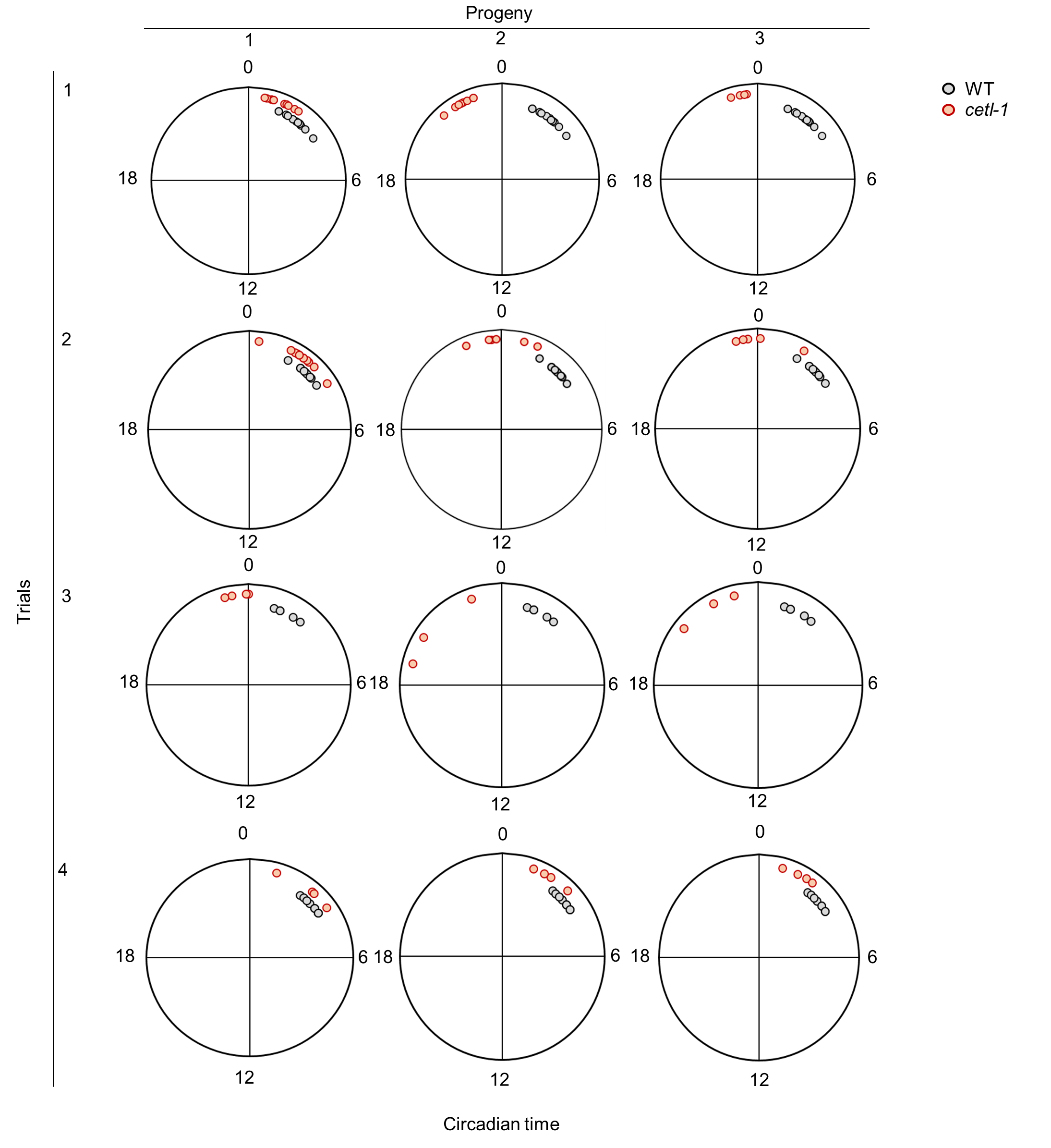

Supplement: S11 Fig — The phase diagram shown in Fig 6C (bottom) has been separated to show the data of individual cetl-1 progeny (1–3) from different trials. As all three cetl-1 progenies were analyzed at the same time in every trial, the circadian phases of the cetl-1 progenies within a single trial are compared to the same WT data points. 5–12 biological replicates of WT and 3–12 biological replicates of cetl-1 progenies were used for the experiment. (TIF) [file pgen.1010449.s011.tif]

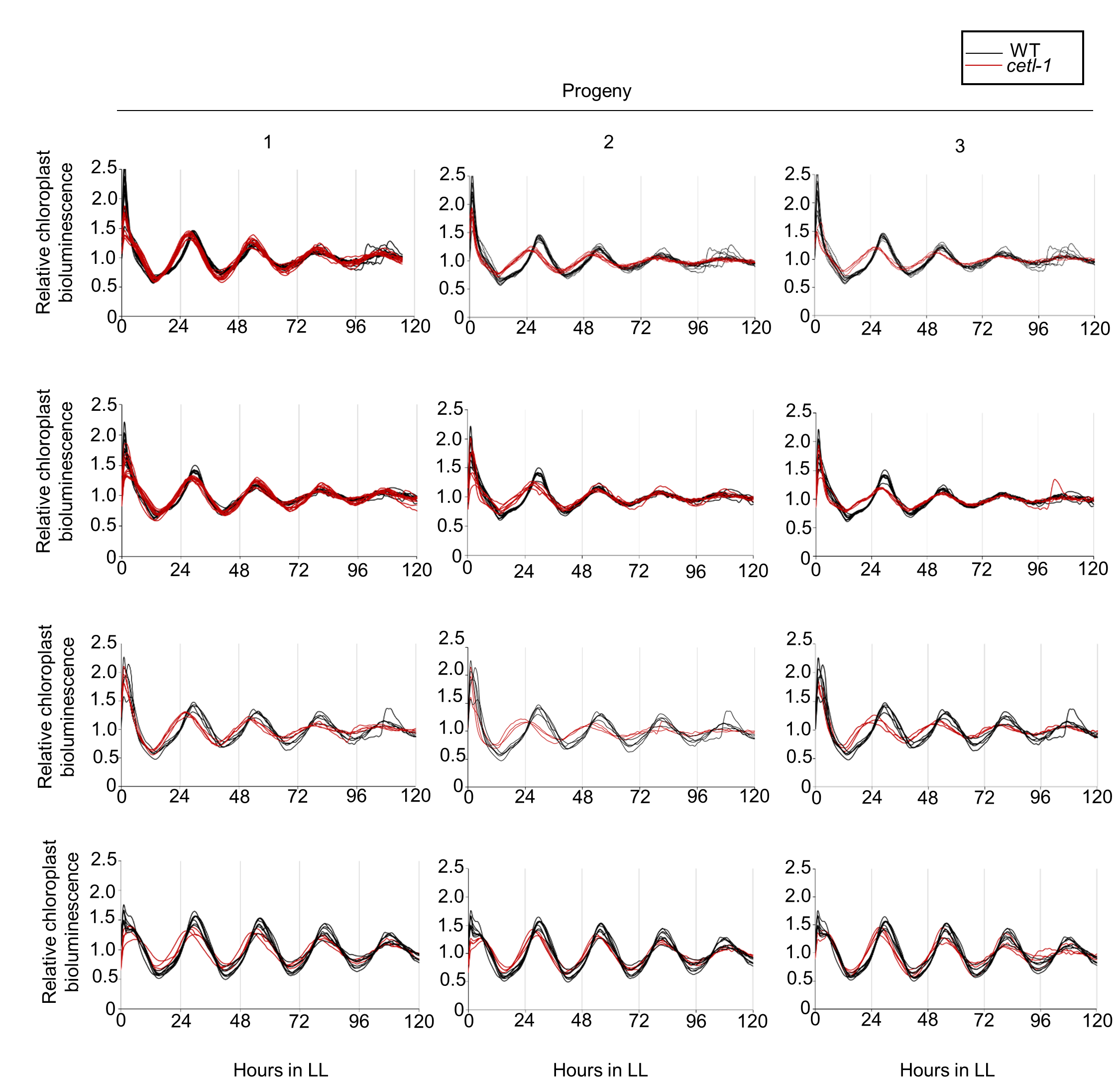

Supplement: S12 Fig — The chloroplast bioluminescence rhythms corresponding to the phase diagrams in S11 Fig. The chloroplast bioluminescence traces of cetl-1 and WT from Fig 6A are included in the figure for easy comparison. Since all three cetl-1 progenies were monitored at the same time in a given trial, the bioluminescence traces of the progenies within a trial are compared to the same WT bioluminescence traces. Each row corresponds to a single trial. 5–12 biological replicates of the WT and 3–12 biological replicates of the cetl-1 progenies were used in the experiments. (TIF) [file pgen.1010449.s012.tif]

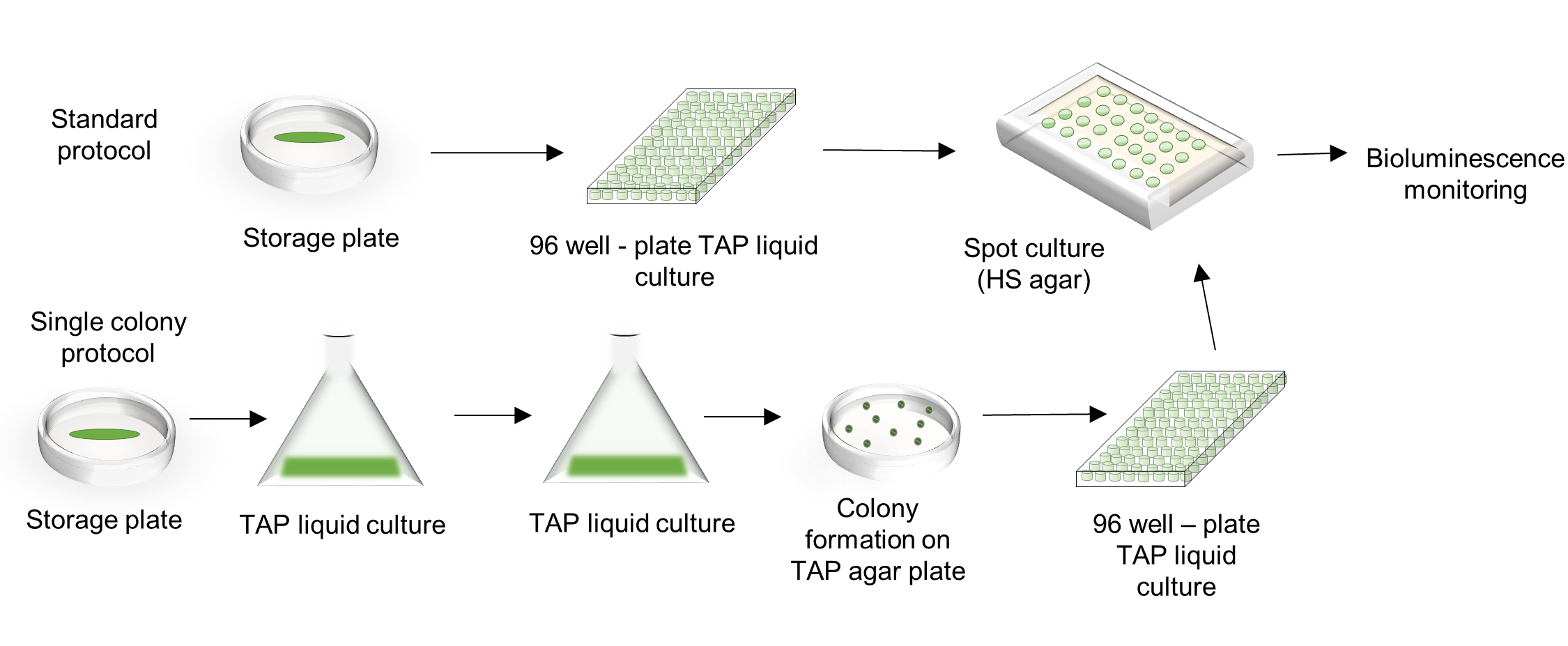

Supplement: S13 Fig — Standard protocol: Cells were picked from a single patch on a storage plate and transferred to TAP medium in a 96 well plate. Cultures from the 96 well plate were then spotted on to HS agar plates (Materials and Methods: Culture conditions—Spot cultures for bioluminescence monitoring). Single colony protocol: An unsynchronized TAP culture was prepared from a single patch of cells on a storage plate (Materials and Methods: Culture conditions). Cells from this culture were spread onto TAP agar plates and allowed to form colonies. Colonies were picked at random from the plate and used in the preparation of a spot culture. In both protocols, cells were maintained in LL conditions at all stages of preparation until exposure to the appropriate light regime for synchronization. (TIF) [file pgen.1010449.s013.tif]

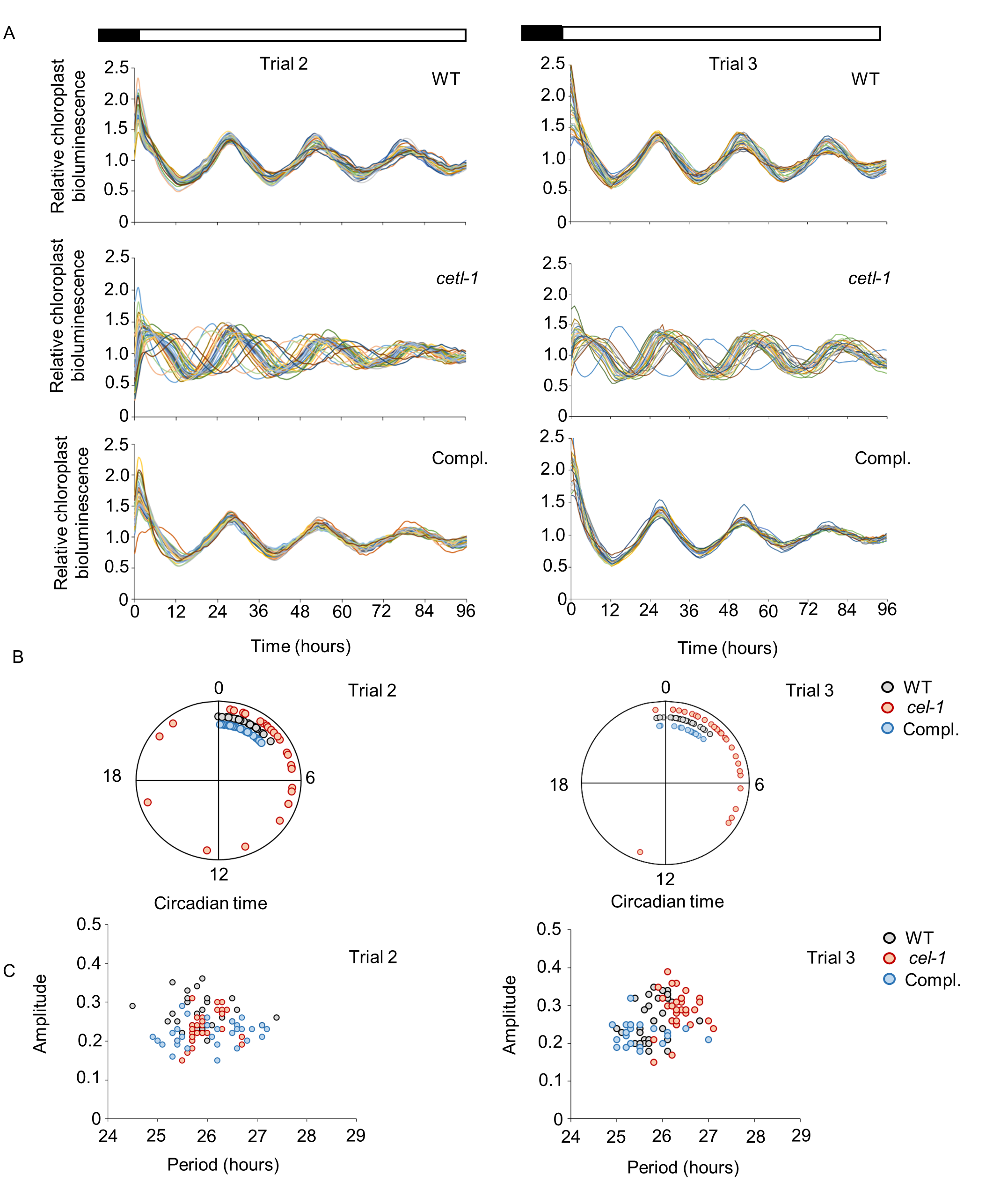

Supplement: S14 Fig — Results from the subsequent trials are shown. Spot cultures were prepared in the same manner as in Fig 6. Cultures were maintained in a 12-hour dark period before release into LL conditions. A. Chloroplast bioluminescence rhythm after release into LL conditions. Trial 2-Data for 30 biological replicates each for WT and Compl. and 31 biological replicates of cetl-1, are shown. Trial 3-Data for 32 biological replicates of WT, 21 biological replicates of Compl., and 30 biological replicates of cetl-1 are shown. B. Circadian phases of the rhythms shown in A. C. Plots of amplitude and period of the rhythms show in A. The circadian time (CT) at the beginning of LL was set to 0. (TIF) [file pgen.1010449.s014.tif]

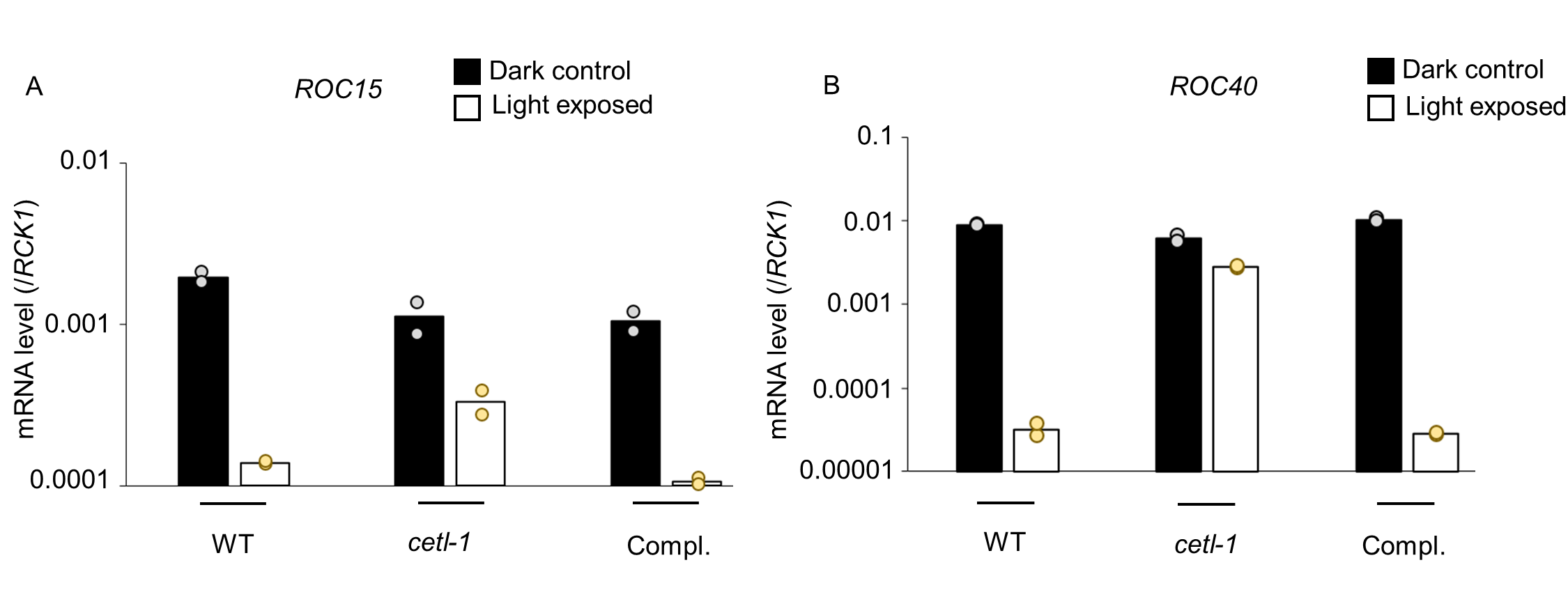

Supplement: S15 Fig — ROC15 (A) and ROC40 (B) mRNA levels in the wild type (WT; ROC15-LUC strain), the cetl-1 mutant, and the complements (Compl.) were analyzed as described in Fig 8. The mean is represented by the bars and the value of each biological replicate is indicated by the dots. These strains possess two copies of ROC15: an endogenous copy and the ROC15-LUC transgene. The ROC15 mRNA levels detected in this experiment are therefore a sum of the levels of mRNA from the endogenous copy and the ROC15-LUC transgene. (TIF) [file pgen.1010449.s015.tif]

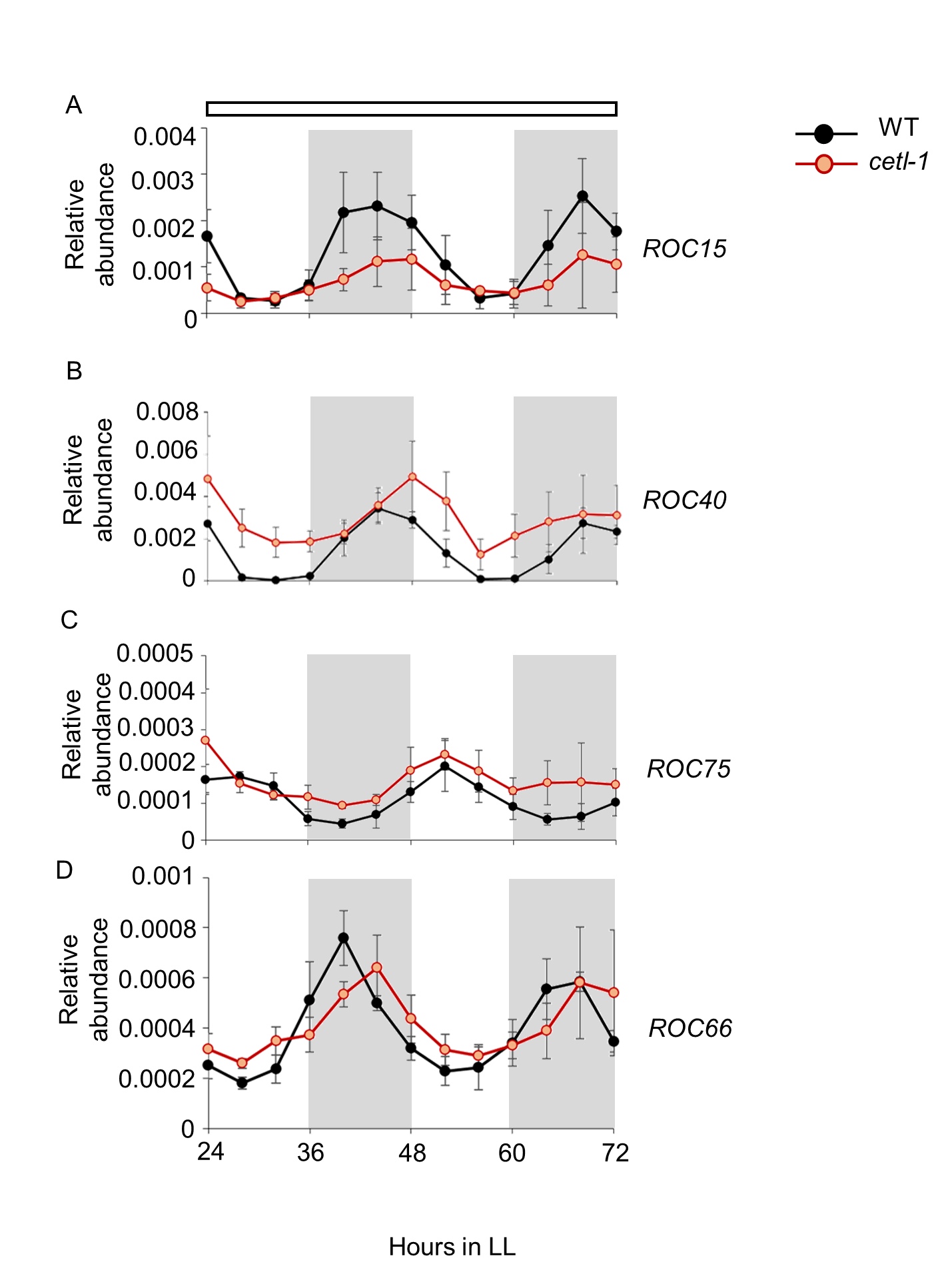

Supplement: S16 Fig — Free-running high-salt cultures of the wild type (WT; ROC15-LUC strain) and cetl-1 were harvested every four hours (between 24–72 hours) after release into LL conditions. Mean ± SD of 3 biological replicates are shown at most time points. The levels of ROC15 mRNA in cetl-1 mutant at the 56th hour correspond to the mean of two biological replicates. The bar above the graph indicates light conditions (i.e., LL conditions). The alternating white and grey background indicates subjective day and subjective night, respectively, that is expected from the light conditions prior to LL conditions. These strains possess an endogenous copy of ROC15 and a ROC15-LUC transgene; therefore, the ROC15 mRNA levels detected in this experiment are a sum of the levels of mRNA from the endogenous copy and the ROC15-LUC transgene. (TIF) [file pgen.1010449.s016.tif]

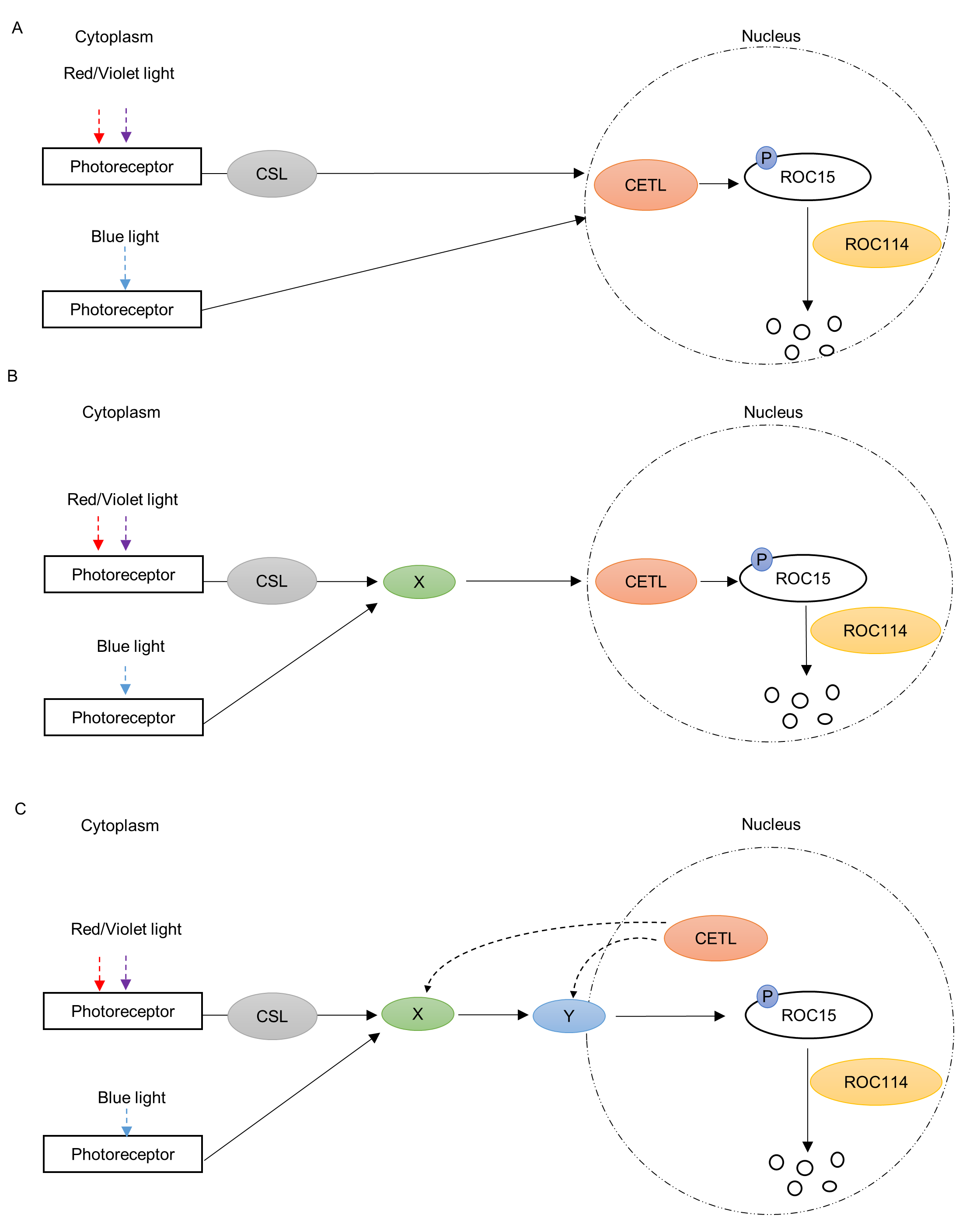

Supplement: S17 Fig — The current working models for the light-induced ROC15 degradation are shown. (TIF) [file pgen.1010449.s017.tif]
