# Supplementary material for: A potential EARLY FLOWERING 3 homolog in Chlamydomonas is involved in the red/violet and blue light signaling pathways for the degradation of RHYTHM OF CHLOROPLAST 15
Source: PLoS Genet. 2022 Oct 17;18(10):e1010449. doi: 10.1371/journal.pgen.1010449 (PMC9612821; doi:10.1371/journal.pgen.1010449)
Supplement: S6 Fig — Protein encoded by Cre07. g357500 was aligned with A. thaliana ELF3 and its homologs from O. sativa (ELF3-1), S. lycopersicum and Z. mays using MAFFT (MAFFT 7.471, SnapGene) and MUSCLE (MUSCLE 3.8.1551, SnapGene) multiple sequence alignment algorithms. Block I, II, III, and IV are highlighted according to the regions recognized as Blocks in A. thaliana ELF3 [11]. Conserved and similar amino acids are represented in the Clustal format. Region 1 and Region 2 are underlined in red and orange respectively, in both alignments. Details of the amino acid sequences can be found in S2 Table. (PDF) [file pgen.1010449.s006.pdf]

Aligned using MAFFT

```
C. reinhardtii      MGPDNQMPAGSGAVGKSQLAVPKQPQGGLPAGSGPHLGQSAGAPQVFAPGLAGATAAAAA  60
O. sativa           -----MATRGGGGGGGGK----------------------EAKGKVMGP-----------  22
A. thaliana         ------MKRGK------------------------------DEEKILEP-----------  13
S. lycopersicum     ------MKRGK------------------------------GEEKVMGP-----------  13
Z. mays             ------MRRGATKDDA-------------------------APDKVMGP-----------  18
                          :.:*.                              . .::..*  Block I

C. reinhardtii      MLGQLQSSGAML-SGPQPQLPLAAPAAQQHQQALQQYLLQQTQQLQQQLQQQQQQQQQQ  119
O. sativa           LFPRLHVNDAAKGGGPR--------APPRNKMALYEQFTVPSHRFSG-------------  61
A. thaliana         MFPRLHVNDADK-GGPR--------APPRNKMALYEQLSIPSQRFG--------------  50
S. lycopersicum     MFPRLHVNDTDK-GGPR--------APPRNKMALYEQLSIPSQRFN--------------  50
Z. mays             LFPRLHVNDTLK-GGPR--------APPRNKMALYEQFSVPSHRYS--------------  55
                    ::::*::::: . :**: Block I*::::::**::::..:::::.

C. reinhardtii      RLQLAAGQGGGGVAPCISPVLQHLQLHRQLQAAAAGTSQTSQNSLSLGPGGQMQLLPPSF  179
O. sativa           ----GGGGGGVGGSP-----------------AHSTSAASQSQSQSQVYGRDSSLFQP--  98
A. thaliana         ------DHGTMNSRS------------------NNTSTLVHPGPSSQPCGVERNL-----  81
S. lycopersicum     -------SGVLPLDP-------------------NNTSKMAPPSSSQGSGHDRSGYLP--  82
Z. mays             --------AAVPPAPSPAPPW----------GAQRPASAVPSTSASQVGGGDRPIFPL--  95
                            :. . .:                   .... . . : *:. * ....  .

C. reinhardtii      PQMPGRYPQLPGLVQAPQQAAAASGALMALSSLMAAGGVAGPVPGLAPPGVHGVPSGGMR  239
O. sativa           -------------FNVPSNR----------------------------------------  105
A. thaliana         ------------------------------------------------------------  81
S. lycopersicum     -------------IQHPPSRRLAD------------------------------------  93
Z. mays             -------------FRVPSTE----------------------------------------  102
                                    .

C. reinhardtii      PMGPGQLPLAAAPLLASTGRPGMPQPGAAGPPFFGGPGGPVPGVGGLQVPLPVPYGGGRP  299
O. sativa           ------------------------------------------------------------  105
A. thaliana         ------------------------------------------------------------  81
S. lycopersicum     ----------------------------------------------------------KP  95
Z. mays             ------------------------------------------------------------  102

C. reinhardtii      PGHMALGPGGGSAAGGGDSAVA----LLQQWQLQQLQQLQQQALLPGLPPAAASAFLNGA  355
O. sativa           PGHSTEKINSDKINKKISGSRKELGMLSSQTKGMDIYASRSTA-----------------  148
A. thaliana         SVQHLDSSAANQATEK----------FVSQMSFME--NVRSSA-----------------  112
S. lycopersicum     PGHSSDPST-----------------LLQQYE----------------------------  110
Z. mays             PVRSSDQTNANSNGQGANGTIAESG---RQRQSTHLKSKDTNA-----------------  142
                    :.:... ..                 . .* .          .

C. reinhardtii      MLGAGMQQQQQQPALSGHKPPLQQPLQYALPQPSLVRRPVAVAAGGSPSLQLPPPQAEGG  415
O. sativa           --------------------------------------------------EAPQRRAENT  158
A. thaliana         --------------------------------------------------QHDQ------  116
S. lycopersicum     ------------------------------------------------------------  110
Z. mays             --------------------------------------------------AGPPAEGNNS  152

C. reinhardtii      MARSAPAAQAPAVKPESSRQVGDMATAAAAAAAAAGDHYRNGSGSG-DRHGP--------  466
O. sativa           IKSS---------SGKRLADDDEFMVPSVFNSRFPQYSTQENAGVQ-DQSTPLVAANPHK  208
A. thaliana         --------------RKMVREEEDFAVPVYINSRRSQSHGRTKSGIEKEKHTPMVA-----  157
S. lycopersicum     --------------LKKRTEEDDFTVPIFVNSKLGQAHGSHNVNME--KLS---------  145
Z. mays             V-------------GKKLANDDDFTVPSVLYSGMPPHSSQE-------KLTLFPTTSPCK  192
                                   :  ....::.::.  .:. .. . ..  .    :...

C. reinhardtii      --PSPQQ--QRQPLPQPSRQLQAPQVQQTSGESYSTVTEGSTEASSGATSAGCSPPGAGG  522
O. sativa           S-PSTVSKSSTKCYNTVSKKLE--RIHVSDVKSRTPLKDKEMEA----------------  249
A. thaliana         --PSSHH----------SIRFQ--EVNQTGSKQNVCLATCSKP-----------------  186
S. lycopersicum     --PSGQL-------FCPNKELE--GVTHLTLRQQRNSQNKE-------------------  175
Z. mays             SVPAKYS-------STDKRRLE--GMDASDVKSKGPSGIKEKEP----------------  227
                      *: .           ...::  .:  .. ..   .  ..

C. reinhardtii      TTEAASPRQYDKSLGGGGTGSPPSGGRRLAGPAHPAGAAGVRADGYKRRRDDWEEEEEVG  582
O. sativa           ----------------------------------------------AQTSKNVE-VEKSS  262
A. thaliana         -----------------------------------------------EVRDQVKANARSG  199
S. lycopersicum     -----------------------------------------------NLKCTLARREKTT  188
Z. mays             ----------------------------------------------VQVRINLEDKETTP  241
                                                                   . . ...  :...
```

```
C. reinhardtii      PPHCAKALEAAAVAAGATPGGPLDGSETSGGLASGGGGGVTGADGN---GSGGSTTLGAP  639
O. sativa           SFHASKDM-FESRHAKVYPKMDKTGIINDSDEPHGGNSGHQATSRN-----GGSMKFQNP  316
A. thaliana         GFVI-------SLDVSVTEEIDLEKSASSHDRVNDYNASLRQESRNRLYRDGGKTRLKDT  252
S. lycopersicum     SNSA-------SKECRLDPQV-------------------------------GCSSIPEP  210
Z. mays             SFQVLNDK-TWSPDPKLSSHMDRL------KKQHAEAESYQIRTRN-----ENAVETQSP  289
                    .. .       : .... . .                        ..    .:.. .  :

C. reinhardtii      AQRRSLSDEVAAARAARAEDARQQQRQRAGRSGTSDA-GSGSRENASSGMRDVQLAEGGS  698
O. sativa           PMRRN-----EISSNPSSENTDRHYN----------LPQGGIEETGTKRKRLLEQH----  357
A. thaliana         DNGA--------ESHLATENHSQEGHGSP-----EDI----DNDREYSKSR---------  286
S. lycopersicum     VKGTY-----DGSSYPRKEFVSEEQLTA------NDL----VNDTESQEDR---------  246
Z. mays             PKNGV-----SLLSKPY---VDREQNGD------SDLLGHGLRETGEKRKR---------  326
                     ...         : .  .  ....          ..     .:.. .. *

C. reinhardtii      NGGAAAGSDAMQVDMRWQQTQAPTAAQAQTQAQQAGRGSAGSAGGGRGEVRGYGREAGRR  758
O. sativa           ---DAEKSD---------------------------------------------------  363
A. thaliana         ----ACAS----------------------------------------------------  290
S. lycopersicum     ----AHKS----------------------------------------------------  250
Z. mays             ------------------------------------------------------------  326
                        .  .

C. reinhardtii      GEPDTDMAEAEGEGEGQSETECSDTPLTSSSSQSQSQSQQRGAASQRQQHQGGPAGMQAG  818
O. sativa           -----DVSRLLEQHDAENIDDVSDSS-VECITGWE-------------------------  392
A. thaliana         ----------LQQINEEASDDVSDDSMVDSISSID-------------------------  315
S. lycopersicum     ----------LQTGNLDRGDDLSETSRVESISGTD-------------------------  275
Z. mays             -----------SHHDVEQNDDLSDSS-VESLPGME-------------------------  349
                           .. . :  ::.*:.: :.:... .

C. reinhardtii      GRGFREGEGEGGRMGASPLPASEAAVGAAGADDGVARAGGGSRDGSGGVAFPTRGNDGTT  878
O. sativa           ---------------ISP-------------DKIVG------------------------  400
A. thaliana         ---------------VSP-------------DDVVG------------------------  323
S. lycopersicum     ---------------ISP-------------DDIVG------------------------  283
Z. mays             ---------------ISP-------------DDVVS------------------------  357
                               .**             *:.*.
                                                             Block II

C. reinhardtii      AAAAAAVTAAAAGLSLRAAPPQPWGEQAQLPPQFQPHQLQHQQHQQPMFSPPPPGGHMMM  938
O. sativa           ------------------------------------------------------------  400
A. thaliana         ------------------------------------------------------------  323
S. lycopersicum     ------------------------------------------------------------  283
Z. mays             ------------------------------------------------------------  357
                                              Block II

C. reinhardtii      PPPPAFPPGAGPVGPGGPLPPPPPGPPAPAGGPALRDLLGDPLYGAVRATLMRQQSVFVM  998
O. sativa           -------------------------------------AIGTKHFWKARRAIMNQQRVFAV  423
A. thaliana         -------------------------------------ILGQKRFWRARKAIANQQRVFAV  346
S. lycopersicum     -------------------------------------IIGLKRFWKARRAIVNQQRVFAI  306
Z. mays             -------------------------------------AIGPKHFWKARRAIVNQQRVFAV  380
                              Block II                    .:* :.::.:*.::.:**:**::

C. reinhardtii      QLGELHKIARVQQVIWSEMLVVDPAGLQAACMEAGFAGAGPAGPILRLPPPPAPGQQQQQ  1058
O. sativa           QVFELHKLVKVQKLI-------------------------AASPHVLIESDPCLGNALLG  458
A. thaliana         QLFELHRLIKVQKLI-------------------------AASPDLLLDEISFLGKVSAK  381
S. lycopersicum     QVFELHRLIKVQRLI-------------------------AGSPNSSLEDPAYLGKPLKS  341
Z. mays             QVFELHRLIKVQKLI-------------------------AASPHVLIEGDPCLGKSLAV  415
                    *:.***::.:**::*       Block II          :::* ..:.  ..:*. .

C. reinhardtii      QQQQRPLTPQPHMGLGTATPESQEGGGVAAGEQQHQRQRPGLASVAGAAEAGYQLTSATT  1118
O. sativa           SK--NKL---------------------------------------------------VEE  466
A. thaliana         SYPVKKL---------------------------------------------------LPS  391
S. lycopersicum     SS-IKRLP-----------------------------------------------LDCIV  353
Z. mays             SK--KRLA-----------------------------------------------GDVET  426
                    :   ..*                                                    .

C. reinhardtii      AHEAQA--GSVVASAAVEAAGGYGGGAGTSSQPHHGAQRSAPAAPPPPAAPPLSREELRV  1176
O. sativa           NLKAQP-----LLVATIDD------VEPSLQQPEVSKENTEDSPPSPH------------  503
A. thaliana         EFLVKPPLPHVVVKQRGDS----------EKTDQHKMESSAENVVGR-------------  428
S. lycopersicum     R-ESQS-----VLKRKHDS-----------EKPHFRMEHTAESNVGK-------------  383
Z. mays             QLES----------AKNDDG-----VRP--KQLEHSKEKTEANQPSP-------------  456
                      ...        .  .. :.           ...... :.:... ...
```

```
C. reinhardtii      RLLGDMQLLFNLPRGLRERVLRSVPPEQLVRPAVPPPPAPRPAATMVGGGGGLGGGGNAV  1236
O. sativa           ------------------------------------------------------------  503
A. thaliana         ------------------------------------------------------------  428
S. lycopersicum     ------------------------------------------------------------  383
Z. mays             ------------------------------------------------------------  456


C. reinhardtii      VRPAASKLYTLMDGDEGPADSEQPTVVQKVVAQATARAAAAAAEKQRNNGGSGGGSVAGG  1296
O. sativa           --------------------------------------DTGLGSGQRDQAATNGVSKSNR  525
A. thaliana         ------------------------------------------LSNQ------GHHQQSNY  440
S. lycopersicum     ----------------------------------------ASLSTVQ------NGSQLSSH  398
Z. mays             --------------------------------------------SQDEQAATNGAVAASM  472
                                                                 *      .:  .  :.

C. reinhardtii      PNGGIPAAVGQAGPFRQQPTAAPPSPHAAQPATRLGLPDGAAAAVPSPHQVLPPFPPHGM  1356
O. sativa           R-------------------ATPVA-----------------------------------  531
A. thaliana         M------------PFANNPPASP-------------------------------------  451
S. lycopersicum     K------------PFSGTPLPTPVT-----------------------------------  411
Z. mays             H--------------------TP-------------------------------------  475
                                        ..*

C. reinhardtii      PQRPQASALLMPPPSMHPHMHPPSPAAAFGLPPHALPAGPGLAQPHALLGGMPPAAAAAA  1416
O. sativa           ---------------------SDNKQNNWGVQL---------------------------  543
A. thaliana         ------------------------APNGYCFPP---------------------------  460
S. lycopersicum     ---------------------NDSNAGPWCFQ----------------------------  422
Z. mays             ---------------------SDNKQKSWCI-----------------------------  485
                                         ..  ...

C. reinhardtii      GLAVGPPPHGMGAAGAAAAATAGAGAFGSPGSSFRAPPHPAQPGQPLHMDLPPQWSGPAM  1476
O. sativa           ----QPPQN-------------------------------------------QWLVPVM  555
A. thaliana         ----QPPPSG---------------------------NH-------------QQWLIPVM  476
S. lycopersicum     ----QPPGH-------------------------------------------QWLIPVM  434
Z. mays             ----PAPPS-------------------------------------------QWLIPVM  497
                        .:*..                                            **:.*:*
                                                     Block III

C. reinhardtii      -PQHG--HLPLHGQLPPHMHMAMPLHGQLPPGMAGPLGALPAR--GPLQGGAPGAPPPGG  1531
O. sativa           SPLEGLVYKPYSGPCPPAGSILAPFYANCTP------LSLPSTA-GDFMNSAYGV-----  603
A. thaliana         SPSEGLIYKPHPGM-------------------------AHTGHYGGYYGHYMPT-----  506
S. lycopersicum     SPSEGLVYKPFPGP-----GFTSPICGSGPP------GSSPTM--GNFFAPTYGV-----  476
Z. mays             SPSEGLVYKPYTGHCPPVGSLLAPPFFASYP------TSSSSTAGGDFMSSACGA-----  546
                    .*.:*..:.*..*          .      .       ....   *.: ...  :.
                     Block III

C. reinhardtii      APMAVAAGGPRGPLQQPQQPQPLVRPPGVPPPLVGTPPLPMPQPGPSAAAPVPHLDAAGT  1591
O. sativa           -------------------------------------PMPH-QPQ-HMGAPGP-------  617
A. thaliana         -------------------------------------PMVMPQYHPGMGFP---------  520
S. lycopersicum     -------------------------------------PAPNPHYQ-GMGVPFA-------  491
Z. mays             -------------------------------------RL--------MSAP---------  552
                                                         :..  .    :..*

C. reinhardtii      SQPQSAAAPAPTPAAPVPGGGFGGVLPPALPVLFSSRQGAGVGAGGTKVPPVLRPPGVPA  1651
O. sativa           ---------------PSMPMNY---FPP-----FSI--------------PVMNPTAPA-  639
A. thaliana         ----------------PPGNGY---FPP-----YGMM------------PTIMNPYCSSQ  544
S. lycopersicum     ---------------PPTGHGY---FRQ-----YGM--------------PAMNPPISS-  513
Z. mays             --------------------VY---FPS-----FSM--------------PAVSGSA---  567
                                      . .:   ::.     ::.              :.:.:

C. reinhardtii      PAIPVPRPVASIDPHEAWFARHVAGAGAGAQPAAAQPAAATPAAAAEAAGAAGPAAGAVG  1711
O. sativa           ------------------------------------------------------------  639
A. thaliana         ------------------------------------------------------------  544
S. lycopersicum     ------------------------------------------------------------  513
Z. mays             ------------------------------------------------------------  567


C. reinhardtii      SRQAQAQAPSQGLGSRPGSLQRIASTERVVGGGGGSAAAVTGTGALQQPPERQSQPQQRQ  1771
O. sativa           -----PVVEQGRHPSMPQPYGNF----------------------------------EQQS  661
A. thaliana         QQQQQQPNEQMNQFGHPGNLQNT---------------------------------QQQQ  571
S. lycopersicum     -----TASEESNQYTMPGLQHQFSGVVDDV-------------------------NIQHQD  544
Z. mays             -------VEQVSHVAASQHKRN--------------------------------------  582
                           .:. .. . :.  ..                                  ...
```

```
C. reinhardtii      GAEEAEAAPSAARGGPSADVGWVAAARQGQPHQPRPASEMQAGAPQDQAPSASQPEGLEA  1831
O. sativa           WISCNMSHPS--------GIWRFHASRDS----------------EAQASSASSPFD---  694
A. thaliana         QRSDNEPAPQQQQ-QPTKSYPRARKSR--------------------QGSTGSSPSG---  607
S. lycopersicum     --SSNVLNQKKEN-VP--DVVRYQSTKDN----------------EVQASSASSP-----  578
Z. mays             --SCSEA---------------VLASRDS----------------EVQGSSASSP-----  604
                      : ... .            .  ..:.                . *::::*:*

C. reinhardtii      QGQQELQTGAPGVAGAPAAPPDVYAVAGGSAGGAPGQGQAQAASSDDDEEEDVATSAGAN  1891
O. sativa           ----------------------------------------------------RFQCSGSG  702
A. thaliana         ----------------------------------------------------PQGISGSK  615
S. lycopersicum     -----------------------------------------------------IETAGRN  585
Z. mays             ------------------------------------------------------------  604
                                                                         .

C. reinhardtii      ALQPPPSLPPLHQAEAAAGLAAAAAAAPAAAAAAPTSSGAAAPPPPPPPSAAVPVPAVGP  1951
O. sativa           PVSAFPT---------------------------VSAQNNQPQPSYSSRDNQ--------  727
A. thaliana         SFRPFAA--------------------------VDEDSNINNAPEQTMTTTTT-------  642
S. lycopersicum     MLSLFPT--------------------------SPVTDNRDGSPQACVPDNP--------  611
Z. mays             -----------------------------------ASSETAAQP----------------  613
                          .                           ... ...*

C. reinhardtii      TKWWQDPNTAFGELGLVDPQALGNTPAVVEDDEEDDDDEAAAAAATAGLQPGDALAAHMQ  2011
O. sativa           ------------------------------------------------------------  727
A. thaliana         -----------------------TTRTTVTQTTRD-------------------------  654
S. lycopersicum     ------------------------------------------------------------  611
Z. mays             ------------------------------------------------------------  613

C. reinhardtii      LVQSLVPAPRAAGSGEGSGSGAPPPLPPLKKREKYMMQQQQQQQQQAAAVAAAAAAAAGG  2071
O. sativa           ------------------------------------------------------------  727
A. thaliana         ------------------------------------------------------------  654
S. lycopersicum     ------------------------------------------------------------  611
Z. mays             ------------------------------------------------------------  613

C. reinhardtii      APPGAAPGQGAAALAGANQAAMAAAAAVALGLDPATAAAAAALMASGVAGGLGPGGMGGG  2131
O. sativa           ------------------------------------------------------------  727
A. thaliana         ---------------------------------------------------------GGG  657
S. lycopersicum     ------------------------------------------------------------  611
Z. mays             ------------------------------------------------------------  613

C. reinhardtii      VRPEVLLAATRMLQPSAFKTVPPRQQHSNRGSNRLTMSTHTGATESMASGWGFGLSRRAG  2191
O. sativa           ---------TNVI-----KVVP----HNSR-----------TASESAARIF---------  749
A. thaliana         --------VTRVI-----KVVP----HNAK-----------LASENAARIF---------  680
S. lycopersicum     ---------ARVI-----KVVP----HNAR-----------SATESVARIF---------  633
Z. mays             ----------RVI-----RVVP----HTAR-----------TASESAARIF---------  634
                             .:::     ::**    *..:  Block IV  *:*:.*:.:

C. reinhardtii      GGAQPERSEAGVSRATGGAGPHYRTSGNGGATTSAAGGGAGRPARKVERKYDGEASEAGG  2251
O. sativa           ------------------------------------------------------------  749
A. thaliana         ------------------------------------------------------------  680
S. lycopersicum     ------------------------------------------------------------  633
Z. mays             ------------------------------------------------------------  634

                                             Block IV

C. reinhardtii      GAASAASGGRRPEAQQAQQRPQQQQQQRQQQQGARGGITAGGAAATGAEGTQGGAEADEA  2311
O. sativa           ---------------------RSIQMERQRDD----------------------------  760
A. thaliana         ---------------------QSIQEERKRYDSSK-------------------------  694
S. lycopersicum     ---------------------QSIQQERNNMT----------------------------  644
Z. mays             ---------------------RSIQMERKQNDP---------------------------  646
                          Block IV       ::.*.:*.. .

C. reinhardtii      QTSGAGGGGGGAGGRAAGGAAGKVRRAGEGPSVAAAAAAGVGGAVRHPQQRAVARAGGGD  2371
O. sativa           ------------------------------------------------------------  760
A. thaliana         -----------------------------------------------P------------  695
S. lycopersicum     ------------------------------------------------------------  644
Z. mays             ------------------------------------------------------------  646
```

C. reinhardtii	DVGEVSSGAVEAAEAEDGMSE	2392
O. sativa	---------------------	760
A. thaliana	---------------------	695
S. lycopersicum	---------------------	644
Z. mays	---------------------	646

Aligned using MUSCLE

```
C. reinhardtii       MGPDNQMPAGSGAVGKSQLAVPKQPQGGLPAGSGPHLGQSAGAPQVFAPGLAGATAAAAA  60
O. sativa            ------------------------------------------------------------  0
A. thaliana          ------------------------------------------------------------  0
S. lycopersicum      ------------------------------------------------------------  0
Z. mays              ------------------------------------------------------------  0


C. reinhardtii       MLGQLQSSGAMLSGPQPQLPLAAPAAQQHQQALQQYLLQQTQQLQQQLQQQQQQQQQQQR  120
O. sativa            ------------------------------------------------------------  0
A. thaliana          ------------------------------------------------------------  0
S. lycopersicum      ------------------------------------------------------------  0
Z. mays              ------------------------------------------------------------  0


C. reinhardtii       LQLAAGQGGGGVAPCISPVLQHLQLHRQLQAAAAGTSQTSQNSLSLGPGGQMQLLPPSFP  180
O. sativa            ---MATRGGGGGGG--------------------------------GKEAKGKVMGPLFP  25
A. thaliana          ----MKRGKD----------------------------------------EEKILEPMFP  16
S. lycopersicum      ----MKRGKG----------------------------------------EEKVMGPMFP  16
Z. mays              ----MRRGATK-----------------------------------DDAAPDKVMGPLFP  21
                         ..:*..                                        . :::.*.**
                                                                          Block I
C. reinhardtii       QMPGRYPQLPGLVQAPQQAAAASGALMALSSLMAAGGVAGPVPGLAPPGVHGVPSGGMRP  240
O. sativa            RLH---------------------------------------------------------  28
A. thaliana          RLH---------------------------------------------------------  19
S. lycopersicum      RLH---------------------------------------------------------  19
Z. mays              RLH---------------------------------------------------------  24
                     :::                          Block I

C. reinhardtii       MGPGQLPLAAAPLLASTGRPGMPQPGAAGPPFFGGPGGPVPGVGGLQVPLPVP---YGGG  297
O. sativa            -------------VNDAAKG-------------GGPRAPPRNKMALYEQFTVPSHRFSGG  62
A. thaliana          -------------VNDADK--------------GGPRAPPRNKMALYEQLSIPSQRFGDH  52
S. lycopersicum      -------------VNDTDK--------------GGPRAPPRNKMALYEQLSIPSQRFNSG  52
Z. mays              -------------VNDTLK--------------GGPRAPPRNKMALYEQFSVPSHRYSAA  57
                                  :::: :   Block I    ***::*:::..:*::::.:*...:...

C. reinhardtii       RPPGHMALGPGGGSAAGGGDSAVALLQQWQLQQLQQLQQQALLPGLPPAAASAFLNGAML  357
O. sativa            GGGGGVGGSP--AHST--------------------------------------------  76
A. thaliana          G-----TMNSRSNNTS--------------------------------------------  63
S. lycopersicum      V----LPLDP--NNTS--------------------------------------------  62
Z. mays              VPPAPSPAPPWGAQRP--------------------------------------------  73
                           .  :  ....

C. reinhardtii       GAGMQQQQQQPALSGHKPPLQQPLQYALPQPSLVRRPVAVAAGGSPSLQLPPPQAEGGMA  417
O. sativa            -SAASQSQSQSQVYGRDSSLFQPFNV-------------------------PSNRPGHST  110
A. thaliana          -TLVHPGPSSQPC-GVERNL--SVQH----------------------------------  85
S. lycopersicum      -KMAPPSSSQGS--GHDRSGYLPIQH-------------------------PPSR-----  89
Z. mays              -ASAVPSTSASQVGGGDRPIFPLFRV-------------------------PSTEP---V  104
                      . . ...:...  * ....  ....                         .

C. reinhardtii       RSAPAAQAPAVKPESSRQVGDMATAAAAAAAAAGDHYRNGSGSGDRHGPPSPQQQRQPLP  477
O. sativa            EKINSDKINKKISGSRKELGMLSSQTKGMDIYASRSTAEAPQRRAENTIKSSSGKRLA--  168
A. thaliana          --LDSSAANQATEKFVSQMSFMENVRSSAQ-----------------------HDQRKMV  120
S. lycopersicum      ------RLADKPPGHSSDPSTLLQQYE--------------------------LKKRT--  115
Z. mays              RSSDQTNANSNGQGANGTIAESGRQRQSTHLKSKDTNAAGPPAEGNNSV----GKKLA--  158
                            ..   ..  .... .  .                             ....

C. reinhardtii       QPSRQLQAPQVQQTSGESYSTVTEGSTEASSGATSAGCSPPGAGGTTEAASPRQYDKSLG  537
O. sativa            -DDDEFMVP-----------SVFNSRFPQYSTQENAGVQDQST--PLVAANPHKSPSTVS  214
A. thaliana          REEEDFAVP-----------VYINSRRSQSHGRTKSGIEKEKHT-PMVAPSSHHS-----  163
S. lycopersicum      -EEDDFTVP-----------IFVNSKLGQAHGSHNVNMEKLSPSGQLFCPNK--------  155
Z. mays              -NDDDFTVP-----------SVLYSGMPPHSSQEKLTLFPTTSPCKSVPAKYSSTDKR--  204
                      ....: :*           ....:.  .........  . .   ....:.

C. reinhardtii       GGGTGSPPSGGRRLAGPAHPAGAAGVRADGYKRRRDDWEEEEEVGPPHCAKALEAAAVAA  597
O. sativa            KSSTKCYNTVSKKL---------ERIHVSDVKSRTPLKDKEMEAAQTSKN----------  255
A. thaliana          --------I---RF---------QEVNQTGSKQNVCLATCSKP--EVRDQ----------  191
S. lycopersicum      ------------EL---------EGVTHLTLRQQRNSQNKE----NLKCT----------  180
Z. mays              ------------RL---------EGMDASDVKSKGPSGIKEKEPVQVRIN----------  233
                                 ::         ..:   . :..     .:    ... .
```

```
C. reinhardtii        GATPGGPLDGSETSGGLASGGGGGVTGADGNGSGGSTTLGAPAQRRSLSDEVAAARAARA  657
O. sativa             ---------VEVEKSSSFH------------------------------ASKDMFESRHAKV  278
A. thaliana           -------VKANARSGGFV------------------------------ISLDVSVTEEIDL  215
S. lycopersicum       -------LARREKTTSN------------------------------------SASKECRL  198
Z. mays               -------LEDKETTPSFQ------------------------------VLNDKTWSPDPKL  257
                             .   :.:.:.                               . . . ... ..

C. reinhardtii        EDARQQQRQRAGRSGTSDAGSGSRENASSGMRDVQLAEGGSNGGAAAGSDAMQVDMRWQQ  717
O. sativa             Y-------PKMDKTGIINDSDEPH------------------------------------  295
A. thaliana           E-------KSASSHDRVNDYNASL------------------------------------  232
S. lycopersicum       D-------PQVGCSSIPEPVKGTY------------------------------------  215
Z. mays               S-------SHMDRLKKQHAEAESY------------------------------------  274
                      .       ..... .  ..  ...

C. reinhardtii        TQAPTAAQAQTQAQQAGRGSAGSAGGGRGEVRGYGREAGRRGEPDTDMAEAEGEGEGQSE  777
O. sativa             -------------------------GGNSGHQATSRNGGS--------------------  310
A. thaliana           -------------------------RQESRNRLY-RDGGK--------------------  246
S. lycopersicum       -------------------------DGSS----YPRKEFV--------------------  226
Z. mays               -------------------------------QIRTRNENA--------------------  283
                                                . .  . . *...

C. reinhardtii        TECSDTPLTSSSSQSQSQSQQRGAASQRQQHQGGPAGMQAGGRGFREGEGEGGRMGASPL  837
O. sativa             MKFQNPPMRRNEISSNPSSEN------TDRHYNLPQG------GIEETGTKRKRLLEQHD  358
A. thaliana           TRLK---DTDNGAESHLATEN-----HSQEGHGSPED------IDNDREYSKSR------  286
S. lycopersicum       SEEQ---LTANDLVNDTESQE------DRAHKSLQTG-----------------------  254
Z. mays               VETQSPPKNGVSLLSKPYVDR------EQNGDSDLLG-----HGLRETGEKRKR------  326
                      .. .    . ..  :.. ...       . . . . :         .      .

C. reinhardtii        PASEAAVGAAGADDGVARAGGGSRDGSGGVAFPTRGNDGTTAAAAAAVTAAAAGLSLRAA  897
O. sativa             AEKSDDVSRLLEQHDA-----------------ENIDDVSDSS-VECI------------  388
A. thaliana           ------ACASLQQINE-----------------EASDDVSDDSMVDSI------------  311
S. lycopersicum       --------------NL-----------------DRGDDLSETSRVESI------------  271
Z. mays               -----------SHHDV-----------------EQNDDLSDSS-VESL------------  345
                                    ..                 .. :*.:..: :..:

C. reinhardtii        PPQPWGEQAQLPPQFQPHQLQHQQHQQPMFSPPPPGGHMMMPPPPAFPPGAGPVGPGGPL  957
O. sativa             -------------------------------------------------------TGWEI  393
A. thaliana           -------------------------------------------------------SSIDV  316
S. lycopersicum       -------------------------------------------------------SGTDI  276
Z. mays               -------------------------------------------------------PGMEI  350
                                                                             .: .:

C. reinhardtii        PPPPPGPPAPAGGPALRDLLGDPLYGAVRATLMRQQSVFVMQLGELHKIARVQQVIWSEM  1017
O. sativa             SP-----------DKIVGAIGTKHFWKARRAIMNQQRVFAVQVFELHKLVKVQKLI----  438
A. thaliana           SP-----------DDVVGILGQKRFWRARKAIANQQRVFAVQLFELHRLIKVQKLI----  361
S. lycopersicum       SP-----------DDIVGIIGLKRFWKARRAIVNQQRVFAIQVFELHRLIKVQRLI----  321
Z. mays               SP-----------DDVVSAIGPKHFWKARRAIVNQQRVFAVQVFELHRLIKVQKLI----  395
                      :*           :.....:* :.::.:*.::.:**:**::*:.***::.:**::*
                                                    Block II

C. reinhardtii        LVVDPAGLQAACMEAGFAGAGPAGPILRLPPPPAPGQQQQQQQQQRPLTPQPHMGLGTAT  1077
O. sativa             ---------------------AASPHVLIESDPCLGNALLGSK-NK--------------  462
A. thaliana           ---------------------AASPDLLLDEISFLGKVSAKSYPVKKL------------  388
S. lycopersicum       ---------------------AGSPNSSLEDPAYLGKPLKSSSIKR--------------  346
Z. mays               ---------------------AASPHVLIEGDPCLGKSLAVSK-KR--------------  419
                            Block II     :::*  ..:.  ..:*. .  :   :

C. reinhardtii        PESQEGGGVAAGEQQHQRQRPGLASVAGAAEAGYQLTSATTAHEAQAGSVVASAAVEAAG  1137
O. sativa             ------------------------------------------------------------  462
A. thaliana           ------------------------------------------------------------  388
S. lycopersicum       ------------------------------------------------------------  346
Z. mays               ------------------------------------------------------------  419

C. reinhardtii        GYGGGAGTSSQPHHGAQRSAPAAPPPPAAPPLSREELRVRLLGDMQLLFNLPRGLRERVL  1197
O. sativa             ------------------------------------------------------------  462
A. thaliana           ------------------------------------------------------------  388
S. lycopersicum       ------------------------------------------------------------  346
Z. mays               ------------------------------------------------------------  419
```

```
C. reinhardtii       RSVPPEQLVRPAVPPPPAPRPAATMVGGGGGLGGGGNAVVRPAASKLYTLMDGDEGPADS  1257
O. sativa            --LVEENL--KAQPLLVATI--------------------------------------DD  480
A. thaliana          --LPSEFLVKPPLPHVVVKQ------------------------------------RGDS  410
S. lycopersicum      --LPLDCIVRESQSVLKRKH--------------------------------------DS  366
Z. mays              --LAGD-V--ETQLESAKND--------------------------------------DG  436
                       :.  :  :  .... .  .                                        *.

C. reinhardtii       EQPTVVQKVVAQATARAAAAAAEKQRNNGGSGGGSVAGGPNGGIPAAVGQAGPFRQQPTA  1317
O. sativa            VEPSLQQPEVSKENTEDSPPSPHDTGLGSGQRDQAATNGVSKSNRRATPVASDNKQNNWG  540
A. thaliana          EKTDQHKMESSAENVVGRLSN--------QGHHQQSNYMPFANNPPASPAPNGY-----C  457
S. lycopersicum      EKPH-FRMEHTAESNVGKASLSTV-----QNGSQLSSHKPFSGTPLPTPVTNDSNAGPWC  420
Z. mays              VRP--KQLEHSKEKTEANQPSP-------SQDEQAATNGAVAASMHT---PSDNKQKSWC  484
                     ..:   : : ..:.. . ..         ... : .. .. .... .   ...  .   .

C. reinhardtii       APPSPHAAQPATRLGLPDGAAAAVPSPHQVLPPFPPHGMPQRPQASALLMPPPSMHPHMH  1377
O. sativa            VQLQP----PQNQWLVP----VMSPLEGLVYKPYSGPC----PPAGSILA-P--------  579
A. thaliana          FPPQPPPSGNHQQWLIP----VMSPSEGLIYKPHPGMA----------HTGH--------  495
S. lycopersicum      FQ-QP----PGHQWLIP----VMSPSEGLVYKPFPGPG----------FTSP--------  453
Z. mays              IP-AP----PS-QWLIP----VMSPSEGLVYKPYTGHC----PPVGSLLAPP--------  522
                     .. .*    :  ::.:*    :::*:::::::*:.:..          .. :
                                   Block III
C. reinhardtii       PPSPAAAFGLPPHALPAGPGLAQPHALLGGMPPAAAAAAGLAVGPPPHGMGAAGAAAAAT  1437
O. sativa            --------------------------FYANCTPLSLPSTAGDFMNSAYG-----------  602
A. thaliana          --------------------------YGGYY---------GHYMPTPMV-----------  509
S. lycopersicum      --------------------------ICGSGPPGSSPTM-GNFFAPTYG-----------  475
Z. mays              --------------------------FFASYPTSSSSTAGGDFMSSACG-----------  545
                                               . :  .. .     ........:

C. reinhardtii       AGAGAFGSPGSSFRAPPHPAQPGQPLHMDLPPQWSGPAMPQHGHLPLHGQLPPHMHMAMP  1497
O. sativa            ----------------------------------------------------------VP  604
A. thaliana          ----------------------------------------------------------MP  511
S. lycopersicum      ----------------------------------------------------------VP  477
Z. mays              ----------------------------------------------------------AR  547
                                                                               .:

C. reinhardtii       LHGQLPPGMAGPLGALPARGPLQGGAPGAPPPGGAPMAVAAGGPRGPLQQPQQPQPLVRP  1557
O. sativa            MPHQ-PQHM---------------GAPGPPSMPMNYF-----------------------  625
A. thaliana          QYHP---GM---------------GFP---PPGNGYF-----------------------  527
S. lycopersicum      APNPHYQGM---------------GVPFAPPTGHGYF-----------------------  499
Z. mays              L-------M---------------SAP-------VYF-----------------------  555
                     .      .*               :.*   . . ..:

C. reinhardtii       PGVPPPLVGTPPLP-MPQPGPSAAAPVPHLDAAGTSQPQSAAAPAPTPAAPVPGGGFGGV  1616
O. sativa            ----------PPFS-IPVMNPTAPAPV--------------VEQGRHPSMPQPYGNF---  657
A. thaliana          ----------PPYGMMPTIMNPYCSSQ--------------QQQQQQPNEQMNQFGHPGN  563
S. lycopersicum      ----------RQYG-MPAMNPPISSTA--------------SEESNQYTMPGLQHQFSGV  534
Z. mays              ----------PSFS-MPAVSGSA------------------VEQVSHVAASQHKRN----  582
                               :... :*.. ...                   ..  .....    ..

C. reinhardtii       LPPALPVLFSSRQGAGVGAGGTKVPPVLRPPGVPAPAIPVPRPVASIDPHEAWFARHVAG  1676
O. sativa            ------------------------------------------------------------  657
A. thaliana          L-----------------------------------------------------------  564
S. lycopersicum      V-----------------------------------------------------------  535
Z. mays              ------------------------------------------------------------  582

C. reinhardtii       AGAGAQPAAAQPAAATPAAAAEAAGAAGPAAGAVGSRQAQAQAPSQGLGSRPGSLQRIAS  1736
O. sativa            ------------------------------------------------------------  657
A. thaliana          ------------------------------------------------------------  564
S. lycopersicum      ------------------------------------------------------------  535
Z. mays              ------------------------------------------------------------  582

C. reinhardtii       TERVVGGGGGSAAAVTGTGALQQPPERQSQPQQRQGAEEAEAAPSAARGGPSADVGWVAA  1796
O. sativa            --------------------------------------EQQSWISCNMSHPSGIWRFHAS  679
A. thaliana          ------------------------------QNTQQQQQRSDNEPAPQQQQQPTKS-YPRA  593
S. lycopersicum      ------------------------------DDVNIQHQDSSNVLNQKKENVPDVVRYQST  565
Z. mays              --------------------------------------------SCSEA-------VLAS  591
                                                                 .           . ..
```

```
C. reinhardtii      ARQGQPHQPRPASEMQAGAPQDQAPSASQPEGLEAQGQQELQTGAPGVAGAPAAPPDVYA  1856
O. sativa           RDS-----------------EAQASSASSPFDRFQ-------------------------  697
A. thaliana         RKS-----------------R-QGSTGSSPSGPQG-------------------------  610
S. lycopersicum     KDN-----------------EVQASSASSPIE----------------------------  580
Z. mays             RDS-----------------EVQGSSASS-------------------------------  603
                    ...                 . *::::*:.

C. reinhardtii      VAGGSAGGAPGQGQAQAASSDDDEEEDVATSAGANALQPPPSLPPLHQAEAAAGLAAAAA  1916
O. sativa           ------------------------------CSGSGPVSAFPTVS----------------  711
A. thaliana         ------------------------------ISGSKSFRPFAAVD----------------  624
S. lycopersicum     ------------------------------TAGRNMLSLFPTSP----------------  594
Z. mays             ------------------------------------------------------------  603
                                                    .       .

C. reinhardtii      AAPAAAAAAPTSSGAAAPPPPPPPSAAVPVPAVGPTKWWQDPNTAFGELGLVDPQALGNT  1976
O. sativa           --AQNNQPQPSYSSRDNQTN----------------------------------------  729
A. thaliana         -EDSNINNAPEQTMTTTTTTTRTTVTQTTRDGGGVTR-----------------------  660
S. lycopersicum     -VTDNRDGSPQACVPDNPAR----------------------------------------  613
Z. mays             ---------PASSETAAQPR----------------------------------------  614
                        .    *  . .....
                                                         Block IV

C. reinhardtii      PAVVEDDEEDDDDEAAAAAATAGLQPGDALAAHMQLVQSLVPAPRAAGSGEGSGSGAPPP  2036
O. sativa           ------------------------------------------------------------  729
A. thaliana         ------------------------------------------------------------  660
S. lycopersicum     ------------------------------------------------------------  613
Z. mays             ------------------------------------------------------------  614
                                         Block IV

C. reinhardtii      LPPLKKREKYMMQQQQQQQQQQAAAVAAAAAAAAGGAPPGAAPGQGAAALAGANQAAMAAA  2096
O. sativa           ------------------------------------------------------------  729
A. thaliana         ------------------------------------------------------------  660
S. lycopersicum     ------------------------------------------------------------  613
Z. mays             ------------------------------------------------------------  614
                                         Block IV

C. reinhardtii      AAVALGLDPATAAAAAALMASGVAGGLGPGGMGGGVRPEVLLAATRMLQPSAFKTVPPRQ  2156
O. sativa           ---------------------------------------------------VIKVVPHNS  738
A. thaliana         ---------------------------------------------------VIKVVPHNA  669
S. lycopersicum     ---------------------------------------------------VIKVVPHNA  622
Z. mays             ---------------------------------------------------VIRVVPHTA  623
                                         Block IV                  ::::**:..

C. reinhardtii      QHSNRGSNRLTMSTHTGATESMASGWGFGLSRRAGGGAQPERSEAGVSRATGGAGPHYRT  2216
O. sativa           RTASESAARIFRSI----------------------------------------------  752
A. thaliana         KLASENAARIFQSI----------------------------------------------  683
S. lycopersicum     RSATESVARIFQSI----------------------------------------------  636
Z. mays             RTASESAARIFRSI----------------------------------------------  637
                    : :.:..:*::.*:       Block IV

C. reinhardtii      SGNGGATTSAAGGGAGRPARKVERKYDGEASEAGGGAASAASGGRRPEAQQAQQRPQQQQ  2276
O. sativa           -----------------------------------------------------------Q  753
A. thaliana         -----------------------------------------------------------Q  684
S. lycopersicum     -----------------------------------------------------------Q  637
Z. mays             -----------------------------------------------------------Q  638
                                         Block IV                                  *

C. reinhardtii      QQRQQQQGARGGITAGGAAATGAEGTQGGAEADEAQTSGAGGGGGGAGGRAAGGAAGKVR  2336
O. sativa           MERQRDD-----------------------------------------------------  760
A. thaliana         EERKRYDSSKP-------------------------------------------------  695
S. lycopersicum     QERNNMT-----------------------------------------------------  644
Z. mays             MERKQNDP----------------------------------------------------  646
                    .:*.. .
                  Block IV

C. reinhardtii      RAGEGPSVAAAAAAGVGGAVRHPQQRAVARAGGGDDVGEVSSGAVEAAAEAEDGMSE  2392
O. sativa           --------------------------------------------------------  760
A. thaliana         --------------------------------------------------------  695
S. lycopersicum     --------------------------------------------------------  644
Z. mays             --------------------------------------------------------  646
```
