# Supplementary material for: A potential EARLY FLOWERING 3 homolog in Chlamydomonas is involved in the red/violet and blue light signaling pathways for the degradation of RHYTHM OF CHLOROPLAST 15
Source: PLoS Genet. 2022 Oct 17;18(10):e1010449. doi: 10.1371/journal.pgen.1010449 (PMC9612821; doi:10.1371/journal.pgen.1010449)
Supplement: S1 Table — (DOCX) [file pgen.1010449.s018.docx]

| Primer set | Primer name | Sequence (5’ --˃3’) | Target | Purpose |
| --- | --- | --- | --- | --- |
| I | UPI | CGCCGCCTCCAAGCTGTACACAC | Upstream of *aph7”* insertion (M1 (*cetl-1*)) | Insertion check in the *CETL* gene from *cetl-1* mutant. |
|  | DPI | GGTGCATGTGCGGGTGCATACTGG | Downstream of *aph7”* insertion (M1(*cetl-1*)) |  |
| II | m19-F | AGCCGAGGCCCGCGTCGGAAATG | Upstream of *aph7”* insertion (*b19* (*cetl-2*)) | Insertion check in the *CETL* gene from *cetl-2* mutant. |
|  | m19-R | GGCAGTAGCGGTCAGGGCGGTG | Downstream of *aph7”* insertion (*b19* (*cetl-2*)) |  |
| III | RTP4(Ι) | GCGCGGGTGCAACAGGTCATCTGGTC | First exon junction of the *CETL* transcript | Insertion check in the *CETL* transcript from the *cetl-1* mutant. |
|  | RTP3(ΙΙ) | GGGCGGCGGCATAAGGAGAGCTGAC | Exon immediately downstream of insertion locus in the *CETL* transcript |  |
| IV | qPCR1 | TGGACCCACAGGCCCTGGGCAACAC | Fifth and seventh exon junction of the *CETL* transcript  (Cre07.g357500) | Quantification of *CETL* mRNA. |
|  | qPCR2 | TGCCGGGGGGGCACCGTCTTGAATG |  |  |

**Primer list**

**S1 Table.** Details of primers used for insertion check and RT-qPCR.
