# Supplementary material for: A potential EARLY FLOWERING 3 homolog in Chlamydomonas is involved in the red/violet and blue light signaling pathways for the degradation of RHYTHM OF CHLOROPLAST 15
Source: PLoS Genet. 2022 Oct 17;18(10):e1010449. doi: 10.1371/journal.pgen.1010449 (PMC9612821; doi:10.1371/journal.pgen.1010449)
Supplement: S2 Table — (DOCX) [file pgen.1010449.s019.docx]

**Sequences used for alignment**

| S. No | Genome | Repository/portal | Protein ID | Transcript ID | Supporting references |
| --- | --- | --- | --- | --- | --- |
| 1. | *Chlamydomonas reinhardtii* CBR strain | DDBJ/GenBank/EMBL-EBI |  | LC664050 |  |
| 2. | *Chlamydomonas incerta* SAG 7. 73 | PhycoCosm | 16158 | 16158 | [1] |
| 3. | *Chlamydomonas schloesseri* CCAP 11/173 | PhycoCosm | 12441 | 12441 |  |
| 4. | *Edaphochlamys debaryana* CCAP 11/70 | PhycoCosm | 11184 | 11184 |  |
| 5. | *Volvox carteri* v2.1 | PhycoCosm | 12823 | 12823 | [2] |
| 6. | *Gonium pectorale* NIES- 2863 | PhycoCosm | 14005 | 14005 | [3] |
| 7. | *Gonium pectorale* NIES- 2863 | PhycoCosm | 14004 | 14004 |  |
| 8. | *Chlamydomonas eustigma* NIES-2499 | PhycoCosm | 8338 | 8338 | [4] |
| 9. | *Dunaliella salina* CCAP 19/18 | PhycoCosm | 13574 | 13574 | [5] |
| 10. | *Chromochloris zofingiensis* v5.2.3.2 | Phytozome |  | Cz15g15170.t1 | [6] |
| 11. | *Raphidocelis subcapitata* NIES-35 | PhycoCosm | 7428 | 7428 | [7] |
| 12. | *Enallax costatus* CCAP 276/31 v1.1 | PhycoCosm | 6729302 | [6730092](https://phycocosm.jgi.doe.gov/annotator/servlet/jgi.annotation.Annotation?pDb=Enacos1_1&pStateVar=View&pProteinId=6729302&pViewType=protein) | [8] |
| 13. | *Scenedesmus Sp.* NREL 46B-D3 v1.0 | PhycoCosm | 1642377 | 1643679 | [9] |
| 14. | *Tetradesmus obliquus* UTEX B 72 v1.1 | PhycoCosm | 1123993 | 1124339 | [8] |
| 15. | *Asterochloris glomerata* Cgr/DA1 pho v2.0 | PhycoCosm | 5319 | 5319 | [10] |
| 16. | *Bortryococcus braunii* Showa v2.1 | PhycoCosm | 20297 | 20297 | [11] |
| 17. | *Symbiochloris reticulata* Spain reference genome v1.0 | PhycoCosm | 838596 | 838660 | [8] |
| 18. | *Picochlorum renovo* | PhycoCosm | 29125 | 29169 | [12] |
| 19. | *Micractinium conductrix* SAG 241.80 | PhycoCosm | 5015 | 5015 | [13] |
| 20. | *Chlorella variabilis* NC64A | PhycoCosm | 52115 | 52115 | [14] |
| 21. | *Chlorella sorokiniana* UTEX 1602 | PhycoCosm | 5130 | 5130 | [13] |
| 22. | *Micromonas commoda* NOUM17(RCC299) | PhycoCosm | 7934 | 7934 | [15] |
| 23. | *Klebsormidium nitens* NIES-2285 | *Klebsormidium nitens* NIES-2285 genome project | kfl00240_0090_v1.1 |  | [16] |
| 24. | Chara braunii | ORCAE | g6559.t1 |  | [17] |
| 25. | *Marchantia polymorpha* v3.1 | Phytozome |  | Mapoly0014s0139.1 | [18] |
| 26. | *Physcomitrium patens* v3.3 | Phytozome |  | Pp3c11_14750V3.4 | [19] |
| 27. | *Selaginella moellendorfii* v1.0 | Phytozome |  | 415241 | [20] |
| 28. | *Selaginella moellendorfii* v1.0 | Phytozome |  | 411196 |  |
| 29. | *Sorghum bicolor* | PhycoCosm | 5010261 | 5239448 | [21] |
| 30. | *Oryza sativa* | rap-db | Os01t0566100-02 |  | [22] |
| 31. | *Oryza sativa* | rap-db | Os06t0142600-01 |  |  |
| 32. | *Populus trichocarpa* v4.1 | Phytozome |  | Potri.006G233800.1 | [23] |
| 33. | *Arabidopsis thaliana* | TAIR | AT2G25930.1 |  | [24] |
| 34. | *Solanum lycopersicum* ITAG4.0 | Phytozome |  | Solyc08g065870.4.1 | [25] |
| 35. | *Zea mays* B84 v1.2 | Phytozome |  | ZmB84.06G283200.1.p | [26] |

**S2 Table.** Accession details for the sequences used in the protein sequence alignment (**Figs 2B, 2C, S5, and S6**).

**Supplementary References**

1. Craig RJ, Hasan AR, Ness RW, Keightley PD. Comparative genomics of *Chlamydomonas*. Plant Cell. 2021;33: 1016–1041. DOI: [10.1093/plcell/koab026](https://doi.org/10.1093/plcell/koab026).
2. Prochnik SE, Umen J, Nedelcu AM, Hallmann A, Miller SM, Nishii I, et al. Genomic Analysis of Organismal Complexity in the Multicellular Green Alga *Volvox carteri*. Science. 2010; Volume 329: 223–226. DOI: [10.1126/science.1188800](https://doi.org/10.1126/science.1188800).
3. Hanschen ER, Marriage TN, Ferris PJ, Hamaji T, Toyoda A, Fujiyama A, et al. The *Gonium pectorale* genome demonstrates co-option of cell cycle regulation during the evolution of multicellularity. Nat Commun. 2016;7: 11370. DOI: [10.1038/ncomms11370](https://doi.org/10.1038/ncomms11370).
4. Hirooka S, Hirose Y, Kanesaki Y, Higuchi S, Fujiwara T, Onuma R, et al. Acidophilic green algal genome provides insights into adaptation to an acidic environment. Proc Natl Acad Sci U S A. 2017; Volume 114: E8304–E8313. DOI: [10.1073/pnas.1707072114](https://doi.org/10.1073/pnas.1707072114).
5. Polle JEW, Barry K, Cushman J, Schmutz J, Tran D, Hathwaik LT, et al. Draft Nuclear Genome Sequence of the Halophilic and Beta-Carotene-Accumulating Green Alga *Dunaliella salina* Strain CCAP19/18. Genome Announc. 2017;5. doi:[10.1128/genomeA.01105-17](https://doi.org/10.1128/genomea.01105-17).
6. Roth MS, Cokus SJ, Gallaher SD, Walter A, Lopez D, Erickson E, et al. Chromosome-level genome assembly and transcriptome of the green alga *Chromochloris zofingiensis* illuminates astaxanthin production. Proc Natl Acad Sci U S A. 2017;114: E4296–E4305. DOI: [10.1073/pnas.1619928114](https://doi.org/10.1073/pnas.1619928114).
7. Suzuki S, Yamaguchi H, Nakajima N, Kawachi M. Raphidocelis subcapitata (=Pseudokirchneriella subcapitata) provides an insight into genome evolution and environmental adaptations in the Sphaeropleales. Sci Rep. 2018; Volume 8: 8058. DOI: 10.1038/s41598-018-26331-6.
8. Grigoriev IV, Hayes RD, Calhoun S, Kamel B, Wang A, Ahrendt S, et al. PhycoCosm, a comparative algal genomics resource. Nucleic Acids Res. 2021;49: D1004–D1011. DOI: [10.1093/nar/gkaa898](https://doi.org/10.1093/nar/gkaa898).
9. Calhoun S, Bell TAS, Dahlin LR, Kunde Y, LaButti K, Louie KB, et al. A multi-omic characterization of temperature stress in a halotolerant *Scenedesmus* strain for algal biotechnology. Commun Biol. 2021;4: 333. DOI: [10.1038/s42003-021-01859-y](https://doi.org/10.1038/s42003-021-01859-y).
10. Armaleo D, Müller O, Lutzoni F, Andrésson ÓS, Blanc G, Bode HB, et al. The lichen symbiosis re-viewed through the genomes of *Cladonia grayi* and its algal partner *Asterochloris glomerata*. BMC Genomics. 2019;20: 605. DOI: [10.1186/s12864-019-5629-x](https://doi.org/10.1186/s12864-019-5629-x).
11. Browne DR, Jenkins J, Schmutz J, Shu S, Barry K, Grimwood J, et al. Draft Nuclear Genome Sequence of the Liquid Hydrocarbon-Accumulating Green Microalga *Botryococcus braunii* Race B (Showa). Genome Announc. 2017;5. doi:[10.1128/genomeA.00215-17](https://doi.org/10.1128/genomea.00215-17).
12. Dahlin LR, Gerritsen AT, Henard CA, Van Wychen S, Linger JG, Kunde Y, et al. Development of a high-productivity, halophilic, thermotolerant microalga *Picochlorum renovo*. Commun Biol. 2019;2: 388. DOI: [10.1038/s42003-019-0620-2](https://doi.org/10.1038/s42003-019-0620-2).
13. Arriola MB, Velmurugan N, Zhang Y, Plunkett MH, Hondzo H, Barney BM. Genome sequences of *Chlorella sorokiniana* UTEX 1602 and *Micractinium conductrix* SAG 241.80: implications to maltose excretion by a green alga. Plant J. 2018;93: 566–586. DOI: [10.1111/tpj.13789](https://doi.org/10.1111/tpj.13789).
14. Blanc G, Duncan G, Agarkova I, Borodovsky M, Gurnon J, Kuo A, et al. The *Chlorella variabilis* NC64A genome reveals adaptation to photosymbiosis, coevolution with viruses, and cryptic sex. Plant Cell. 2010;22: 2943–2955. DOI: [10.1105/tpc.110.076406](https://doi.org/10.1105/tpc.110.076406).
15. Worden AZ, Lee JH, Mock T, Rouzé P, Simmons MP, Aerts AL, et al. Green evolution and dynamic adaptations revealed by genomes of the marine picoeukaryotes *Micromonas*. Science. 2009;324: 268–272. DOI: [10.1126/science.1167222](https://doi.org/10.1126/science.1167222).
16. Hori K, Maruyama F, Fujisawa T, Togashi T, Yamamoto N, Seo M, et al. *Klebsormidium flaccidum* genome reveals primary factors for plant terrestrial adaptation. Nat Commun. 2014;5: 3978. DOI: [10.1038/ncomms4978](https://doi.org/10.1038/ncomms4978).
17. Nishiyama T, Sakayama H, de Vries J, Buschmann H, Saint-Marcoux D, Ullrich KK, et al. The *Chara* Genome: Secondary Complexity and Implications for Plant Terrestrialization. Cell. 2018;174: 448–464.e24. DOI: [10.1016/j.cell.2018.06.033](https://doi.org/10.1016/j.cell.2018.06.033).
18. Bowman JL, Kohchi T, Yamato KT, Jenkins J, Shu S, Ishizaki K, et al. Insights into Land Plant Evolution Garnered from the *Marchantia polymorpha* Genome. Cell. 2017;171: 287–304.e15. DOI: [10.1016/j.cell.2017.09.030](https://doi.org/10.1016/j.cell.2017.09.030).
19. Lang D, Ullrich KK, Murat F, Fuchs J, Jenkins J, Haas FB, et al. The *Physcomitrella patens* chromosome-scale assembly reveals moss genome structure and evolution. Plant J. 2018;93: 515–533. DOI: [10.1111/tpj.13801](https://doi.org/10.1111/tpj.13801)
20. Banks JA, Nishiyama T, Hasebe M, Bowman JL, Gribskov M, dePamphilis C, et al. The *Selaginella* genome identifies genetic changes associated with the evolution of vascular plants. Science. 2011;332: 960–963. DOI: [10.1126/science.1203810](https://doi.org/10.1126/science.1203810).
21. Paterson AH, Bowers JE, Bruggmann R, Dubchak I, Grimwood J, Gundlach H, et al. The *Sorghum bicolor* genome and the diversification of grasses. Nature. 2009;457: 551–556. DOI: [10.1038/nature07723](https://doi.org/10.1038/nature07723).
22. Sakai H, Lee SS, Tanaka T, Numa H, Kim J, Kawahara Y, et al. Rice Annotation Project Database (RAP-DB): An Integrative and Interactive Database for Rice Genomics. Plant Cell Physiol. 2013;54: e6–e6. DOI: [10.1093/pcp/pcs183](https://doi.org/10.1093/pcp/pcs183).
23. Tuskan GA, Difazio S, Jansson S, Bohlmann J, Grigoriev I, Hellsten U, et al. The genome of black cottonwood, *Populus trichocarpa* (Torr. & Gray). Science. 2006;313: 1596–1604. DOI: [10.1126/science.1128691](https://doi.org/10.1126/science.1128691).
24. Berardini TZ, Reiser L, Li D, Mezheritsky Y, Muller R, Strait E, Huala E. The Arabidopsis information resource: making and mining the “gold standard” annotated reference plant genome. genesis. 2015; 53: 474-85.DOI: [10.1002/dvg.22877](https://doi.org/10.1002/dvg.22877).
25. Hosmani PS, Flores-Gonzalez M, van de Geest H, Maumus F, Bakker LV, Schijlen E, et al. An improved *de novo* assembly and annotation of the tomato reference genome using single-molecule sequencing, Hi-C proximity ligation and optical maps. Cold Spring Harbor Laboratory. 2019. doi:10.1101/767764
26. Bornowski N, Michel KJ, Hamilton JP, Ou S, Seetharam AS, Jenkins J, et al. Genomic variation within the maize stiff-stalk heterotic germplasm pool. Plant Genome. 2021;14: e20114. doi:10.1002/tpg2.20114
